# Targeting protein modifications in metabolic diseases: molecular mechanisms and targeted therapies

**DOI:** 10.1038/s41392-023-01439-y

**Published:** 2023-05-27

**Authors:** Xiumei Wu, Mengyun Xu, Mengya Geng, Shuo Chen, Peter J. Little, Suowen Xu, Jianping Weng

**Affiliations:** 1 Department of Endocrinology, Institute of Endocrine and Metabolic Diseases, The First Affiliated Hospital of USTC, Division of Life Sciences and Medicine, Clinical Research Hospital of Chinese Academy of Sciences (Hefei), University of Science and Technology of China, Hefei, Anhui 230001 China; 2 Department of Endocrinology and Metabolism, Guangdong Provincial Key Laboratory of Diabetology, The Third Affiliated Hospital of Sun Yat-sen University, 510000 Guangzhou, China; 3 School of Pharmacy, University of Queensland, Pharmacy Australia Centre of Excellence, Woolloongabba, QLD 4102 Australia; 4 Sunshine Coast Health Institute and School of Health and Behavioural Sciences, University of the Sunshine Coast, Birtinya, QLD 4575 Australia; 5 Bengbu Medical College, Bengbu, 233000 China

**Keywords:** Metabolic disorders, Biochemistry

## Abstract

The ever-increasing prevalence of noncommunicable diseases (NCDs) represents a major public health burden worldwide. The most common form of NCD is metabolic diseases, which affect people of all ages and usually manifest their pathobiology through life-threatening cardiovascular complications. A comprehensive understanding of the pathobiology of metabolic diseases will generate novel targets for improved therapies across the common metabolic spectrum. Protein posttranslational modification (PTM) is an important term that refers to biochemical modification of specific amino acid residues in target proteins, which immensely increases the functional diversity of the proteome. The range of PTMs includes phosphorylation, acetylation, methylation, ubiquitination, SUMOylation, neddylation, glycosylation, palmitoylation, myristoylation, prenylation, cholesterylation, glutathionylation, S-nitrosylation, sulfhydration, citrullination, ADP ribosylation, and several novel PTMs. Here, we offer a comprehensive review of PTMs and their roles in common metabolic diseases and pathological consequences, including diabetes, obesity, fatty liver diseases, hyperlipidemia, and atherosclerosis. Building upon this framework, we afford a through description of proteins and pathways involved in metabolic diseases by focusing on PTM-based protein modifications, showcase the pharmaceutical intervention of PTMs in preclinical studies and clinical trials, and offer future perspectives. Fundamental research defining the mechanisms whereby PTMs of proteins regulate metabolic diseases will open new avenues for therapeutic intervention.

## Introduction

Rapid economic development, ageing of the population, and evolved lifestyles have the outcome of creating a dramatic worldwide growth in chronic metabolic disorders. These preventable lifestyle-related diseases include hyperglycemia, hyperlipidemia, hypertension, obesity and its consequence, nonalcoholic fatty liver disease (NAFLD).

The 10^th^ International Diabetes Federation (IDF) indicates a global diabetes prevalence of nearly 10% (537 million), and the cases are predicted to reach 783 million by 2045; 90 percent of these cases are type 2 diabetes.^1^ The global prevalence of NAFLD is 38%, growing to nonalcoholic steatohepatitis (NASH) and hepatocellular carcinoma.^2^ As of 2015, nearly 712 million individuals (604 million adults, 108 million children) were obese worldwide, and the prevalence of obesity more than doubled from 1.1% in 1980 to 3.85% in 2015. Childhood obesity had an even higher rate of increase.^3^ The seminal issue is that metabolic disorders are closely related to the consequences of cardiovascular diseases (CVDs) and all-cause mortality.^4^ Over the past decades, CVD cases have increased from 271 million in 1990, reaching 523 million in 2019. The total cardiovascular deaths rose to 18.6 million in 2019, up from 12.1 million in 1990.^5^ CVD has become the predominant contributor to global mortality and disability.^5,6^ Alarmingly, the occurrence and hospitalization for metabolic disorders and CVD in young adults are increasing.^5,6^

With the rising incidence of metabolic diseases and CVD, attention has been focused on the global cardiometabolic disease epidemic, with negative impacts on lifespan and socioeconomic burden. In America, 90% of the annual healthcare expenditures (3.7 trillion dollars) are directed to the population with chronic diseases and mental health issues.^7^ Metabolic diseases represent both a huge social burden but also provide an opportunity for high cost-effectiveness for efficacious interventions. Since most metabolic diseases are preventable and treatable, their prevention and control will yield great societal and economic benefits.

The Human Genome Project has revealed the human genome includes about 20,000 to 25,000 genes, whereas, due to alternative splicing, metabolism and PTMs, the human proteome includes over 1 million proteins. Posttranslational modifications (PTMs) are central to the complexity and diverse functional roles of the proteome. PTMs are the biochemical modifications of proteins after protein biosynthesis. PTMs dynamically regulate protein activity, location and molecular interactions by modifying or introducing functional groups such as phosphoryl, methyl, acetyl and glycosyl groups. PTMs generally occur in proteins serving as important structures or exhibiting crucial functions, such as histones, membrane proteins and secretory proteins. PTMs are usually reversible, and the irreversible alterations arise from proteolytic modifications. PTMs take place in various cellular compartments, such as nucleus, cytosol, endoplasmic reticulum (ER) and the Golgi complex.^8^

Protein phosphorylation was the first discovered PTM and this phenomena was first identified in 1906.^9^ Another 50 years passed before the specific observation of protein kinase activity in 1954.^10^ In the 1960s, the general importance of PTMs was appreciated, as the discovery of histone acetylation governing the transcription of genes was put forward in 1964^11^ and protein phosphorylation participating in cell metabolism was reported in 1969.^12^ The biological relevance of the newborn field of PTM sparked much excitement across scientific communities. However, the investigation of PTMs had decades of stagnation because of poor PTM detection technology and a lack description of functional activity and consequences. Fortunately, the enhanced accessibility of genomic sequencing data and the rapid development of detection approaches such as mass spectrometry (MS)-based proteomics, radioactive isotope labeling, peptide/protein array, immunoprecipitation and proximity ligation assay (PLA) have ended the long struggle and led to a golden age of PTM research.^13^

To date, owing to advanced detection technologies over 600 types of PTMs have been identified.^13^ These PTMs affect enzyme function and assembly, receptor activation, protein interactions, cell interactions, protein solubility, molecular trafficking, protein stability, protein folding, protein turnover, protein localization, cell metabolism, and signaling pathways.^14^ The most general PTMs include protein phosphorylation, methylation, acetylation, SUMOylation, neddylation, ubiquitination, glycosylation, palmitoylation, glutathionylation, S-nitrosylation, and ADP ribosylation. Aberrant PTMs are implicated in diverse human diseases, including metabolic disorders and CVDs.

Due to the aberrant regulatory role of PTMs in diseases, multiple important therapeutic agents regulating PTMs, such as kinase agonists/inhibitors, histone deacetylase inhibitors, and histone methyltransferase inhibitors, are discovered to treat a variety of illnesses. The c-Abl tyrosine kinase inhibitor, imatinib (Glevec^®^), which received Food and Drug Administration (FDA) approval in 2001, was the first “smart” kinase inhibitor developed with a specific target identified to treat chronic myeloid leukemia.^15^ This remarkable progress arouses awareness of the significance of protein phosphorylation, allowing protein kinases to serve as the second most prominent drug target category, following G-protein-coupled receptors.^16^ Drugs targeting PTMs have thus provided potential therapeutic strategies in the study of diverse diseases, including metabolic disorders.^17–19^

**Fig. 1:**
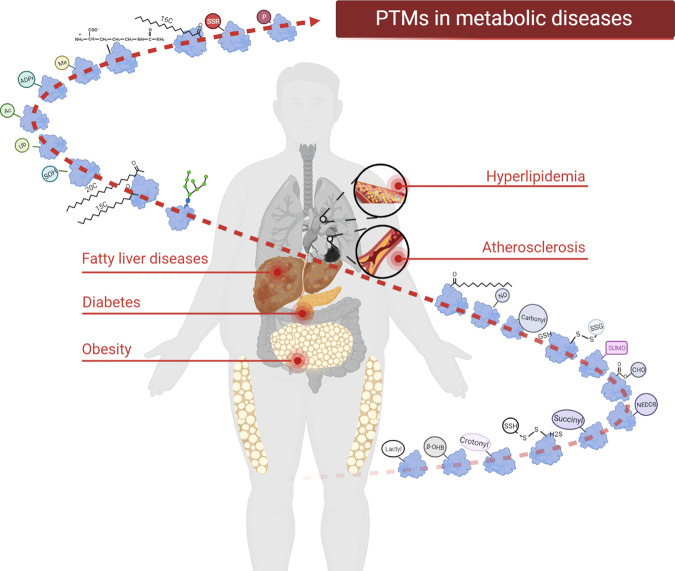
PTMs in metabolic diseases. An increasing number of people are suffering from metabolic diseases, such as fatty liver, diabetes, obesity, hyperlipidemia and atherosclerosis. The liver, pancreas, adipose tissue, blood vessels, and heart are the main affected organs. Numerous proteins and PTMs (such as phosphorylation, acetylation, methylation, ubiquitination, etc.) are involved in the normal biology of these organs and the whole body. Abnormal PTMs thus are involved in the progression of these metabolic diseases and can be therapeutically targeted. The figure is generated with BioRender (https://biorender.com)

In summary, as shown in Fig. 1, an increasing number of human subjects acquire metabolic diseases which occurred in the liver (fatty acid liver), pancreas (diabetes), adipose tissue (obesity), blood fat (hyperlipidemia) and heart (atherosclerosis). Numerous proteins and PTMs are implicated in the progression of these metabolic diseases. To put into practice the preventive and treatment options for metabolic diseases, both medical and lifestyle, a thorough understanding of PTMs and metabolic disorders is needed. Here, we systematically review and profile the most recent advances in the roles of PTMs in metabolic diseases.

## Principles and mechanisms of protein posttranslational modifications (PTMs)

**Fig. 2:**
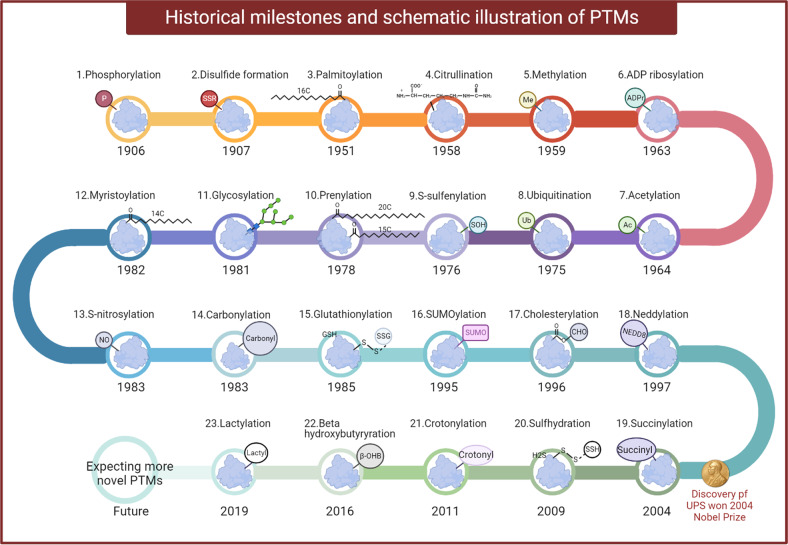
The historical milestones and schematic illustration of different PTMs. Protein phosphorylation was the first discovered PTM and this phenomenon was first identified in 1906. Since then, other common PTMs such as disulfide formation, palmitoylation, citrullination, methylation, ADP ribosylation, etc were being discovered. Recently, a novel PTM, lactylation, was reported in 2019. Advanced availability of genomic sequence information and the rapid development of detection approaches will lead to a golden age of PTM research and afford abundant novel therapeutic targets for human diseases. The figure is generated with BioRender (https://biorender.com). ADPr adenosine diphosphate (ADP)-ribose, Ac the acetyl group, β-OHB β-hydroxybutyrate, CHO cholesterol, GSH glutathione, H2S hydrogen sulfide, Me the methyl group, NEDD8 neuronal precursor cell-expressed developmentally downregulated protein 8, NO nitric oxide, P the phosphate group, SOH sulfenic acid, SSG glutathione disulfide, SSH the persulfide group, SSR disulfide formation, Ub ubiquitin, PTMs post translational modifications

Transcription, translation, and PTMs provide a multilayer dynamic network for biochemical and physiological diversity and complexity. Dynamic reversibility enables PTMs to regulate cellular processes and signal transduction most efficiently. One protein can be modified by various PTMs at a time or modified by one specific PTM at different stages. Figure 2 illustrates the historical milestones of PTMs research. It can be seen that since the discovery of first type of identified PTM (phosphorylation in 1906^9^) it takes more than one hundred years to the latest discovery of lactylation in 2019.^20^ Advances in detection technology will provide researchers with more opportunities to explore the biological functions of novel PTMs and accelerate the research of the roles of PTMs in human diseases, which could hold the promise of novel therapies. Below, we will describe the principles and mechanisms of the most common PTMs.

### Phosphorylation

Protein phosphorylation was first identified in 1906 in the egg-yolk protein, phosvitin, by Phoebus A. Levene.^9^ The enzymatic process of protein phosphorylation was first explained half a century later in 1954 when protein kinase activity was observed for the first time.^10^ Phosphorylation is an enzymatic reaction of protein kinases catalyzing the linkage between amino acid residues of the protein and phosphate groups in adenosine triphosphate (ATP). The reversibility arises from the actions of protein phosphatases which catalyze dephosphorylation by removing phosphate groups.

Protein kinases and phosphatases dynamically regulate the state of protein phosphorylation. According to the target phosphorylated amino acid residues, protein kinases can be characterized into three groups: serine protein kinases, threonine protein kinases, and tyrosine protein kinases. Protein phosphatases can also be clustered according to substrate specificity. Phosphorylation sites can be serine, threonine, tyrosine, cysteine, arginine, proline, aspartic acid, and histidine residues, but the most common target sites are serine (Ser), threonine (Thr), and tyrosine (Tyr). Protein phosphorylation receives the most attention and is the most intensively studied PTM. Phosphorylation usually occurs in the cytosol or nucleus and is considered a fundamental, universal and essential mechanism regulating protein activity and functions. Phosphorylation can rapidly control the function of proteins through two mechanisms.^21^ One is by allostery to activate enzyme activity (typically Ser/Thr, Tyr residues), as in the example of glycogen synthase kinase-3 via serine/threonine kinase. Another is by combining interaction domains to activate signal transduction (usually Tyr residues), as the instance of Src homology 2 domain-mediated autoinhibited conformation of the tyrosine kinase domain.

There are many research approaches for studying protein phosphorylation, such as kinase activity assays, phosphatase treatment, in vitro phosphorylation assays using γ-^32^P-ATP autoradiography, phosphor-specific antibody development, phospho-tag SDS-PAGE, ELISA (cell-based and enzyme-linked), immunohistochemistry (IHC), immunocytochemistry (ICC), flow cytometry, MS, and phosphoproteomics.^22^

Nearly 30% of human genome-coded proteins contain covalently bound phosphate. Reversible protein phosphorylation modulates almost every aspect of cellular processes relevant to replication, transcription, apoptosis, the immune response, environmental responsiveness, and cell metabolism.^14^ Abnormal phosphorylation has been recognized as the cause or consequence of diverse diseases, including tumors, CVDs, and metabolic disorders. Thus, drugs targeting protein kinases provide attractive therapeutic strategies against several diseases.^17–19^

### Acetylation

In 1964, protein acetylation was first identified on histone proteins isolated from calf thymus nuclei in vitro by V.G. Allfrey.^11^ In the 1990s, the first histone acetyltransferases (HATs) and histone deacetylases (HDACs) in mammals were discovered, which were recognized as transcriptional regulatory factors owing to the observation of p53 acetylation.^23^ Acetyltransferases add acetyl coenzyme A (acetyl-CoA)-derived acetyl groups (COCH_3_) to the ε-amino group in the lysine. Conversely, deacetylases catalyze the removal of the acetyl group from the side chains in lysine. Acetylated modifications include one irreversible type (Nα-acetylation) and two reversible types (Nε-acetylation and O-acetylation). These three types of acetylation can occur on diverse amino acids with different frequencies, and lysine Nε-acetylation is more commonly reported.

At present, there are three types of human deacetylases: HDAC1, HDAC2, HDAC3, and HDAC8 make up the class I HDACs; class II HDACs are grouped into class IIa (HDAC4, HDAC5, HDAC7, and HDAC9) and class IIb (HDAC6 and HDAC10); and class III HDACs are nicotinamide adenine dinucleotide (NAD^+^)-dependent sirtuins (SIRTs) containing SIRT1 to SIRT7. Acetylation is directly connected to acetyl-CoA levels. Mitochondrial and nonmitochondrial acetyl-CoA are independently engendered and can locally trigger acetylation. Recent research has shown that acetyl-CoA can regulate acetylation in a nonenzymatic manner.^24^

Several acetyl-lysine research tools include convenient acetylation detection kits, mass spectrometry with acetylated affinity beads, and immunofluorescence by specific acetyl-lysine antibodies.^25^

The dynamic balance between histone acetylation and deacetylation in cell nucleus changes chromatin structure and regulates gene expression. Acetylation stimulates chromatin de-condensation and promotes gene expression, whereas deacetylation induces suppression of gene expression. In the past decade, increasingly advanced proteomic information has vastly expanded the number of known acetylated nonhistone proteins. A considerable amount of nonhistone protein acetylation has been identified and found to be associated with vital cellular biology, including gene expression, DNA damage repair, cell cycle control, cell fate, protein folding, protein–protein interaction, autophagy, signal transduction, and cell metabolism.^23^ Therefore, disruption in acetylation is implicated in diverse conditions and diseases, including immune disorders, ageing, tumors, neurological conditions, metabolic disorders, and heart diseases.^26–28^

### Methylation

In 1959, protein methylation was initially reported in bacterial flagellar proteins by Richard Ambler and Maurice Rees.^29^ Methylation is the catalytic process of transferring methyl groups from active methyl compounds to amino acid residues. After decades of inactivity, due to advances in biology in the late 20th century, the investigation of protein methylation flourished leading to discoveries of extensive protein methylation and its potential functions. Methylation occurs mainly in nucleus and usually modifies nuclear proteins (for instance, histones). Protein methylation can occur on several amino acid residues. Methylation mainly modifies lysine and arginine residues.

There are expected to be more than 100 lysine methyltransferases (KMTs) in humans,^30^ such as SUV39H1 and enhancer of zeste homolog 2 (EZH2). Also, there are nine protein arginine methyltransferases (PRMTs) in mammals. PRMT1 primarily catalyzes asymmetric di-methylation, and PRMT5 is mainly responsible for symmetric demethylation.^31^ The arginine residue can be methylated up to two times and the lysine residue up to three times. One proton will be removed from the ɛ-amino group at each methylation, but these do not influence the total charge and will subsequently reduce the hydrogen-bonding capacity and increase the hydrophobicity. Methylation is an epigenetic regulatory process that mediates the transcriptional availability of DNA. Histone methylation occurs much slower than other histone PTMs (for example, phosphorylation and acetylation), indicating epigenetic stability.

Protein methylation can be investigated by methylation-site specific antibodies, mapping with mass spectrometry, protein or peptide immunoprecipitation (IP) with isotopic labeling or methionine labeling, and novel proteomic strategies to identify methylated substrates.^32^

Methylation is associated with various cell activities, including transcriptional regulation, epigenetic silencing, RNA processing and export, and signal transduction.^33^ Dysregulation in protein methylation results in multiple diseases, including cancer, mental abnormalities, metabolic disorders, and CVDs.^34–36^

### Ubiquitination

In 1975, ubiquitination was first discovered by Gideon Goldstein.^37^ Ubiquitin (Ub, 8.6 kDa) is a polypeptide of 76 amino acids that is highly conserved and widely expressed in eukaryotes. During ubiquitination, activated ubiquitin proteins are attached to Nε of the lysine residue of proteins and the subsequent modifications occur via a series of enzymes. Three steps are involved in ubiquitination: ubiquitin activation, conjugation and ligation. The ubiquitin-activating E1 enzyme activates ubiquitin in an ATP-dependent manner. Then, the ubiquitin-conjugating E2 enzyme is bound to the activated Ub-E1 complex, transferring ubiquitin from E1 to E2. At last, the ubiquitin E 3 ligase enzyme attaches to the lysine residues on the target protein and the C-terminal glycine on ubiquitin, leading to the subsequent modification and related effects.^38^

Most species contain only one E1 enzyme, ubiquitin-like modifier activating enzyme 1 (UBA1).^39^ E2 enzymes are a polygenic family, and their members vary in different species. Approximately 40 E2 enzymes have been discovered in humans, such as UBE2A, UBE2B, and UBE2C.^40^ Since E3 ligases link to the substrates and govern the peculiarity of ubiquitination, humans have over 600 E3 ligases. Based on different structures and functions, E3 ligases are classified into four types: HECT, RING-finger, U-box, and RBR types.^41^

Ubiquitination can modify all 20 amino acids, but lysine ubiquitination is the predominant form of ubiquitination. Ubiquitination is a well-recognized mechanism in endogenous protein degradation through the ubiquitin-proteasome system (UPS). This milestone discovery of UPS won the 2004 Nobel Prize in Chemistry. Functionally, the tag of one single ubiquitination drives the subsequent addition of ubiquitin and the growth of a polyubiquitin chain. The 26S proteasome will finally identify and degrade the polyubiquitinated protein, recycling the amino acids and ubiquitin.^38^ Once polyubiquitination occurs, the process becomes irreversible and the protein is destined for degradation. However, not all ubiquitination contributes to protein degradation.

Ubiquitin binding is reversible and can activate or inactivate proteins and regulate interactions among different proteins. The deubiquitinating enzyme (DUB) is a large family, including over 100 enzymes involved in removing a single ubiquitin and cleaving polyubiquitin chains. The regulation of ubiquitination depends on the conjugation of ubiquitin by ubiquitin ligases, in which deubiquitinating enzymes remove ubiquitin and counter the process.^42^

Current methodologies to study ubiquitination include activity-based probes (ABPs) targeting enzymes or the 26S proteasome, Ub tagging-based experiments, MS-based ubiquitination omics, ubiquitination site profiling with anti-diGly antibodies, ubiquitination-site specific antibodies or Ub COmbined FRActional DIagonal Chromatography (COFRADIC) and computational prediction.^43^

Ubiquitination is of great importance in the preservation and differentiation of stem cells and various cellular processes, such as cell proliferation, DNA repair, replication, transcription, protein degradation, autophagy and apoptosis, innate immunity and signal transduction.^44^ Dysfunction of ubiquitination is closely involved in various diseases, such as tumors, metabolic diseases, inflammatory diseases, and neurodegenerative diseases.^45–47^

### SUMOylation

Small ubiquitin-related modifier (SUMO)-related PTMs were primarily found in yeast with the discovery of the yeast orthologue SMT3 in a genetic inhibition screening for the Mif2 protein by Meluh and Koshland in 1995.^48^ The SUMO protein is a 10-kDa polypeptide that links to the ɛ-amino groups of lysine residues via isopeptide bonds, and this is termed SUMOylation. SUMO proteins carry similarity (less than 20%) with ubiquitin in amino acid sequence. The N-terminus of all SUMO proteins share a formless sequence of 10–25 amino acids that is absent from ubiquitinated proteins. The SUMO family varies in diverse species, and there are two isoforms in yeasts, eight in plants, three in mammals, and four in humans.^49^

SUMOylation is a highly dynamic enzymatic cascade requiring SUMO-activating E1 enzyme (SAE1/UBA2), SUMO-conjugating E2 enzyme (UBC9), and SUMO E3 ligase, similar to but distinct from ubiquitination. In contrast to hundreds of identified ubiquitin E3 ligases, only a tiny number of SUMO E3s have been recognized, including nuclear pore protein RanBP2, tripartite motif-containing (TRIM) families, protein inhibitor of activated STAT (PIAS) and polycomb group protein Pc2.^50^ SUMOylation can occur in cell nuclei, cytoplasm, plasma membrane, ER, and mitochondria and is accordingly widespread in eukaryotic organisms. The acceptor lysine motif in target proteins is commonly found as ΨKxE/E (where Ψ refers to the hydrophobic residue such as valine, isoleucine, or phenylalanine; K is the SUMO-conjugated lysine; x represents any amino acid; D or E is an acidic residue),^51^ although increasingly non-consensus acceptor sites are being identified. SUMOylated modification alters protein localization, activity, and stability by covering or appending interaction surfaces. Some specific short SUMO-interaction motifs (SIMs) have been recognized noncovalently in target proteins, SUMO enzymes, and downstream effectors.^52^

SUMOylation is a reversible modification. Sentrin/SUMO-specific protease (SENP) mediates deSUMOylation, where SUMO is deconjugated to the target amino acids. The human SENP proteins include seven members: SENP1-3 and SENP5-8, but SENP8 shows no action on SUMO and has specificity for the NEDD8 protein.^53^

Methodologies to investigate SUMOylation include purifying SUMOylated protein, SUMO-fluorescent conjugation analysis, surface plasmon resonance-based SUMO-SIM interactions, PLA, biotin or histidine-tag assay, polymeric protein scaffold-based assay, and MS-based proteomics.^54^

SUMOylation contributes to many biological processes, including chromatin organization, DNA repair, transcription control, accumulation of macromolecules, cell cycle progression, trafficking, gene expression,^55^ and cell signaling pathways. There are many reports of abnormal SUMOylation in diseases, including tumors, Alzheimer’s disease, Parkinson’s disease, CVD, and metabolic disorders.^56,57^

### Neddylation

Neddylation is a PTM akin to ubiquitination and was first reported in 1997 by Tetsu Kamitani.^58^ Neddylation attaches the ubiquitin-like protein neuronal precursor cell-expressed developmentally downregulated protein 8 (NEDD8) to the lysine residues of proteins. The ubiquitin superfamily includes 17 members, including ubiquitin, SUMO, and NEDD8. NEDD8, an 81-amino acid polypeptide, has remarkable similarity with ubiquitin, which shares 80% homogeneity and 60% identity with ubiquitin.^58^ NEDD8 is a conserved and predominantly nuclear protein.

Analogous to ubiquitination, protein neddylation is a highly dynamic enzymatic cascade that requires NEDD8-E1-activating enzyme (NAE), NEDD8-E2-conjugating enzyme (UBE_2_F, UBE_2_M), and specific NEDD8-E3 ligases. The most distinctive substrate of NEDDylated modification is the cullin subunit of Cullin-RING ligase (CRL). NEDD8 attaches to the lysine group at the C-terminus of cullins, spacing the CRL negative factor CAND1 and promoting CRL assembly for activation.^59^ All presently known NEDD8 E3 ligases could serve as E3 ubiquitin enzymes, and the majority of E3 NEDD8 ligases have the RING domain. CRLs are the principal family belonging to multiunit E3 ligases, regulating the breakdown of ~20% of proteasome-controlled proteins. The most common and studied NEDD8 E3 enzymes include RXB1 (CRL1, CRL2, CRL3, CRL4 complexes) and the homolog RXB2 (CRL5).^60^ De-neddylation enzymes detach NEDD8 from target proteins, including NEDD8-specific protease 1 (NEDP1), CNS5-derived eight-subunit COP9 signalosome (CSN), spinal-cerebral-ataxia related protein 3, and ubiquitin-specific peptidase 21 (USP21).^61^

The neddylation detection assay includes coincubation experiments, cellular thermal shift assays, isothermal titration calorimetry (ITC), biolayer interferometry (BLI), and high-throughput screening (HTS) combined with molecular docking, facilitating the confirmation of potential targets and the development of novel regulators.^62^

Overall, protein neddylation affects protein localization, stability and function. Neddylation participates in diverse cell processes, including DNA damage, cell apoptosis, immune regulation, and signaling pathways.^59,60^ Abnormal neddylation is involved in various diseases, including tumors,^59^ neurodegenerative disease, metabolic diseases and CVD.^63,64^

### Glycosylation

In 1981, N-Glycosylation was the first type of glycosylated modification studied by E Bause and G Legler.^65^ Glycosylation is thought to be the most abundant and complex PTM, and accordingly, it vastly expands the diversity of the proteome. Glycosylation describes a series of reversible enzymatic processes of the glycoconjugates (composed of glycans or carbohydrate chains) covalently linked to the protein or lipid via glycosyltransferase or glycosidase. Glycoconjugates differ in their glycan composition, linkage, structure, and length, thus facilitating diversity. Glycosylation modifies approximately one-half of the plasma proteins, while membranes and secreted proteins are commonly glycosylated. Glycosylation can occur in cytosol, sarcolemma membrane, endoplasmic reticulum and the Golgi complex. According to the linked residues, glycopeptide bonds, and attached oligosaccharides, glycosylation can be categorized as N-glycosylation (linked to asparagine residues), O-glycosylation (attached to serines and threonines), C-glycosylation, S-glycosylation, glypiation, and phosphoglycosylation.^66^ N-glycosylation and O-glycosylation are two key types of protein glycosylation.

N-glycosylation represents the most common glycosylation, attaching N-acetylglucosamine (GlcNAc) to the conserved motif Asn-X-Ser/Thr by a β1-glycosidic linkage. N-glycosylation includes three processes: N-glycan synthesis, transfer, and modification. N-glycanidine biosynthesis and transfer only occur in ER, but the modification can occur in both ER and Golgi complex. Glycosyltransferases such as ALG7 and ALG13/14 produce N glycans. Oligosaccharyltransferase (OST) transfers the oligosaccharide chain to asparagine. Glycosyltransferases and glycosidases-mediated shearing and processing culminate in the formation of complex, heterogeneous N-glycan chains.^65^ N-glycanase (PNGase) specifically hydrolyzes asparagine (Asn)-linked oligosaccharides, mediating deglycosylation.^67^ N-glycosylation usually takes place cotranslationally, in which glycoconjugates are bound to the target protein during translation and transport into the endoplasmic reticulum.^65^ N-glycosylation thus regulates the functions of a majority of glycoproteins. The approaches to identify site-specific glycosylation are specific enzymatic proteolysis, fractionation of glycopeptides (liquid or affinity chromatography), and glycopeptide analysis by MS. Specifically, different specific endoglycosidases combined with isotope dimethyl labeling could quantitatively investigate the N-glycoproteome.^68^

O-glycosylation links GlcNAc and N-acetylgalactosamine (GalNAc) to serines or threonines from the oxygen atom in hydroxyl groups. O-glycosylation often occurs posttranslationally in the Golgi apparatus. O-glycans are formed by conserved O-GlcNAc transferase and reversibly broken down by the highly conserved O-glcNAcase. O-linked glycosylation is vital in the synthesis of mucins.^65^ No generic enzymes can directly deglycosylate O-glycans, making O-glycan release difficult and making analysis challenging. O-glycosidase fails to completely cleave complex O-glycans, and chemical methods (β-elimination, peeling reaction, and end-capping strategies) must be applied for intact glycan release.^69^

Approaches to study protein glycosylation include glycogene-chip; glycosyltransferase and glycosidase activity detection with radiochemistry, chromatography, spectrophotometry, and bioorthogonal chemical reporters; glycan analysis by lectin binding assays, chromatography, mass spectrometry, and novel fragmenting technologies (electron capture and transfer dissociation); and glycopeptide enrichment.^70^

Glycosylation is crucial in regulating cellular processes, including protein folding, degradation, secretion, molecular trafficking and clearance, cell adhesion, cell-cell interactions, signal transduction, receptor activation, and endocytosis.^66,71^ Dysregulation in glycosylation affects the development of human diseases, including tumors, atherosclerosis, diabetes, liver cirrhosis, and Alzheimer’s disease.^72–74^

### Palmitoylation

Palmitoylation was first reported by J Folch in 1951.^75^ Myristoylation, and prenylation are the three major types of lipidation, describing the covalent binding of lipids to proteins, palmitoylation. These PTMs occur by thioester linkages of various fatty acids, including palmitate, myristic acid, stearic acid, octanoic acid, and cholesterol. Palmitate, a sixteen-carbon saturated fatty acid, can attach to cysteine residues by a thioester bond. This is considered a palmitoylated modification, which can increase the hydrophobicity of proteins and promote protein-lipid bilayer interactions.^76^ The labile and reversible thioester linkage of palmitate dynamically changes protein-palmitoylation levels in response to physiological stimulation, providing a critical potentiality to regulate cell development and signaling. The initial discovery of palmitoyltransferases was in yeast by Bartels in 1999.^77^ Palmitoyl-CoA is linked to target proteins by palmitoyltransferases and detached by thioesterases.

Chemical approaches to investigate protein palmitoylation include radio-labeled fatty acid reporters with autoradiographic detection or biorthogonal fatty acid reporters with bioorthogonal reactions.^78^ Protein palmitoylation plays critical roles in protein sorting, protein functions, protein-protein interactions, apoptosis, neuronal development, and signal transduction.^76^ Several pieces of evidence have indicated the crucial roles of palmitoylation in neurological diseases, cancers, and metabolic disorders.^79–81^

### Myristoylation

Myristoylation was first reported in the bovine brain by Alastair Aitken in 1982.^82^ During myristoylation, the fourteen-carbon saturated fatty acid, myristic acid, attaches to the N-terminal of glycine by a covalent bond after cleavage of the initiator methionine. Myristoylation usually takes place co-translationally and irreversibly on cytoplasmic eukaryotic proteins. However, posttranslational myristoylation also occurs in cell apoptosis.^83^ Myristoylated proteins are frequently transported to the membrane according to the orientation of the myristoyl group, which usually promotes membrane binding. N-myristoyl transferases (NMTs) mediate attachment using myristoyl-coenzyme A as a substrate. The existence of NMT has been identified in most eukaryotes, but not in prokaryotes.^84^ Lower eukaryotes only express a single type of NMT, while mammals have two isozymes, NMT1 and NMT2. A few reports indicate the existence of demyristoylation, but the evidence is scarce, and the mechanism is still less understood.^85^

Myristoylation is vital in protein stability, protein localization, protein structure maturation, extracellular communication, immune response, cell metabolism, and signal transduction.^86^ Dysregulation in protein myristoylation has been reported in the development of cancer, neurological diseases,^87^ viral and bacterial infections, and metabolic disorders.^84,88,89^

### Prenylation

Prenylation was first discovered in yeast by KamiIya in 1978.^75^ Prenylation is an irreversible modification ubiquitously occurring in all eukaryotic cells. Prenylation describes the covalent addition of isoprenoids to the carboxyl-terminal or cysteine residues.

Prenylation includes two major forms^90^: farnesylation (linkage of a fifteen-carbon farnesyl pyrophosphate) and geranylation (attachment of a twenty-carbon geranylgeranyl pyrophosphate). Three isoprenyl transferases catalyze these modifications. Farnesyltransferase (FTase) modulates the combination of a single geranylgeranyl group, whereas geranylgeranyltransferase type-1 (GGTase-I) adds a single geranylgeranyl group. The common sequence in C-terminal of the target cysteine is the “CaaX” box, in which “C” represents a cysteine, “a” represents the aliphatic amino acid, “X” could be any amino acid responsible for the attached isoprenoid.^91^ GGTase-II catalyzes dual geranylgeranyl groups attaching to double cysteine residues in motifs like “CCXX” or “CXC“.^91^ Prenylated proteins experience farnesylation, proteolytic removal of the “aaX” sequences, carboxymethylation and finally become oriented and the plasma membrane. Well-known prenylated proteins include Ras superfamily proteins, Ras-related small guanosine triphosphate-binding proteins (G proteins), and trimeric G proteins.^91^

Several tools to study protein prenylation include chemical proteomic analysis with alkyne-containing probes.^92^ Prenylation is the first necessary process in membrane targeting and binding and involves subsequent protein-protein interactions, protein trafficking, cell movement, cell growth, differentiation, and proliferation.^93^ Disruption in prenylated modification is observed in the pathogenesis of tumors, neurodegenerative diseases, bone diseases and cardiometabolic diseases.^94,95^

### Cholesterylation

Cholesterol modification (cholesterylation) was first found in hedgehog (Hh) proteins by Porter in 1996.^96^ For the next 20 years, hedgehog was considered the only cholesterylated protein until another cholesterol-modified protein, smoothened (SMO), was reported by Song and colleagues in 2017.^97^ Cholesterol modifies SMO at the Asp95 residue, which is necessary for the Hh protein pathway and embryonic development. Song and colleagues subsequently revealed that cholesterylation of SMO is an autocholesterylation process promoted by calcium.^98^ Biorthogonal labeling can be applied to analyse and identify novel cholesteryled proteins.^99^ Several approaches to detecting protein cholesterylation exist, such as biorthogonal labeling with azido-conjugated cholesterol analogs and alkynyl sterol probes.^99^

### Glutathionylation

The primary report of protein glutathionylation dated back to 1985 by Ziegler.^100^ However, the comprehensive understanding of protein glutathionylation associating reactive oxygen/nitrogen species (ROS/RNS) did not emerge until the last decade. S-glutathionylation describes the bond formed between glutathione (GSH) and the thiol group (-SH) of cysteines via a mixed disulfide linkage. This reversibly adds a negative charge and a tripeptide to alter the protein structure, charge, and functions. Glutathione S-transferase (GST) can catalyze S-glutathionylation, or S-glutathionylation can occur spontaneously.^101^ Thioredoxin, glutaredoxin (Grx), and sulfiredoxin can regulate the reversal of S-glutathionylation.^101^ The rate of GSH and glutathione disulfide (GSSG) mainly serves as the sensor of the intracellular redox state and can be reduced by oxidative or nitrosative stress under physiological or pathological conditions.^102^ Many proteins undergo S-glutathionylation, covering the cytoskeleton, cell metabolism, kinase, calcium pathway, antioxidant homeostasis, protein folding, and signal transduction.^103^

Given the potential significance of glutathionylation, numerous developing techniques could identify protein glutathionylation. The basis for the current proteomic investigation is labeling glutathione with ^35^S radiolabelling and biotinylation.^104^ There is also novel computational prediction by the position-specific matrix.^105^

Due to the abundance and significance of glutathione, the S-glutathionylation cycle plays vital roles in cell fate, cell proliferation, differentiation, apoptosis, antioxidant homeostasis, cell metabolism, immune response, inflammation and signaling pathways.^102,106^ An imbalance in S-glutathionylation results in a series of diseases, such as infection, tumors, neurodegenerative diseases,^107^ CVDs, and metabolic diseases.^106,108–110^

### S-nitrosylation

Although S-nitrosylation was primarily investigated by Shigeru Oae and Koichi Shinhama in 1983,^111^ it took another 30 years before S-nitrosylation was recognized as nitric oxide (NO)-dependent PTM.^112^ S-nitrosylation is the covalent incorporation between the nitrosyl moiety of NO and target molecules. S-nitrosylation occurs at the cysteine thiol group, producing protein S-nitrosothiols (SNOs). If nitrosylation occurs at a transition metal, this is termed metal nitrosylation. Awarded as “the Molecule of Year” in 1992, the dissolved gas, NO, is of great significance in biology and has been associated with extensive research and numerous awards, including the Nobel Prize in 1998. In the classical NO pathway, NO induces the generation of cyclic guanosine monophosphate (cGMP) and activation of cGMP-dependent protein kinase (PKG) signaling through attachment to guanylyl cyclase (GC).^113^ In the nonclassical NO pathway, S-nitrosylation mediates the major mechanism.^114^ S-nitrosylation can consume NO to prevent the reaction between NO and ROS and guard cysteine thiols against ROS-induced oxidation at a low ROS level. Most proteins act as substrates for S-nitrosylation. Some enzymes are involved in S-nitrosylation and de-nitrosylation, but the mechanism of dynamic regulation has remained less explored.^114^ There are precise space and time mechanisms regulating S-nitrosylation and denitrosylation. For instance, the location of the target cysteine, the specific motif sequence “I/LXC-X2-D/E” in cysteine, a highly hydrophobic region, and the suitable environment jointly confirm the specificity of S-nitrosylation.^115^

Tools to detect protein S-nitrosylation include biotin-switch-based mass spectrometry, immunochemistry with specific anti-SNO antibodies, chemical strategies by gold nanoparticles, organomercury resin capture, organophosphine-related biotin labeling, and labeling based on one-step disulfide production.^116^

S-nitrosylation regulates various cellular mechanisms, including transcription, protein stability, localization, trafficking and interaction, cell growth and apoptosis, cell metabolism, signaling pathways, and further protein modification phosphorylation, acetylation, and ubiquitination.^114^ An imbalance in S-nitrosylation is implicated in the occurrence of various human diseases, such as cancer,^117^ neurodegenerative diseases, respiratory diseases, cardiovascular diseases, and metabolic disorders.^118–120^

### Sulfhydration

First identified in mouse liver lysates by protein analysis in 2009, sulfhydration describes the PTM involving the alteration of the thiol group (-SH) in reactive cysteine residues to a persulfide (-SSH) group, leading to the enhanced reactivity of the cysteine residue, akin to nitrosylation.^121^ Hydrogen sulfide (H_2_S) functions as an imperative gasotransmitter/signaling molecule and is crucial in physiological processes analogous to NO. Mechanically, H_2_S facilitates its role through protein sulfhydration. H_2_S physiologically modifies nearly 10%-25% of hepatic proteins by S-sulfhydration, including tubulin, actin, and glyceraldehyde-3-phosphate dehydrogenase.^122^ Sulfhydration regulates protein function and mostly depends on the structure and spatial arrangement of sulfhydrated residues. Sulfhydration protects cysteine residues against oxidative damage, leading to remission of permanent injury and amelioration of protein function. Sulfhydration is similar to nitrosylation, by which both are reversible and occur on the cysteine residue, but they are differentiated from each other. Sulfhydration is more common than nitrosylation, as 25–50% sulfhydrated proteins are detected in murine liver.^122^ Sulfhydration generally activates enzyme activity, whereas nitrosylation usually suppresses protein function.^123^

Approaches to exploring protein sulfhydration include biotin-switch analysis, cysteinyl labeling examination, tag-switch assessment, protein persulfide detection, and mass spectrometry analysis.^124^ Sulfhydration orchestrates various processes, including inflammation, endoplasmic reticulum stress, signal transduction, blood pressure, and vascular tension.^125^ Disruption in sulfhydration mediates abundant diseases, such as Alzheimer’s disease, Parkinson’s disease, CVDs and metabolic disorders.^126–129^

### Citrullination

The citrullinated modification was first reported in detail by Rogers in 1958.^130^ Citrullination, also known as deimination, is an irreversible chemical process converting arginine to citrulline, during which positively charged arginine is chemically hydrolyzed to uncharged citrulline and neutral urea. Citrulline is a nongenetically coded type of amino acid, and citrullination only takes place posttranslationally. This charge conversation will affect protein structure, charge, hydrogen bond generation, protein-protein interactions, and even protein denaturation.

Citrullinated modifications can involve numerous cellular proteins, including those in the nucleus, cytoplasm, mitochondria, and cell membrane. This modification is catalyzed by peptidylarginine deiminases (PADs), enzymes that appear to be activated by high calcium concentrations. The catalytic process of PAD enzymes was initially described in 1977.^131^ Five calcium-dependent isozymes (PAD1, PAD2, PAD3, PAD4, PAD6) are identified in humans, which share a 50% similar sequence.^132^ Diverse PAD enzymes are distributed widely in cells and tissues. Especially, PAD4 is found only in the nucleus and is essential in histone deamination, whereas the other four isozymes are located in the cytoplasm.^133^

Current strategies to study protein citrullination include COLDER assessment, immunochemistry with specific anti-citrullination antibodies, mass spectrometry, chemical derivatization targeting citrulline, and phenylglyoxal probe-based assays.^134^

The activity and balance of PADs play a role in citrullination and cellular processes, including protein stability and structure, protein-protein interactions, cell apoptosis, and cell death.^135^ Abnormalities in protein citrullination lead to multiple sclerosis, cancer, rheumatoid arthritis, systemic lupus erythematosus,^136^ Alzheimer’s disease and metabolic disorders.^137–139^

### ADP ribosylation

Protein adenosine diphosphate (ADP)-ribosylation was primarily defined by Chambon in the early 1960s.^140^ ADP-ribosylation transfers ADP-ribose (ADPr) from NAD^+^ to the target protein and releases nicotinamide (Nam). This modification includes mono-ADP-ribosylation (MARylation) and poly-ADP-ribosylation (PARylation). PARylation possesses specific characteristics due to the synthesis and nature of ADP-ribose chains. ADP-ribosylation takes place in the side chains with sulfur, nitrogen, or nucleophilic oxygen, leading to S-, N-, or O-glycosidic attachment to the ribose. ADPr carries an adenine ring, two ribose moieties, and two negative charges, enabling hydrophobic interactions and hydrogen linkage. In this manner, ADP ribosylation offers diverse modalities to change protein structure and functions. ADP-ribosylation is a reversible event where ADP-ribosyltransferases (“writers”) covalently add ADPr, whereas ADP-ribosylglycohydrolases (“erasers”) remove ADPr. “Readers” describes the interaction with ADPr.^141^

Based on structural homology, the ADP-ribosyltransferase (ART) superfamily is characterized as ART diphtheria toxin like (ARTD) and ART cholera toxin like (ARTC). ARTDs include the majority of poly (ADP-ribose) polymerases (PARPs) and tankyrases (TNKS). The PARP family includes two tankyrases: tankyrase 1 (TNKS1, also termed PARP5A or ARTD5) and tankyrase 2 (TANK2, also named PARP5B or ARTD6).^142^ Viruses, prokaryotes, and eukaryotes all share conserved ART domains.

Hydrolases remove ADPr, which varies in structure and function, including MacroD1, MacroD2, terminal ADP-ribose protein glycohydrolase 1 (TARG1), poly-ADP-ribose glycohydrolase (PARG), and ADP-ribosyl-acceptor hydrolases (ARHs).^143^

Approaches to exploring protein ADP-ribosylation include chemical tools (such as α-ribosyl amino acids, (pyro)phosphate, ADP-ribosylated peptides, and analogues, polyADPr chains), macroGreen, fluorescence-related assessment, and molecular toolbox.^144^

ADP ribosylation regulates major cellular processes, including DNA repair, cell growth and differentiation, cell metabolism, stress responses, and immunity.^145^ Dysregulation of ADP-ribosylation can lead to human diseases, including cancer, ischaemia-reperfusion-like tissue injury, heart disease, neurological disorders,^146^ and metabolic disorders.^147–149^

### Carbonylation

The introduction of carbonyl groups into protein was first reported during studies of glutamine synthesis in 1983.^150^ Protein carbonylation (PCO) is a type of protein oxidation that produces carbonyl groups such as reactive ketones, aldehydes, or lactam, facilitated by reactive oxygen species (ROS). PCO is a nonenzymatic and deleterious PTM, as the introduction of carbonyl groups into target proteins marks oxidative damage and destroys protein structure and function.

PCO is divided into four groups: the breakage of protein and polypeptide main chains; the oxidation of amino acid side chains; lipid peroxide addition to active site; and glycation oxidation products.^151^ The technology for determining carbonyl content is based on the formation of 2,4-dinitrophenylhydrazone. Spectrophotometry and chromatography can measure the total protein carbonyl content.

Valuable tools to study protein carbonylation include measuring the carbonyl level by the Levine spectrophotometric assay based on the chromogenic reaction with 2,4-dinitrophenylhydrazine (DNPH), ELISA, western blot with anti-DNPH antibodies, and in-gel detection assay by fluorescence.^152^

PCO leads to irreversible damage. PCO acts as the hallmark of oxidative stress and is closely implicated in regulating protein function and cell senescence.^153^ Dysregulation of PCO is seen in skeletal muscle dysfunction, Alzheimer’s disease, and metabolic disorders.^154–156^ The physiological roles of PCO in oxidant signaling indicate that drugs controlling carbonyl content might possess clinical value.

### Other oxidative modifications

Protein oxidative modifications are an appreciable group of protein PTMs, which are induced by ROS, reactive sulfur species (RSS) or reactive nitrogen species (RNS). Cysteine is the molecular basis for thiol-mediated redox control. Common oxidative reversible alterations of cysteine thiols include S-nitrosylation (-SNO), S-sulfenylation (-SOH), glutathionylation (-SSG), disulfide formation (-SSR) and S-sulfhydration (-SSH). Furthermore, the biologically stable modifications mainly cover S-sulfinylation (-SO_2_H) and S-sulfonylation (-SO_3_H). We have described some oxidative modifications such as S-nitrosylation, glutathionylation, and sulfhydration, above, so here we will give a brief introduction to other protein oxidative modifications.

#### S-sulfenylation (-SOH)

The study of S-sulfenylation commenced in 1976.^157^ S-Sulfenylation is a process where hydrogen peroxide (H_2_O_2_) converts oxidized specific cysteine thiols to sulfenic acid (-SOH). Most interaction between cysteine thiol groups and H_2_O_2_ is slow, which depends on the protein microenvironment, pH, pKa(-SH), and the presence of bulky groups around the thiol groups.^158^S-Sulfenylation serves as an intermediate redox sensor leading toward other oxidative modifications, including S-glutathionylation and disulfide formation. This reversible modification can control molecular thiol switches to modulate protein stability, activity, interactions, conformational alteration, and cellular location.^159^ Approaches to identifying SOH are usually indirect, including protein engineering techniques, chemical labeling, single-molecule force-clamp spectroscopy, and mass spectrometry.^160^ Aberrant sulfenylation contributes to numerous diseases, including tumors, senility, CVDs, obesity, diabetes and neurodegenerative diseases.^158^

#### S-sulfinylation (-SO_2_H)

Protein thiol oxidation yields SOH, which is oxidized further to form S-sulfinic acid (-SO_2_H). This reversible process is sulfinylation (-SO_2_H). Hyperoxidation of SOH to SO_2_H relies on SOH ionization and the nucleophilic assault of H_2_O_2_. The generation of SO_2_H is commonly related to oxidative stress. Sulfiredoxin decreases S-sulfinic acid (-SO_2_H) back to the thiol in an ATP-dependent manner.^161^ The reversibility of SO_2_H indicates that sulfinic acid formation plays a role in redox regulation, which enables H_2_O_2_ signals to exert regulatory effects. It was reported that 5% of the hepatic cysteines in the rat are present as SO_2_H.^162^ S-sulfinylation is an elusive modification, and the identification depends on chemical probes, electrophilic probes and mass spectrum.^163^

#### S-sulfonylation (-SO_3_H)

Protein thiol oxidation can yield SOH, further generate SO_2_H, and finally, produce S-sulfonic acid (-SO_3_H). This is the process of sulfonylation (-SO_3_H), which irreversibly deactivates proteins.^164^ GSH could bind to SOH and form the protein glutathione mixed disulfide (PSSG), fundamentally avoiding the further oxidation of lipoate and blocking the irreversible alteration of SOH to SO3H.^165^

#### Disulfide formation (-SSR)

Disulfide bonds were found in coagulated egg albumin in 1907 by Heffter and in 1911 by Arnold.^166^ This two-electron reaction requires an electron acceptor or oxidant. Disulfide-bond formation in cellular proteins occurs as a series of catalyzed processes that are essential to the function of membrane and secreted proteins. Disulfide bonds primarily take place in the periplasmic space for prokaryotic cells or ER for eukaryotic cells.^167^ Disulfide bonds appear either intramolecularly (on two cysteines in the same polypeptide chain) or intermolecularly (between two proteins). The mixed disulfide is the disulfide bond linking the cysteine and a thiol-containing redox reagent (like dithiothreitol or glutathione). Intramolecular disulfide bonds attribute to stabilizing the tertiary structures of proteins, while intermolecular disulfide bonds contribute to stabilizing the quarternary structure.

The reversibility of disulfide formation allows its regulatory effects on protein folding, assembly, structure, stability, and function. Two redox-sensitive cysteines and the related disulfide bonds can serve as redox-sensitive switches. Redox-sensing switches can be present in abundant proteins, including enzymes, receptor proteins, sensor proteins, and transcriptional factors.^168^ Disulfide bonds can be detected by nuclear magnetic resonance, X-ray crystallography, LC-Fourier transform tandem mass spectrometry (FT MS/MS), and MassMatrix MS/MS search engine.^169^

Intramolecular disulfide bonds have been reported to be involved in G protein signaling,^170^ antioxidant enzyme Prdx1, thiol peroxidase, cholesterol metabolism, multiple myeloma, Alzheimer’s disease^171^ and amyotrophic lateral sclerosis. Intermolecular disulfide bonds play a role in innate immunity, prion diseases, Alzheimer’s disease, and vascular diseases. Mixed disulfide bonds are involved in immune response and celiac disease.^172^

### Novel types of PTMs

Recently some novel PTMs have emerged. Here, we will briefly introduce these novel types of PTMs.

#### Succinylation

Succinylation is a unique, recently discovered, and less understood PTM. Succinylation was first identified in *Escherichia coli* in the context of the catalytic activation of homoserine by Ran Rosen in 2004.^173^ Protein succinylation is conserved in prokaryotes and eukaryotes, describing the process of the covalent attachment of the succinyl group derived from succinyl-CoA to the lysine residue. Since the succinyl group is large (100 Da), the succinylated PTM results in a significant mass change and alters the physiological charge from −1 to +1; accordingly, succinylation possesses a significant effect on protein structure and function compared to acetylation (40 Da) or methylation (14 Da).^174^ Succinylated modification can occur in the nuclei, cytoplasm, and mitochondria.

As the principal regulator of succinylation, succinyl-CoA is positively associated with nonenzymatic succinylation. In addition, the α-ketoglutarate dehydrogenase complex (α-KGDHC) controls succinylation either by direct enzymatic succinylation or by regulating the levels of succinyl-CoA.^175^ Carnitine palmitoyltransferase 1A (CPT1A), another lysine succinyltransferase in mammalian cells, promotes succinylation without changing succinyl-CoA levels.^176^ NAD^+^-dependent SIRT5 is a desuccinylase that functions in all cell compartments.^175^

Because of the low content and a broad dynamic range of succinylated proteins in cells, enrichment of succinylated peptides is required to increase their abundance before mass spectrometroscopic analysis, and then quantitative analysis of the enriched succinylated peptide samples is performed using traditional quantitative proteomic analysis tools. Moreover, several computational predictions based on websites and deep learning methods are becoming increasingly common.^177^

Succinyl-CoA serves as a crucial metabolic intermediate in tricarboxylic acid (TCA) cycle and a vital donor of succinylation at the same time, allowing succinylation to govern cell metabolism and signal transduction.^178^ Accumulating evidence indicates that protein succinylation is involved in transcription modification, immune response, and cell metabolism covering the TCA cycle, urea cycle and fatty acid metabolism with altered metabolism.^179–181^ Current data has shown that dysfunction of succinylation leads to many diseases, such as inflammatory diseases, tuberculosis, ischaemia-reperfusion-like tissue injury, and metabolic diseases.^182–184^

#### Crotonylation

Lysine crotonylation (Kcr) was first reported in male germinal cells and human somatic cells by Zhao and colleagues in 2011 and was recognized as an epigenetic research highlight of 2011 by the journal, *Cell*, in the same year.^185^ The crotonyl group has an exclusive C-C π‐bond, leading to a rigid configuration. Kcr is usually found on histones in transcriptionally active chromatin regions and is closely associated with reproductive regulation. Kcr can be regulated reversibly by crotonyltransferases and decrotonylases. Kcr can be controlled by a nonenzymatic mechanism, and crotonyl-CoA profusion is one modulating factor of Kcr.^186^

Kcr “writer” refers to enzymes that catalyze histone crotonylation, which is influenced by intracellular crotonyl-CoA. Both genetic and environmental factors can regulate Kcr. Three main HAT families exhibit extended histone crotonyltransferase (HCT) activities, including p300/CREB-binding protein (p300/CBP), MYST, and GNAT (Gcn5-related N-acetyltransferase).^187^

The group of Li and colleagues identified a novel crotonylation “reader” (the AF9 YEATS structural domain) in 2016.^188^ AF9 YEATS structural domain can directly link Kcr to transcriptional activity. Double plant homeodomain finger proteins, YEATS domain proteins, and bromodomain proteins have been identified as three major families of readers.

In 2017, a crotonylation “eraser” appeared with the discovery that HDACs, but not the sirtuin family, are the main histone decarboxylases.^186^ Histone crotonylation is dynamically regulated in mammalian cells in the same way as histone acetylation. CDYL can negatively regulate histone crotonylation by serving as a crotonyl-CoA hydratase to change crotonyl-CoA to β-hydroxybutyric-CoA. Moreover, crotonylation can also occur on nonhistones.^189^

Current tools for the study of crotonylation are water-soluble phosphine warhead-based probes and bioinformatics detection by deep learning.^190^

Lysine crotonylation is associated with numerous cellular processes including DNA damage and repair, differentiation of stem cells, spermatogenesis, and inflammation.^186,187,191^ Dysregulation of lysine crotonylation is involved in diverse human diseases, including tumors, neuropsychiatric disease, and cardiovascular diseases.^192,193^

#### Beta-hydroxybutyryration

Beta-hydroxybutyryration (Kbhb) is a novel acylation modification mediated by β-hydroxybutyrate (β-OHB), first proposed by Zhao and colleagues in 2016.^194^ The acyltransferase p300 catalyzes the attachment of β-OHB to lysine, whereas HDAC1 and HDAC2 reversibly eliminate Kbhb. By this, β-OHB has been simply considered a functional carrier transferring energy from the liver to peripheral tissues upon starvation stress. β-OHB is also an important signaling and epigenetic regulatory molecule that regulates all aspects of life functions in vivo. β-OHB can mediate lysine Kbhb on histones of several hunger-associated genes, assisting the body to quickly adapt to metabolic shifts caused by energy shortage.^195^ Subsequent studies have revealed that in addition to histones, β-OHB can modify nonhistone proteins and participate in regulating diseases such as cancer and cardiometabolic diseases.^196–199^

Many key metabolic enzymes have multiple Kbhb sites, such as the urea cycle rate-limiting enzyme CPS1, the ketogenic rate-limiting enzyme HMGCS2, and S-adenosyl-L-homocysteine hydrolase (AHCY) in the methionine cycle.^200^ β-OHB can regulate cellular functions by directly affecting intracellular acetyl-CoA, succinyl-CoA, and NAD^+^ levels or by inhibiting histone deacetylase (HDAC) activity, thereby altering protein acetylation, succinylation, and other downstream molecular events. Kbhb-altered proteins are broadly distributed in the cytoplasm, mitochondria, and nucleus, suggesting a broad impact of β-hydroxybutyrylation modifications on cellular functions.^194,201^ Immunochemistry with crotonylation-modified pan antibodies, site-specific antibodies, and mass spectrometric techniques are the major detection methods for Kbhb.

#### Lactylation

Zhao and colleagues identified the novel histone acylation code, lactylation, in 2019.^20^ The researchers determined that lactylation occurred on histone lysine in human and mouse cells, which could trigger gene transcription directly. Enzymatic lysine lactylation transfers the lactyl group from lactyl coenzyme A (lactyl-CoA) to lysine, catalyzed by lysine acetyltransferase (KAT) enzymatic P300 and regulated by lactyl-CoA.^20^ Nonenzymatic lysine lactylation is derived from methylglyoxal, a glycolytic by-product, producing lactoylglutathione (LGSH).^202^ Lactylation results from lactic acid produced by cellular glucose metabolism and is regulated by lactic acid, glycolysis, and mitochondrial oxidative metabolism. Histone lactylation is abundant in late M1 macrophage polarization and shows diverse temporal dynamics compared with histone acetylation.^20^ Immunochemistry with specific antibodies and mass spectrometric techniques are the primary detection methods for lactylation. Lactylation is involved in gene expression, cell differentiation,^203^ and inflammation. Aberrant lactylation has been proposed in cancer, fibrosis, and cardiometabolic diseases.^204–207^

## PTMs in metabolic diseases

### PTMs in diabetes

**Fig. 3:**
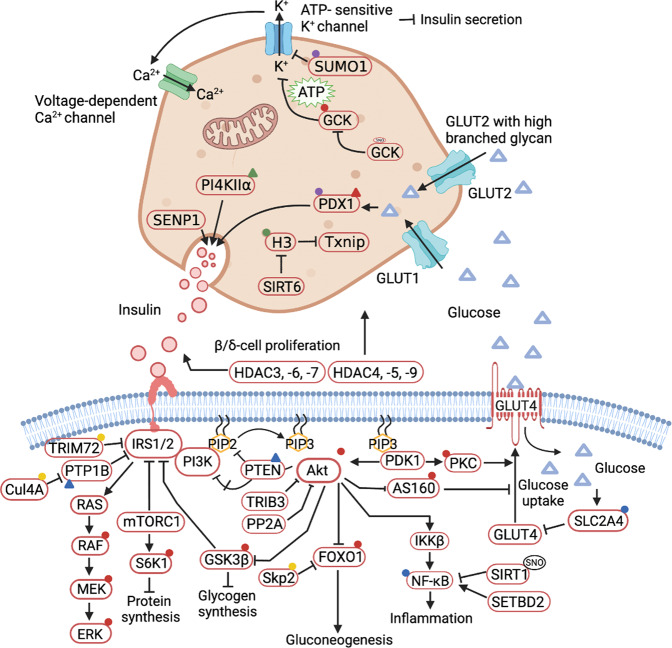
A holistic summary and illustration of the crosstalk between PTMs and diabetes. The pathogenesis of diabetes is complex and interactive, involving various cellular responses and signaling cascades regulated by PTMs. (**1**) Balance the actions of kinases and phosphatases in regulating glucose-stimulated insulin secretion from pancreatic beta cells. (**2**) Establish the link between gluconeogenesis, the TCA cycle and glycolysis. (**3**) Directly cause modification of certain proteins or induce PTMs secondary to various cellular processes to maintain beta cell function and viability. Different colors and shapes represent different types of PTMs. Activation and inhibition effects are displayed in “arrows” and “inhibition” symbols, respectively. The figure is generated with BioRender (https://biorender.com). CUL4A cullin 4A, ERK extracellular regulated protein kinases, FOXO1 forkhead box O1, GCK glucokinase, GLUT glucose transporter, GSK-3 glycogen synthase kinase 3, IRS insulin receptor substrate, HDAC histone deacetylase inhibitor, JNK c-Jun N-terminal kinase, MKP1 mitogen-activated protein kinase phosphatase, MEKK mitogen-activated extracellular signal-regulated kinase kinase, NF-κB nuclear factor-k-gene binding, PTP1B protein tyrosine phosphatase 1B, PTEN phosphatase and tensin homolog, PPAR peroxisome proliferators-activated receptors, PDK1 3-phosphoinositide-dependent protein kinase 1, PP2A proteinphosphatase2A, SENP2 sentrin-specific protease 2, SREBP1 sterol-regulatory element binding protein 1, SIRT sirtuins, TRIB3 tribbles pseudokinase 3, TXNIP thioredoxin interacting protein

Diabetes occurs in several major forms, including type 1 diabetes mellitus (T1DM), type 2 diabetes mellitus (T2DM), and gestational diabetes mellitus (GDM) and others, each of which is diagnosed and characterised by hyperglycemia. Diabetes has a strong association with the development and progression of life-threatening CVD. The processes of diabetes are intricate and interactive, including various cellular responses and signaling cascades regulated by PTMs. For example, various kinases and phosphatases regulate glucose-stimulated insulin secretion in pancreatic beta cells. PTMs can establish the link between gluconeogenesis, the TCA cycle and glycolysis to affect beta cell viability and function. In this section, we discuss the roles of various PTMs in diabetes (Fig. 3).

#### Phosphorylation in diabetes

Protein phosphorylation is an important PTM that balances the actions of kinases and phosphatases in regulating glucose-stimulated insulin secretion from pancreatic beta cells. Glucose homeostasis is mainly dependent on signaling cascades mediated by protein kinases and phosphatases which determine the output of metabolic processes by controlling PTMs of different substrates. The insulin receptor (INSR, IR) activates various downstream targets, such as PI3K/AKT (PKB), mitogen-activated protein kinases 3/1 (MAPK3/1), extracellular signal-regulating kinase 1/2 (ERK1/2) and AMP-activated protein kinase (AMPK), to control energy homeostasis and stimulate energy catabolic processes. Thus, the PI3K/AKT, MAPK and AMPK pathways are required for insulin-dependent regulation of metabolic activity. As exemplified above, the insulin-PI3K/AKT pathway is negatively regulated by PTPN1 (PTP1B), PTEN and PP2A, which can dephosphorylate and inhibit IR, IRS1/2, PIP3, and AKT. Thus, PTMs of proteins of the insulin signaling pathway can impair or improve metabolic pathways.

Phosphorylation events and kinases in islets are associated with insulin secretion. Based on the SILAC proteomics, 8539 phosphosites derived from 2487 proteins were identified in the islets, and 170 phosphosites (98 were upregulated and 72 were downregulated) are differentially expressed in response to a short-term high glucose challenge.

IR is essential for insulin action and plays an important role in pancreatic cells. Deletion of IR reduced β and α cell mass and induced hyperglycaemia in mice. *Ins1*^-/-^:*Ins2*^f/f^ mouse β cells lose about 50% of insulin production, resulting in robust hyperglycemia, β cell proliferation, hormone expression disorders and alleviation of ER stress. This is associated with hyperphosphorylation of AKT, leading to reduced DNA damage inducible transcript 3 (DDIT3), tribbles pseudokinase 3 (TTIB3), activating transcription factor 4 (ATF4) and phospho-eIF2α expression.^208^ Overexpression and activation of AKT in pancreatic cells regulate the phosphorylation/dephosphorylation of signaling factors such as forkhead box O1 (FOXO1), glycogen synthase kinase 3β (GSK3β) and mammalian target of rapamycin 1 (mTORC1) and its downstream target to regulate β cell mass and proliferation.^209^ Previous studies have established that the overactivity of AKT is sufficient to increase the proliferation of β-cells via cyclin D1.^209,210^

AMPK is the most intensively studied protein kinase in the treatment of metabolic syndrome. Activated AMPK phosphorylates substrates and can stimulate glucose uptake and inhibit glycogen synthesis.

PTP1B and PTEN antagonize insulin signaling by dephosphorylating the IR and IRS1/2. PTP1B deficiency and the partial reduction of Pten results in improved glucose tolerance and protects against insulin resistance in mice.^211^

A study found that protein phosphatase-2C alpha (PP2C alpha) directly dephosphorylated the p85 subunit of PI3K to stimulate its catalytic activity and enhance insulin sensitivity. Heart- and liver-specific knockout of Ppp2r2a increases the phosphorylation of important insulin signaling molecules, such as AKT, GSK-3α/β, FOXO1 and GS, resulting in increased insulin sensitivity and improved glucose tolerance in the heart and liver.^212^

In addition, the data also indicated that all PKA and PKC substrates in the *db/db* mouse islets were dephosphorylated and that a hyperglycemic environment can increase the phosphorylation of the β cell-specific transcription factor PDX1 through GSK3 kinase, leading to β cell failure.^19^

Serine/Threonine Kinase 25 (STK25) and CK2 are both serine/threonine kinases. Overexpression of STK25 is known to aggravate muscle insulin resistance and increase intramyocellular lipid accumulation.^213^ Inhibition of CK2 reduced the phosphorylation of class I HDACs to activate adipocyte thermogenesis and protected mice from diet-induced obesity and insulin resistance.^214^

#### Acetylation in diabetes

The acetylation of proteins is a pathway that is a reversible PTM regulated through the function of specific types of enzymes and this process functions as a main regulator of human metabolism. Acetylation is a PTM dependent on acetylases and deacetylases for catalyzing acetylation and deacetylation processes, respectively. Acetyl-CoA provides acetyl groups for acetylation and acts as an essential constituent of gluconeogenesis, the TCA cycle and glycolysis. As a consequence, there is an established link between acetylation and hyperglycemia and the insulin resistance of metabolic syndrome.

The differential expression of NAD-dependent deacetylase SIRT3 between Goto-Kakizaki (GK) rats and nondiabetic Brown Norway (BN) rats can support the causality between protein acetylation and impaired glucose homeostasis.

The spectrum study of lysine acetylation in the diabetic kidney identified 39 differentially expressed proteins, most of which were intermediate metabolic enzymes.^215^ Hyperglycemia-induced acetylation of retinal histones H3 and H4 regulates the activities of several proinflammatory proteins that participate in the pathogenesis of diabetic retinopathy (DR). HFD feeding can enhance acetylation of the fatty acid β-oxidation enzymes β-hydroxylacyl coenzyme A dehydrogenase (β-HAD) and long-chain acyl-CoA dehydrogenase (LCAD); it can also lead to the dysregulation of the insulin signaling pathway. In a mutant mouse model of CREB-binding protein (CBP), increased insulin sensitivity and glucose tolerance were demonstrated. A monogenic autosomal form of T2DM,MODY (Maturity Onset Diabetes of the Young), was determined to be associated with histone acetyltransferase (HATs) and HDACs. Some HDAC inhibitors can improve insulin resistance to ameliorate inflammation. Some HDAC3 inhibitors can improve glycemia, promote insulin secretion and protect β cell function in the prediabetic stage.^26,216^ HDAC4, HDAC5, and HDAC9 are key regulators that promote the development of the β/δ-cell lineage. Moreover, inhibition of HDAC6 in pancreatic islets downregulates insulin signaling. Increased HDAC7 levels impair insulin secretion and contribute to β cell dysfunction in type 2 diabetic islets. SIRT6 mediates the deacetylation of histone H3 to restrain Txnip expression in beta cells, thereby maintaining beta cell function and viability.

Notably, compounds that modify lysine acetylation, such as resveratrol, are known to inhibit early diabetic retinopathy in diabetes.^217^

#### Methylation in diabetes

Histone methylation and nonhistone protein methylation are all types of PTMs termed methylation. Protein methylation mainly occurs at lysine or arginine residues and is appended with either one to three methyl groups by N-methyltransferase in the cytosol. Protein methylation is often associated with gene repression or activation depending on the degree and position of the modifications.

Some work has been done on diabetes-associated biochemical modification of metabolic enzymes via methylation. The expression of nicotinamide N-methyltransferase (NNMT) is increased in the white adipose tissue (WAT) and liver tissue of patients with insulin resistance or T2DM. Deletion of NNMT in the livers of C57BL6/J mice lowers fasting plasma glucose levels.^218^ The histone methyltransferase SETDB2-associated pathway IFN-β-SETDB2-H3K9me3 is dysfunctional in diabetes and induces nuclear factor kappa B (NF-κB)-mediated inflammation.^219^ The lack of histone methyltransferase G9a suppresses CD36 and M1 macrophage genes in type 2 diabetic patients.^220^ PRMT1 plays an essential role in maintaining mature β-cell identity.^35^ Deficiency of PRMT5 impairs glucose tolerance and glucose-stimulated insulin secretion in a mouse model. However, the compensatory increase in H3R8me2 can accelerate the binding of the brahma-related gene-1 (BRG1) chromatin remodeling enzyme to the insulin gene promoter.^221^

Patients with T1DM have an increased demethylation level of H3K9me2 in blood lymphocytes.^222^ Methylation of H3K4me1 was increased in patients with T2DM in the transcription factor NF-κB promoter region.^223^

T1DM and T2DM show increased H3K9me3 of the Slc2a4 promoter and decrease glucose transporter type 4 (GLUT4) expression, thus contributing to glycemic impairment.^224^ KDM6A, one of the known H3K27me2/3 demethylases, has higher protein levels in the kidneys of diabetic OVE26 mice.^224^ Combination therapy with telmisartan and esculetin, attenuates increased levels of histone PTMs such as H3K9me2, H3K9Ac, H2AK119Ub, and H2BK120Ub in type 2 diabetic cardiomyopathy.^225^

#### Ubiquitination in diabetes

Ubiquitination is a PTM resulting from the covalent linking of each successive ubiquitin to the previous ubiquitin at lysine by polyubiquitination or mono-ubiquitination. Ubiquitin-activating enzyme (E1), ubiquitin-conjugating enzyme (E2) and ubiquitin ligase enzyme (E3) coordinate the action of ubiquitin-proteasome system homeostasis and degradation.

Inhibition of ubiquitin-activating enzyme E1 blocks ubiquitination of the key molecules of insulin signaling and prevents palmitate-inducible insulin resistance.^226^ UBC9 protein expression is decreased in muscle tissues from T2DM patients.^227^ Haploinsufficiency of UBC13 can ameliorate HFD-induced insulin resistance.^228^ Ubiquitin-conjugating enzyme E2O (UBE2O) is significantly upregulated in obese individuals with T2DM.^229^

The Really Interesting New Gene (RING) family is emerging as the most important ubiquitin ligase. RING ligases play a crucial part in PI3K/AKT-mediated glucose metabolism. Cullin-RING ligase complexes (CRLs) are the most abundant RING E3 ligases. SKP2 (substrate of CRL1) can ubiquitinate and promote the degradation of FOXO1. Cul4A-RING E3 ubiquitin ligase suppresses PTP1B activity and suppresses the expression of genes associated with gluconeogenesis.^230^ TRIM family proteins are involved in the progression of diabetes and the development of diabetic complications. For instance, TRIM13 attenuates DN-induced collagen synthesis by promoting the ubiquitination of C/EBP homologous protein (CHOP).^231^ TRIM32 inhibition increased PI3K-AKT-FOXO signaling in the liver and skeletal muscle and enhanced glucose uptake.^232^ Mitsugumin 53/ TRIM72 promotes ubiquitin-dependent degradation of the insulin receptor and insulin receptor substrate-1, resulting in T2DM.^46^ E3 ubiquitin ligase F-box and WD repeat domain-containing 7 (FBW7) boosted EZH2 ubiquitination and proteasome degradation to inhibit tumor necrosis factor-α (TNF-α)-induced pancreatic β-cell apoptosis. FBW7 inhibits T1DM via the EZH2/ZBTB16 axis in vivo and in vitro.^233^

In addition, chronic hyperglycemia-induced oxidative stress can lead to ER stress and defective insulin secretion by disturbing the ubiquitin-proteasome system. Sections of pancreatic tissues from Zucker diabetic fatty rats show that a large number of Ub-proteins formed in the cytosol of pancreatic cells and β-cells. This response may promote autophagy to protect β-cells from cellular damage during hyperglycemia. Genetic deletion of thioredoxin-interacting protein (Txnip) in cells can increase protein ubiquitination of Xbp1, decrease gluconeogenesis and increase insulin sensitivity.^234^ An increased level of UBE2v1- and Lys63-ubiquitinated proteins was found in patients with T2DM, and the latter is involved in the pathological process of tubular damage in diabetic nephropathy.^235^

Several drugs can ameliorate diabetes by modulating protein ubiquitination. For example, inhibition of progestin and adipoQ receptor 3 (PAQR3) mediates STUB1-peroxisome-proliferator-activated receptor γ (PPARγ) protein ubiquitination and degradation to accelerate diabetic wound healing.^236^

#### Sumoylation in diabetes

SUMOylation is an evolutionarily conserved PTM in which a SUMO is covalently attached to the lysine (K) residue of target proteins.^237^ The SUMOylation process involves an activating enzyme 1 (E1, Uba2/Aos1), conjugating enzyme 2 (E2, UBC9), SUMO ligation enzyme 3 (E3, such as PIAS, RanBP2, and Pc2), and SENPs responsible for deSUMOylation. Sumoylation regulates diverse biological processes.

Type 2 diabetic patients with severe insulin resistance have lower UBC9 protein expression in skeletal muscle.^227^ Mice depleted of the unique SUMO conjugation E2 enzyme UBC9 in pancreatic beta cells spontaneously develop diabetes because of β cell death occurring as a result of the accumulation of reactive oxygen species.^238^ Gli-similar 3 (Glis3) is an insulin-regulated-associated transcription factor. Interestingly, PIAS-family proteins and UBC9 can sumoylate Glis3 to downregulate insulin transcription.^239^

The SUMO deconjugation enzyme SENP1 is involved in insulin secretion in T2DM and adipocyte inflammation in T1DM. SENP1 is localized with insulin granules in β cells, and deletion of SENP1 in β cells of mice impaired glucose tolerance.^240^ Adipocyte-specific deletion of SENP1 aggravated the SUMOylation of the NF-κB essential molecule (NEMO) and symptoms of T1DM.^241^

SUMOylation is associated with the incidence of T1DM in Asian populations.^242^ SUMOylation can also regulate β cell function to prevent the development of diabetes. Beta cells cultured in low glucose (2 mM) media show increased SUMOylation of MafA and interference with the transcription of the insulin gene. A high glucose environment increases the SUMOylation of PDX-1 to enhance insulin gene expression.^243^

SUMOylation affects insulin exocytosis. SUMO1 inhibits the voltage-dependent K^(+)^ (Kv) channel Kv2.1, leading to decreased β-cell excitability and insulin exocytosis.^244^ SUMO1 blunts the exocytotic response of β-cells to Ca^2+^ to decrease glucose-stimulated insulin secretion.^245^

Based on this evidence, regulators of SUMOylation deserve additional study in the context of PTM and metabolic disease.

#### Neddylation in diabetes

Cullin neddylation is a process mediated by NEDD8-E1, E2, and E3 enzymes that sequentially transfer NEDD8 to a cullin protein. Inhibition of cullin neddylation rapidly decreases hepatic glucose generation, attenuates hyperglycemia and improves hepatic insulin signaling in mice. Dysfunction of Cullin 3 RING E3 ubiquitin ligase causes vasoconstriction and increased sodium reabsorption in diabetes^64^

#### Glycosylation in diabetes

Glycosylation includes glycosyltransferases and glycosidases. N-glycosylation is a subtype of glycosylation where polynucleotides and polypeptides are linked with asparagine by an N-glycosidic bond. Increased levels of highly branched N-glycans in plasma indicate an increased risk of diabetes.^246^

Reduced Glut-2 murine N-glycosylation and GlcNAcT-IV expression are associated with diabetes induced by a high-fat diet.^247^ GlycA is identified as a marker of systemic inflammation that originates from N-acetylglucosamine, and systemic inflammation may likely contribute to T2DM occurrence by causing insulin resistance and β cell dysfunction.^248^ Supplementation with sialic acid or the sialic acid precursor N-acetyl-D-mannosamine may restore anti-inflammatory properties and preserve insulin sensitivity.^249^

In other types of diabetes, fucosylated N-glycans are a novel biomarker of HNF1A-MODY, and N-glycans of human milk lactoferrin and secretory immunoglobulin A have been altered in gestational diabetes mellitus.^250^ Unlike the traditional forms of glycosylation, O-GlcNAcylation is an O-linked β-N-acetylglucosamine (O-GlcNAc) group covalently bound to threonine and/or serine residues of proteins. O-GlcNAc transferase (OGT) and O-GlcNAcase (OGA) control the dynamic cycling of O-GlcNAcylation. An early study found that the incidence and age of incidence of T2DM were linked with a region on chromosome 10q in the Mexican American population.^251^ The SNP of the enzyme O-GlcNAcase encoded by MGEA5 on 10q24.1-q24.3 may increase diabetes risk in Mexican Americans.^252^

OGT is highly expressed in islets. O-GlcNAcylation is essential for the function and survival of β cells. β-cell specific OGT-KO mice cannot maintain glucose homeostasis and regulate pancreatic β-cell function. OGA overexpression in β cells decreases insulin secretion and impairs glucose tolerance.^253^ Notably, the knockout phenotype of *oga-1* (*Oga*^-/-^) is similar to human T2DM.^254^

Glucose increases the O-GlcNAcylation of Pdx-1 to increase its DNA binding to the A-box in the HR2 region of the GPR40 promoter to stimulate insulin secretion.^255^

Elevated O-GlcNAc-modified protein levels not only affect pancreatic islets but also affect kidney cells and cardiac, liver, muscle and fat tissues. The increased O-GlcNAcylation extent of cytoskeletal proteins (α-actin, α-tubulin, actinin 4, myosin) is associated with morphological changes in the diabetic kidney. Elevated O-GlcNAcylation in the liver can respond to hyperglycemia by accelerating gluconeogenesis/de novo lipogenesis through FoxO1, PGC-1α, CRTC2, carbohydrate-responsive element-binding protein (ChREBP) and liver X receptor (LXR).

GlcNAcylation can reflect the glycemic status at individual sites based on solid-phase chemical derivatization and chemoenzymatic tagging. O-GlcNAc modification can be a potential biomarker and assist in the identification of prediabetic patients.

#### Palmitoylation in diabetes

Protein palmitoylation is defined as the process by which palmitic acid molecules reversibly attach to cysteine residues via thioester bonds. Palmitoylation has been implicated in the metabolic dysregulation of islet β-cells. Previously, loss of the small GTPase ARF family member ARL15 gene reduced insulin secretion in a human β-cell line. ARL15 is located in the Golgi network, indicating a palmitoylation-dependent Golgi-based role.^81^ Phosphatidylinositol 4-kinase II-alpha (PI4KIIα) palmitoylation positively contributes to enhancing insulin signaling.^256^

#### Myristoylation in diabetes

Myristoylation is a crucial fatty acid acylation catalyzed by NMTs, which can add a myristoyl group to an amino-terminal glycine residue of a protein.^84^ STZ-induced diabetic animal results in a two-fold increase in liver NMT activity in animals and sodium orthovanadate can normalize liver NMT activity in STZ-induced diabetic rats. Furthermore, liver NMT activity is inversely proportional to plasma insulin levels. The effects of diabetes on NMT remain unclear.

#### Prenylation in diabetes

Prenylation includes protein farnesylation and geranylgeranylation, which are catalyzed by farnesyl transferase (FTase) and geranylgeranyl transferase (GGTase), respectively. Several pharmacological and molecular biological experiments have indicated that protein prenylation represents a committed step in glucose-stimulated insulin secretion. Mevalonic acid (MVA) is a precursor for the biosynthesis of FPP and GGPP. HMG-CoA reductase inhibitors (statins) inhibit the synthesis of MVA and sequentially inhibit GSIS. Knockdown of the FTase-β subunit suppresses insulin release.^95^

#### S-glutathionylation in diabetes

S-glutathionylation is a modification of cysteine and the disulfide bond formed only between the cysteine of protein. Protein S-glutathionylation mediates thiol redox signaling and likely plays a significant role in the pathogenesis of diabetes.^106,108^ S-glutathionylation at residue C215 of PTP1B can modify insulin signaling, leading to decreased activity. In addition to PTP1B, PTEN also undergoes S-glutathionylation and influences the PI3K-Akt pathway in hepatocytes from rats fed an HFD. S-glutathionylation, which is involved in the deactivation of Akt and downstream of Akt, is an inhibitor of IKKβ that can regulate insulin resistance in diabetes. Furthermore, S-glutathionylation of hemoglobin of diabetic patients was found to be increased in blood samples.^257^

#### S-nitrosylation in diabetes

Protein S-nitrosylation (SNO) is a reversible modification of cysteine thiols mediated by NO. S-nitrosylation plays an important role in the pathogenesis of insulin resistance. S-nitrosylation of insulin signaling molecules is elevated in patients with T2DM. Protein-nitrosylation was observed in diabetic rats and led to mitochondrial dysfunction. S-nitrosylation is involved in insulin resistance by activating and inactivating Akt. Nitric oxide (NO) inhibits the Abeta-degrading activities of insulin-degrading enzyme (IDE) through S-nitrosylation.^258^ Aspirin treatment reduces iNOS protein levels and S-nitrosylation of IRbeta, IRS-1 and Akt to improve insulin resistance and signaling.^118^

To date, multiple functions of protein S-nitrosylation have been associated with the fate of insulin. S-nitrosylation of Ryanodine Receptor 2 (RyR2) promotes calcium release and insulin secretion. Hyper-nitrosylation of RyR2 in β-cells impairs GSIS and blood glucose clearance.^259^ Sulfonylurea receptor (SUR) is a component of KATP channels, and its S-nitrosylation inhibits ATP binding. Gain-of-function SUR1 mutations lead to neonatal diabetes.^260^

#### Sulfhydration in diabetes

S-sulfhydration is similar to S-nitrosylation in some chemical features. H_2_S, a novel gasotransmitter, acts as a major donor for protein S-sulfhydration. S-sulfhydration modifies cysteines in proteins to mediate most cellular responses. H_2_S increased S-sulfhydration of kelch like ech associated protein 1 (KEAP1) and nuclear erythroid 2-related factor 2 (NRF2) nuclear translocation to attenuate diabetes-accelerated atherosclerosis.^126^

#### Citrullination in diabetes

Citrullination is catalyzed by the PAD enzyme family. A recent study has shown that daily hypodermic injections of BB-Cl-amidine (a pan-PAD inhibitor) can protect against the onset of diabetes in NOD mice.^261^ Moreover, citrullination caused by inflammation occurs almost exclusively in the pancreas and can be considered a marker of beta cell dysfunction or T1D.^137^

#### ADP-ribosylation in diabetes

ADP-ribosylation is a PTM catalyzed by the enzymatic transfer of ADP-ribose from NAD^+^ onto target proteins. This PTM usually occurs on cysteine, arginine and asparagine residues. PARP was demonstrated to be a pathogenic marker of diabetes and diabetic complications in vitro and in vivo. Hyperglycemia increases PARP activation in diabetic patients and decreases GAPDH and α-enolase enzymatic activity. NAD^+^ is used as a substrate for ADP ribosylation. Activated PARP can cause the depletion of NAD^+^ to destroy islet cells. The destruction of the PARP gene completely protected mice from diabetes.^262^

#### Carbonylation in diabetes

Carbonylation is induced by the oxidative stress generated by activated platelets. T2DM additionally enhances carbonylation of human platelet proteins. Rat model of T1DM loss the activity of SERCA2a and diastolic dysfunction occurs by carbonylation.^154^

### PTMs in obesity

**Fig. 4:**
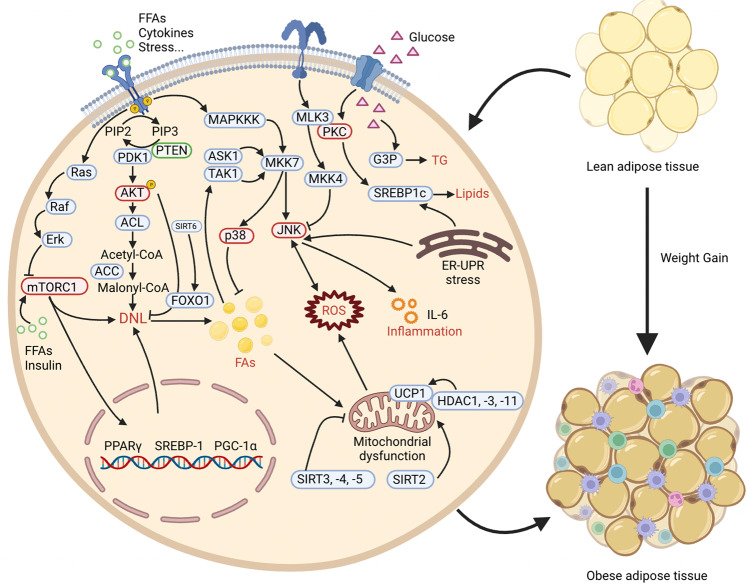
Overview of the roles of PTM in obesity. Obesity is associated with excess calorie intake leading to excessive accumulation of adipose tissues. PTMs can regulate the activity of enzymes or cytokines associated with obesity, thereby engaging the occurrence and treatment of obesity-related metabolic diseases. (**1**) High glucose levels, for example after ingestion of carbohydrates, trigger the secretion of insulin from pancreatic β cells and the activation of a series of kinases downstream of PI3K, such as Akt, PKC, and mTORC1, which stimulates glucose uptake and utilization, and generate FAs that are taken up by adipose tissue. (**2**) Activated transcription factors, including SREBP-1, PPARγ and PGC-1α, for transcriptional activation to promote de novo lipogenesis. (**3**) Some HDAC family members inhibit the thermogenic program in BAT to regulate HFD-induced leptin resistance and obesity. Activation and inhibition effects are displayed in “arrows” and “inhibitors”, respectively. The figure is generated with BioRender (https://biorender.com). AKT protein kinase B, ACL ATP citrate lyase, ACC acetyl CoA carboxylase, FFAs free fatty acids, FOXO1 forkhead box O1, G3P glyceraldehyde 3-phosphate, JNK c-Jun N-terminal kinase, mTOR mechanistic target of rapamycin, PIP2 phosphati-dylinositol-4,5-bisphosphate, PDK1 3-phosphoinositide-dependent protein kinase 1, PTEN phosphatase and tensin homolog, PGC1 peroxisome proliferators-activated receptorγcoactivator 1, PPARγ peroxisome proliferator-activated receptor

Obesity has much to do with excess calorie intake leading to excessive accumulation of adipose tissues. PTMs can regulate the activity of enzymes or cytokines connected with obesity, thereby engaging the treatment of obesity-related metabolic diseases. High fat and glucose levels can trigger the secretion of insulin from pancreatic β cells and PTMs of a series of kinases, generate fatty acids that are taken up by adipose tissue and induce obesity. (Fig. 4).

#### Phosphorylation in obesity

Obesity is a metabolic disease induced in its simplest manifestation by an imbalance between energy intake and expenditure. Chronic nutritional excess leads to adipocyte hypertrophy, which further promotes obesity-associated diseases.

Protein kinases regulate a number of biological processes by phosphorylation. Human GSK3β-overexpressing mice had greater body weight due to an increase in fat mass.^263^ Inactivation of GSK3β by Dyrk1A phosphorylation suppresses the expression of adipogenic proteins, potentially playing a part in the pathological process of obesity. Deficiency of MAP kinase interacting serine/threonine kinase 1 (MNK1) or MNK2 can protect against HFD-induced weight gain.^264^

Phosphatidylinositol-3,4,5-triphosphate [PtdIns(3,4,5)P_3_] is one of the most important phosphoinositides (PIs), and Akt is the most well-known target. The phosphorylation of AKT is catalyzed by PtdIns(3,4,5)P_3_. AKT-dependent FOXO1 phosphorylation decreased in DIO mice. Inhibition of hepatic atypical protein kinase C (aPKC) decreased excessive expression of lipogenic enzymes and improved weight gain.^265^ Activation of AKT2 mediates the stimulation of de novo lipogenesis. The phosphorylation of p66Shc is associated with obesity induced by excess nutrients.^266^

The deficiency of MARK4, an AMPK-related family member, enhances insulin-stimulated AKT phosphorylation to activate brown fat to diminish diet-induced obesity.^267^ Leptin, insulin and glucose adjust food intake by (de)phosphorylation of hypothalamic AMPK. Leptin regulation has been linked to PTP1B. Neuronal PTP1B knockout mice have lower body and adiposity weight, but adipose PTP1B deficiency increases body weight.^268^ JAK2 is a downstream effector of the leptin receptor, and its dephosphorylation depends on PTP1B to stimulate appetite-associated hormones. The biological activity of leptin can also be regulated by STAT3. STAT3 phosphorylation deficiency in the hypothalamus results in central leptin-induced resistance and obesity.^269^

The underlying causes of obesity may be cellular lipid and glucose imbalance or dysregulation. Further research on protein phosphorylation is required to determine its role as a target for treating obesity-associated metabolic diseases.

#### Acetylation in obesity

Protein acetylation is especially relevant to obesity. Protein acetylation is a component of a variety of metabolic reactions, such as glucose metabolism, the TCA cycle and fatty acid pathway. The dynamic regulation of white adipose tissue (WAT), brown adipose tissue (BAT) and beige adipose tissue can affect body obesity to a great extent. The levels of histone H3 lysine 9 and 18 acetylation at the *Tnfα* and *Ccl2* genes are upregulated in obese mouse livers.^270^

Type I HDACs have deacetylation domains. Some studies demonstrated that HDAC1 inhibits the thermogenic program in BAT through the deacetylation of H3K27.^271^ Acute strenuous exercise can induce hyperacetylation of H4 and decrease HDAC2 activity in LPS-stimulated peripheral blood mononuclear cells (PBMCs) of obese males.^272^HDAC3 acts as a coactivator of oestrogen-related receptor α (ERRα) to maintain the capacity for thermogenesis in BAT by deacetylating PGC-1α, ERRα and UCP1.^273,274^ HDAC5 regulates HFD-induced leptin resistance and obesity via STAT3 deacetylation at Lys685 to improve the effect of leptin in the hypothalamus.^275^ HDAC6 and acetylated α-tubulin also control adipogenesis.^276^ In diet-induced obesity mice, the lipogenic transcription factor sterol regulatory element-binding protein-1 (SREBP-1) directly upregulated HDAC8 to promote insulin resistance.^277^ HDAC9 is associated with adipocyte differentiation and obesity. HDAC11 inhibits the expression of UCP1 in BAT to be a novel regulator of obesity.^278^

Type III HDACs include SIRT1-7. SIRT1 can accelerate the deacetylation of PPARγ to induce the browning of WAT.^279^ SIRT2 deacetylates the p65 subunit of NF-κB and RIP-1.^280^ SIRT3 and SIRT4 are located in mitochondria and regulate energy expenditure.^281^ SIRT5 and related acylation can reduce liver steatosis in *ob/ob* mice.^282^ SIRT6 is a FOXO1 deacetylase that drives lipid catabolism.^283^

As mentioned above, protein acetylation, energy metabolism and adiposity go hand in hand.

#### Methylation in obesity

An obesity study quantified histone methylation in diet-induced obesity mice. The study identified 4 glutamate methylation sites and 1 histidine methylation site with statistical significance. Among them, H2A E67me1 and H4 E74me1 might be associated with the pathological process of obesity.

Methylations of H3K4, H3K36, and H3K79 can activate transcription, while H3K9, H3K27, and H4K20 methylation can suppress transcription. Reversible histone methylation is catalyzed by histone methyltransferases (HMTs) and histone demethylases (HDMs), which have been shown to regulate energy metabolism.^284^

Lysine-specific demethylase 1 (LSD1) is the first identified HDM that demethylates H3K4 monomethylation/dimethylation (H3K4me1/me2) and can also reverse methylation of H3K9me1/me2.^285^

EHMT1 and EHMT2 are H3K9 methyltransferases. EHMT1 expression positively regulates brown adipose energy homeostasis by stabilizing the PRDM16 protein and depositing the suppressive H3K9me2 and H3K9me3.^286^ Specific deletion of adipose EHMT1 leads to obesity, systemic insulin resistance and adaptive thermogenesis.^286^ Lacking muscle-specific EHMT2 are resistant to high-fat diet (HFD)-induced obesity and hepatic steatosis in female mice.^287^ JMJD1A is another H3K9 demethylase that binds to the Ucp1 gene and decreases levels of H3K9me2 to regulate metabolic gene expression and obesity resistance.^288^

KMT5c catalyze the methylation of H4K20. Kmt5c knockout mice with decreased repressive marker H4K20me3 are obese when fed with an HFD and develop glucose intolerance.^289^

In the methylation of H3K27, enhancer of zeste homolog 2 (EZH2) promotes adipogenic differentiation, body weight and adipose tissue mass by catalyzing trimethylation of H3K27.^290^

Disruption of telomeric silencing-1 like (DOT-1L) regulates the BAT-selective gene program by promoting H3K79 methylation, especially H3K79me2 modification. Deletion of DOT-1L in thermogenic adipocytes can protect mice from diet-induced obesity.^291^

#### Ubiquitination in obesity

In obesity, adipocyte differentiation is controlled by numerous transcriptional cascades. PPARγ is a nuclear receptor that is associated with obesity and metabolic diseases by converting adipocytes from their precursors. E3 ubiquitin ligase-mediated protein ubiquitination and proteasome-dependent degradation of PPARγ gradually exhibited clear mechanisms in the development of obesity.

Ubiquitin-conjugating enzymes act as important factors affecting the process of ubiquitination. UBE2O is expressed preferentially in metabolic tissues. *Ubc2o*^-/-^mice showed a distinct reduction in overall fat mass and showed a reduction in body weight relative to their WT counterparts.^229^ Another conjugating enzyme simultaneously catalyzed ISGylation and ubiquitination reactions called UBE2L6, which were upregulated in WAT from obese humans and mice. Deficiency of adipose-specific Ube2l6 stabilizes ATGL protein and reduces HFD-induced obesity and insulin resistance.^292^

Knockdown of the E3 ubiquitin ligase IDOL in mice decreased circulating levels of cholesterol, triglycerides, hepatosteatosis and fat mass.^293^ Deletion of TRIM28 induces obesity via epigenetic mechanisms in embryonic development.^294^ TRIM72/MG53 deficiency targets the insulin receptor and IRS1 protein to attenuate HFD-induced obesity.^295^

PPARγ-mediated ubiquitination and degradation of lysine 150 of selenoprotein S (SelS) and lysine 47-48 of selenoprotein K (SelK) is required for adipocyte differentiation. CUL2-APPBP2 is the ubiquitin E3 ligase of the cullin–RING member family. CUL2 stabilized PRDM16 protein to repress adipocyte thermogenesis and counteracted diet-induced obesity by catalyzing its polyubiquitination.^153^ A study showed that fatty acid binding protein 4 (FABP4) was higher in the adipose tissues of obese diabetic patients. FABP4 regulates adipogenesis by downregulating PPARγ and attenuates the development of diet-induced obesity in mice.^296^

#### SUMOylation in obesity

The SUMOylation modification is reversible, in which the modified proteins can be deSUMOylated by SENPs. SUMO regulation is strongly associated with various diseases.

Depletion of UBC9 induces the expression of brown fat genes in human subcutaneous adipocytes.^297^

SUMO-specific protease SENP1 deficiency leads to hyper-SUMOylation of SIRT3, which can protect mice from HFD-induced obesity by increasing oxidative phosphorylation and energy expenditure.^298^ SENP2 deSUMOylated PPARα and promoted its ubiquitylation, which in turn inhibited FGF21 expression and fatty acid oxidation.^299^ Meanwhile, FGF21 null mice are lipodystrophy and have less body fat, which is associated with PPARγ SUMOylation at lysine 107.

Krüppel-like transcription factor 5 (KLF5) is also an essential regulator of lipid metabolism and is controlled by SUMOylation.^300^

#### Neddylation in obesity

NEDD8-based neddylation of PPARγ is crucial to conjugating and stabilizing PPARγ during adipogenesis and provides a potential anti-obesity therapeutic strategy that targets the neddylation of PPARγ. It remains to be investigated if the Neddylation signature in obesity is pathophysiologically relevant in obese animal models and human patients.

#### Glycosylation in obesity

N-glycosylation affects obesity-associated protein structure and function. Studies have shown that central obesity is involved in changes in IgG N-glycosylation. A low-calorie diet induced a marked effect on IgG N-glycosylation.^301^

O-GlcNAcylation of protein is a nutrient-sensing and cellular stress response. Excessive nutritional intake leads to metabolic disorders, including obesity and diabetes. Chronic ingestion of a high-fat diet can increase O-GlcNAc levels in cerebral arteries and the heart. It follows that there is a role for O-GlcNAc signaling in DIO and metabolic dysfunction. The levels of O-GlcNAcylation are determined by O-GlcNAc transferase (OGT) and O-GlcNAcase (OGA). OGT targets adipose lipid desaturation to drive obesity, and the deletion of adipocyte OGT abolishes HFD-induced hyperphagia and obesity in mice.^302^ Loss of OGT can also decrease O-GlcNAcylation of lipid droplet-associated perilipin 1 (PLIN1), elevate PLIN1 phosphorylation and promote lipolysis in visceral fat to relieve diet-induced obesity.^303^

#### Palmitoylation in obesity

A palm oil-rich diet (HPD) causes obesity by inducing dynamic protein S-palmitoylation. It remains to be investigated if the palmitoylation signature is relevant to obesity in animal models and human subjects.

#### Myristoylation in obesity

Saturated FA activates Jun N-terminal kinase (JNK) by altering the membrane distribution of c-Src (a myristoylated protein) to affect obesity in mice and men.^304^

#### Prenylation in obesity

Mammalian geranylgeranyl diphosphate (GGPP) synthase catalyzed the synthesis of isoprenoid moieties for protein isoprenylation, whose expression is regulated in obesity and adipogenesis.^305^ Statins are a useful tool to investigate the role of prenylation in diabetes.

#### S-glutathionylation in obesity

S-glutathionylation, in which reactive oxygen species (ROS) react with cysteine residues of proteins to form glutathione (GSH), is removed by glutaredoxin-1 (Glrx). A previous study indicated that Glrx-lacking mice had increased protein S-glutathionylation and developed obesity; however, the mechanism is unknown.^306^ Another study indicated that deficiency of Glrx stabilized and increased C/EBPβ protein levels to stimulate 3T3L1 cell differentiation and adipogenesis.^307^

#### S-nitrosylation in obesity

In obese humans and DIO mice or *ob/ob* mice, total protein S-nitrosylation is increased. For example, the increased S-nitrosylation of PDE3B was detected in adipose tissue of DIO mice, which means PDE3B may be a specific target in adipocytes. S-Nitrosoglutathione reductase (GSNOR; alcohol dehydrogenase 5 [ADH5]) controls BAT homeostasis to regulate adipose thermogenesis.^308^

On the other hand, obesity-associated inflammation relates to endoplasmic reticulum dysfunction by S-nitrosylation. Obesity-increased inflammatory-associated iNOS activity causes S-nitrosylation of IRE1α (a key UPR regulator) and defective activity.^309^

#### Sulfhydration in obesity

Sulfhydration is a PTM of cysteine residues (RSH) to persulfides (RSSH) and is involved in H_2_S-based signal transduction.

H_2_S is a novel factor mediating obesity and associated metabolic diseases. Plasma H_2_S levels were proved to be reduced in overweight participants.^310^ Cystathionine g-lyase (CSE), cystathionine b-synthase (CBS), 3-mercaptopyruvate sulfurtransferase (3-MST) and selenium-binding-protein 1 (SELENBP1) are four principal mammalian H_2_S-generating enzymes. CSE is the most critical H_2_S-generating enzyme. The deletion of CSE in adipocytes can reduce adipose accumulation and enhance lipolysis by abolishing sulfhydration of plin-1.^311^ CSE, CBS, and 3-MST mRNA were reduced in the WAT of *db/db* mice, while only CSE was reduced in BAT.^312^

In differentiated human adipocytes, sulfhydration was increased in proteins participating in fatty acid metabolism and other metabolic signaling pathways.

#### ADP-ribosylation in obesity

ADP ribosylation was mediated by PARP family of enzymes. Physiological ADP ribosylation of histone H2B-Glu inhibits AMPK-mediated phosphorylation of adjacent H2B-Ser36, which is required for proadipogenic gene expression and fat metabolism programs.^313^

#### Carbonylation in obesity

During the development of obesity, levels of ROS in adipocytes are increased. These ROS products covalently modify the histidine, cysteine and lysine residues via protein carbonylation. Protein carbonylation levels are elevated threefold in the adipose tissue of diet-induced obese mice and obese human individuals.

Increased protein carbonylation has been linked to the antioxidant enzyme GSTA4 and the fatty acid-binding proteins. GSTA4 is decreased in obese mice and humans. Carbonylation of FABP4 on Cys-117 results in loss of fatty acid binding activity in mice. Treatment of TNF-α in 3T3-L1 adipocytes can decrease GSTA4 expression and increase protein carbonylation similar to obese states.^314^ GSTA4-null or accumulated ROS in obese C57BL/6J mice results in impaired glucose and lipid homeostasis.^315^

The accumulation of ROS is linked to adipose oxidative stress in the obese state. Extensive evidence has demonstrated that the accumulation of 4-hydroxynonenal (HNE) acts as a symbol of oxidative stress, which usually occurs in the blood and tissue of obese/diabetic patients.

#### S-sulfenylation in obesity

Brown and beige adipose tissues need uncoupling protein 1 (UCP1) to execute thermogenesis and Cys253 of UCP1 is sulfenylated during thermogenesis, which provides the strategy to improve therapeutic strategies for combating metabolic disorders.^316,317^

### PTMs in fatty liver diseases

**Fig. 5:**
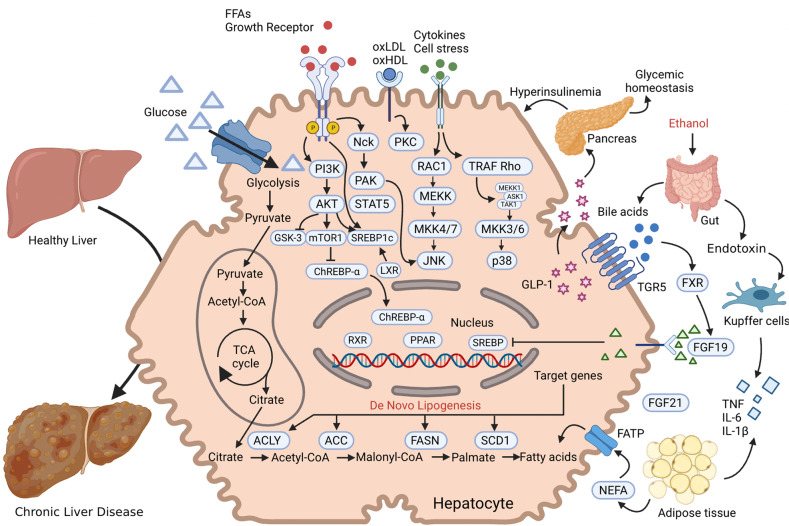
Overview of the roles of PTM in fatty liver diseases. Non-alcoholic fatty liver disease (NAFLD) is characterized by fat accumulation in the liver. We summarize the diverse roles of PTMs in the pathogenesis of NAFLD to enrich the understanding of the molecular mechanisms of the intricate interrelationship between post-translational modification (PTM) of important factors and fatty liver disease. (**1**) Multiple endogenous and exogenous stimuli can lead to aberrant dynamic proteins posttranslational modifications to result in chronic liver disease. (**2**) Many proteins have multiple modification sites to modulate lipid synthesis, lipolysis, and fatty acid oxidation by phosphorylation, acetylation or SUMOylation of key substrates, such as ACC, SREBPs, GPAT and FXR. Activation and inhibition effects are displayed in “arrows” and “inhibitors”, respectively. The figure is generated with BioRender (https://biorender.com). AKT protein kinase B, ACLY ATP citrate lyase, ACC acetyl CoA carboxylase, ChREBP carbohydrate response element binding protein, FFAs free fatty acids, FASN fatty acid synthase, FXR farnesoid X receptor, FGF19 fibroblast growth factor 19, FATP fatty acid transport protein, GSK-3 glycogen synthase kinase 3, JNK c-Jun N-terminal kinase, MEKK mitogen-activated extracellular signal-regulated kinase kinase, Nck non-catalytic region of tyrosine kinase adaptor protein, NEFA non-esterified fatty acids, PAK p21-activated kinase, SREBP1 sterol-regulatory element binding protein 1, STAT5 signal transducer and activator of transcription 5, SCD1 stearoyl-CoA desaturase 1, TRAF TNF receptor associated factors

Various novel signaling pathways in fatty liver have been identified. Many proteins have multiple modification sites to modulate lipid synthesis, lipolysis, and fatty acid β-oxidation by phosphorylation, acetylation or SUMOylation of key substrates, such as ACC, SREBPs, GPAT, and farnesoid X receptor (FXR). Current efforts are concentrated on exploring the molecular mechanism of the interrelationship between fatty liver disease and PTMs of important factors. Here, we summarize the different effects of PTMs in the pathogenesis of NAFLD (Fig. 5).

#### Phosphorylation in fatty liver disease

Protein kinases and phosphatases dynamically regulate protein phosphorylation. Protein kinases (PKs) families contain MAPK, ErbB, PKA-PKD, PI3K/Akt, and mTOR, acting on multiple downstream key protein targets in NAFLD and regulating hepatic gluconeogenesis, lipogenesis and inflammation. AMPK has multiple phosphorylation sites to modulate lipid synthesis, lipolysis, and fatty acid oxidation by phosphorylation of ACC, SREBPs, GPAT and so on. ACC can catalyze the carboxylation of acetyl-CoA to malonyl-CoA, which acts as an important regulatory site of fatty acid synthesis and oxidation pathways. The Ser79, Ser1200 and Ser1215 sites of ACC can be phosphorylated by AMPK. SREBPs directly promote genes involved in fatty acid uptake and triglyceride (TG) synthesis by phosphorylation. Sn-Glycerol-3-phosphate acyltransferase (GPAT) is the rate-determining enzyme that catalyzes TG synthesis. HSL catalyzes the rate-limiting step in TG hydrolysis. AMPK inhibits HSL by phosphorylating its Ser660 and Ser563 sites, thus suppressing lipolysis in adipocytes.^318^

Mitogen-activated protein kinases (MAPKs) include stress-responsive MAPKs, JNK, and p38 MAPK. and ERK1/2. JNK can be phosphorylated by MAPKK and MAPKKK. Lipid accumulation can enhance hepatic JNK, which contributes to liver injury.^319^ JNK-1 knockout mice showed reduced liver steatosis and TG accumulation.^320^ The activation of p38 MAPK promotes the expression of PPARα, CPT1A, and PGC-1α to suppress hepatic fat accumulation. PKC is increased accompanied by high hepatic lipid content in obese mouse models. PKC can directly phosphorylate IRS to reduce downstream insulin signaling.^321^ Akt signaling is triggered by activated-PI3K and requires phosphorylation of Thr308 and Ser473 via PDK-1 and mTORC2. Activated AKT-1 promotes the levels of transcription factors involved in DNL and increases the intracellular lipid content.^322^ High-fat diets can decrease the ratio of p-AKT/AKTt but increase the expression of SREBP1, LXR, ChREBP, ACC1, and fatty acid synthase (FASN).^323^

#### Acetylation in fatty liver disease

Proteomic analyses have identified a large number of acetylated proteins involved in intermediate metabolism. Reversible acetylation is controlled by acetyltransferases (KATs) and deacetylases (HDACs and SIRTs). Protein acetylation can regulate metabolism in chronic liver diseases. Hyperacetylated-LDHB has been detected in NAFL and NASH human samples.^324^

The zinc finger protein Snail1 recruits HDAC1/2 to induce the deacetylation of H3K9 and H3K27 to repress lipogenesis.^325^ HDAC3 controls the circadian rhythm of hepatic lipogenesis.^326^

SIRT1, an NAD^+^-dependent protein deacetylase, regulates lipid homeostasis by positively regulation of peroxisome proliferators-activated receptor α (PPARα). Exenatide (exendin-4) can improve hepatic steatosis via the SIRT1/heat shock factor 1/HSP pathway.^327^ SIRT2 regulated hepatic steatosis by HNF4α deacetylation.^328^ A natural chemical compound 2,3,5,4’-tetrahydroxy-stilbene-2-O-β-d-glucoside (TSG) reduces ROS formation and increases SIRT5 expression in mitochondria to ameliorate NAFLD.^329^ USP10 inhibits hepatic steatosis and inflammation by interacting with SIRT6.^329^ In mitochondria, MRG15 interacts with and deacetylates TUFM, which accelerates effects altogether and drives the progression from NAFLD to NASH with inflammation and fibrosis.^330^

#### Methylation in fatty liver disease

Aberrant histone methylation also takes participate in the process of CLD. Downregulation of glycine N-methyltransferase (Gnmt) occurred in the early stage of pathogenesis of NAFLD and promotes the development of NAFLD-derived HCC. Histone 3 lysine 9 methyltransferase enzyme (G9a) is downregulated in diet-induced animal models of obesity. Transgenerational HFD feeding reduces the accumulation of H3K9 histone methyltransferase in the LXRα and ERO1-α gene promoters and activates ChREBP-mediated both glycolytic and fatty acid synthesis.^331^

Histone demethylase Jumonji domain-containing protein 2B (JMJD2B) removes histone marks (H3K9me2 and H3K9me3) near the LXR response elements (LXREs) to play a role in liver X receptor α (LXRα)-mediated lipogenesis and contribute to hepatic steatosis. In addition, histone demethylase plant homeodomain finger 2 (Phf2) regulates H3K9me2 demethylation at carbohydrate-responsive element binding protein (ChREBP) to prevent NAFLD progression.^332^

#### Ubiquitination in fatty liver disease

Ubiquitination is an important PTM to cope with abnormally folded or damaged proteins which exert diverse functions in chronic liver disease.

E3 Ub ligases and DUBs play important roles in protein ubiquitination and deubiquitination. The activation of E3 ubiquitin ligase Ubr1, inducing polyubiquitination of Plin2 to prevent steatosis in mouse livers.^333^ E3 ubiquitin ligase-tripartite motif-containing protein 31 (TRIM31) promotes degradation of Rhbdf2 by K48-linked polyubiquitination to alleviate NAFLD in mouse hepatocytes.^334^ Knockout of Kindlin-2 destroys the stability of Foxo1 by promoting its ubiquitination and degradation through Skp2 E3 ligase-dependent ubiquitination, which can protect against fatty liver.^335^ Ring finger protein 5 (RNF5) can directly combine with HRD1 and promote ubiquitination and degradation to inhibit NASH progression.^336^ Moreover, sorting nexin 8 (SNX8) promoted FASN protein proteasomal degradation and protected NAFLD by recruiting the E3 ligase tripartite motif containing 28 (TRIM28).^337^ The E3 Ub ligases TRIM8 and TRIM16 can directly bind to TAK1 to promote its phosphorylation and activate JNK/p53 and NF-κB signaling. TRIM8 and TRIM16 can mitigate hepatic steatosis and fibrogenesis in NASH.^338^ The E3 ligase FBXW5 mediates ASK1 ubiquitination and exacerbates NASH.^339^ Liver and adipocytic MKRN1 is an E3 ubiquitin ligase for AMPK. MKRN1-null mice can suppress diet-induced metabolic syndrome.^340^

DUBs catalyze the removal of ubiquitin from protein substrates in the process of ubiquitination. Hepatic USP4 is directly bound to deubiquitinated TAK1, leading to amelioration of metabolic dysfunction.^341^ The USP7/ZNF638 axis mediates de novo lipogenesis.^342^ Ubiquitin-specific peptidase 10 (USP10) decreases over time in patients with NAFLD and in HFD-fed mice. USP10 can interact with Sirt6 and inhibit its ubiquitination and degradation to inhibit hepatic steatosis and inflammation.^343^ USP14 expression has been revised upwards in the livers of HFD, *db/db* mice and NAFLD patients and plays an indispensable role in hepatosteatosis via stabilization of FASN.^344^ Moreover, USP14 deubiquitinates HIF1-α to maintain its stability in hepatocellular carcinoma.^345^ The deubiquitinase cylindromatosis (CYLD) interacts with TAK1 and removes its K63-linked polyubiquitin chain to mitigate NASH.^346^

#### SUMOylation in fatty liver disease

An increasing body of evidence suggests that SUMOylation is closely associated with the progression of liver diseases. For example, UBC9 is the only known E2-conjugating enzyme involved in SUMOylation to regulate hepatic fibrosis. The SUMO-1-conjugating enzyme UBC9 reduces the transcriptional activity of two sumoylation sites in SREBP-1a to inhibit lipid production.^347^ In addition, small ubiquitin-related modifier (SUMO) E3 ligase sumoylates SREBP1c at Lys98, reinforcing the interaction between SREBP1c and PIASγ, which can regulate hepatic lipid metabolism during nutritional deprivation.^348^

The de-SUMOylation enzyme SENP2 decreased CCl4-induced mouse fibrosis in liver tissues. Last but not least, liver receptor homolog 1 (LRH-1) is an important regulator of hepatic metabolism. The SUMOylation-defective mutant of LRH-1 mice developed NAFLD and early symptoms of NASH under the regulation of OSBPL3 when fed a high-fat and high-sucrose diet.^349^

#### Neddylation in fatty liver disease

Recently, numerous mechanistic studies have been performed to elucidate the crucial role of neddylation in lipid metabolism. SRSF3 degradation via lysin11 neddylation partially protects mice from NAFLD and deletion of SRSF3 predisposes to hepatocellular carcinoma in mice.^350^ In addition, dysregulation of NRF2 by neddylation of cullin 3 was connected with AGER1 downregulation and NASH aggravation.^351^ It has been demonstrated that neddylation inhibition in vivo in NAFLD pre-clinical models inhibits mTOR activation and induced protein DEP-domain containing mTOR-interacting protein (DEPTOR), thus mediating anti-steatotic effects as well as boosting hepatic fatty acid oxidation.^352^

More recently, neddylation was described for the first time in liver fibrosis and is found to be deregulated in patients with liver fibrosis and CCl_4_-induced fibrosis mice.^353^ Preventing the neddylation-dependent degradation of serine-rich splicing factor 3 (SRSF3) protected mice from hepatic steatosis, fibrosis and inflammation.^350^ The neddylation modification of TGFβ-RII plays a critical role in HSC activation.^353,354^

The components of the neddylation pathway may become novel biomarkers for CLD diagnosis.

#### Glycosylation in fatty liver disease

Recent studies have indicated that many proteins involved in glycosylation play a part in the pathogenesis of NAFLD.

Recently, glycosyltransferase 8 domain containing 2 (Glt8D2) expression was found to be increased in patients with severe NAFLD.^355^

O-GlcNAcylation involving in liver metabolism by suppressing insulin signaling and activating lipogenic pathways. Protein O-GlcNAcylation was increased in NAFLD and NASH mice during lipid accumulation. Furthermore, O-GlcNAcylation can also modify the ChREBP and FXR in the liver.^356^ ChREBP is a key regulator of glycolysis and lipogenesis. O-GlcNAcylation can increase the ChREBP protein level.^357^

#### Palmitoylation in fatty liver disease

The latest research detected the upregulated palmitoylation of FAT/CD36 in NAFLD. Suppressed FAT/CD36 palmitoylation promotes FAT/CD36 localization change and avoids lipid accumulation in NAFLD.^358^

#### Prenylation in fatty liver disease

Protein prenylation includes protein farnesylation and geranylgeranylation. Abnormal expression of geranylgeranyl diphosphate synthase (GGPPS) breaking the balance of protein farnesylation and geranylgeranylation. The high expression of GGPPS was detected in the livers of NAFLD patients.^359^

#### Glutathionylation in fatty liver disease

Glutathione S-transferase π (GSTπ) is shown to promote S-glutathionylation. The decreased expression of GSTπ reduces protein S-glutathionylation and prevents hepatic lipid accumulation during liver development.^360^

#### S-nitrosylation in fatty liver disease

S-nitrosylation (SNO) can promote the conversion of NAFLD to NASH via the peroxisome PPARγ/SFRP5 pathway.^361^

#### Sulfhydration in fatty liver disease

S-sulfhydration forms a hydro persulfide moiety (-SSH) or polysulfide in the active cysteine residues. According to a prediction, one-third of proteins could be modified forming S-sulfhydration products. In a previous study, H_2_S showed a protective effect in HFD-induced NAFLD or CDA-induced NASH. CSE plays an important role in the methionine trans-sulfuration pathway. CSE/H_2_S promoted a protein sulfhydration of FXR at Cys138/141 sites to promote FXR activity and attenuate NAFLD. Deficiency of CSE in the liver promotes the pathological process of nonalcoholic steatohepatitis.^362^ Cystathionase (CTH) is another metabolic enzyme to mediate the synthesis of H_2_S in the liver. SREBF1/SREBP-1c was activated under an HFD-induced liver steatosis model. The upregulated Mir216a transcription directly decreased CTH-H_2_S sulfhydration signaling and ULK1-stimulated autophagy to promote hepatic steatosis.^363^

Strikingly, S-sulfhydration of Keap1 was decreased in the liver of NAFLD patients. H_2_S plays a protective role depending on the S-sulfhydration of Keap1 to alleviate liver damage through enhanced Nrf2-mediated antioxidant responses.^364^

#### ADP ribosylation in fatty liver disease

PARP1 is activated in the liver of HFD-fed mice and suppressed PPARα signaling.^365^ A PARP inhibitor olaparib reversed NAFLD by NAD^+^ elevation, increasing mitochondrial biogenesis and β-oxidation in liver under HFHS diet.^366^

#### Carbonylation in fatty liver disease

Protein carbonylation is associated with metabolic effects. Examining fatty nonalcoholic steatohepatitis carbonylated proteins by functional enrichment analysis, increased carbonylation was evident in proteins regulating Rho cytoskeletal pathways, nicotinic acetylcholine receptor signaling and chemokine/cytokine inflammatory pathways.^367^ Disruption of PTEN resulted in steatohepatitis and fibrosis in mice, but elevated Nrf-2 responses are not enough to relieve protein carbonylation in hepatocyte-specific PTEN deficient mice.^368^

### PTMs and their roles in other metabolic conditions and diseases

#### PTMs in hyperlipidemia

Hyperlipidemia is a state of elevated levels of fats and lipids in the blood (TGs, cholesterol, or both), encompassing numerous genetic and acquired disorders. TG is the most popular and effective type of energy storage in animal tissues and originates from both dietary intake and endogenous (liver) generation.^369^ Cholesterol is the principal sterol in mammals and is mainly derived from dietary sources and partly endogenous synthesis by the liver and other tissues.^369^ Briefly, a range of apolipoproteins package TGs and cholesterol into lipoproteins, which are transported in vessels. Plasma lipoproteins are grouped into chylomicrons (CM), very low-density lipoprotein (VLDL), low-density lipoprotein (LDL), and high-density lipoproteins (HDL) based on size, density and apolipoprotein content.^370^

Exogenous TGs are converted to glycerol and fatty acids; the fatty acids are employed for energy metabolism (β oxidation of fatty acids) or storage. Extra energy is stored in the liver and adipose tissues as triglycerides via the fatty acid pathway.^370^ Fatty acids can also be produced from carbon sources undergoing several enzymatic processes known as de novo lipogenesis (DNL). Surplus cellular energy induces an increase in mitochondrial citrate, initiating DNL. Mitochondrial citrate travels across the plasma membrane with citrate/isocitrate carrier (CIC) and triggers synthesis through ATP-citrate lyase (ACLY), acetyl-CoA carboxylase (ACC), FASN, and downstream lipid processing with stearoyl-CoA desaturase 1 (SCD1) and DGAT.^371^ SREBPs, LXRs, and ChREBPs mediate the transcription of CIC, ACLY, ACC, and FASN.^372^ Liver-derived lipoproteins travel through the bloodstream carrying endogenous triglycerides for uptake by peripheral tissues.

Dietary cholesterol is absorbed in the intestine, packaged and released as chylomicrons, and finally returns to the liver. Cholesterol biosynthesis originates from acetyl-CoA and requires more than 20 enzymes. Cholesterol biosynthesis mainly localizes in ER, where the liver is the leading site of biosynthesis. 3-Hydroxy-3-methylglutaryl coenzyme A reductase (HMGCR) and squalene monooxygenase (SM) are two rate-limiting enzymes, and SREBP2 is the master transcriptional regulator. Livers deliver endogenously produced and exogenously absorbed cholesterol to the blood with VLDL, after which VLDL gradually changes into circulating LDL for intake by peripheral tissues. HDL transports peripheral cholesterol back to the liver and intestine during the reverse cholesterol transport (RCT) process. Cholesterol participates in bile acids synthesis in the liver or contributes to steroid hormone synthesis. Excess cholesterol is esterified through acyl-coenzyme A:cholesterol acyltransferase (ACAT) for storage or release with lipoproteins.^373^

**Fig. 6:**
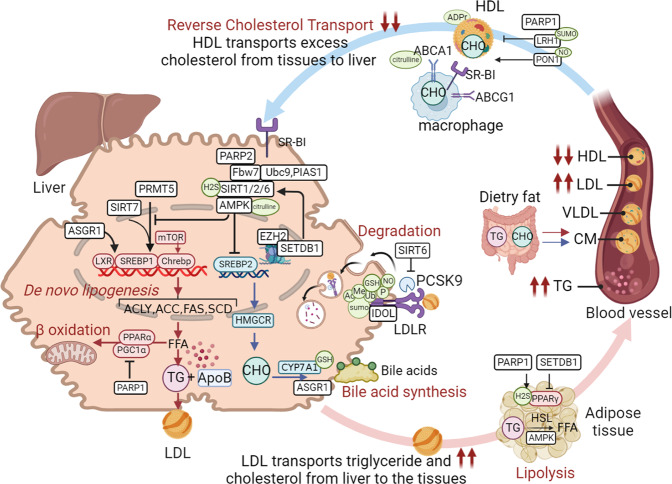
Diverse roles of PTMs in hyperlipidemia. Excessive intake and synthesis or insufficient catabolism of lipids lead to hyperlipidemia. De novo lipogenesis (DNL), reverse cholesterol transport (RCT), fatty acid β-oxidation, lipolysis, bile acid synthesis, and lipoprotein-related-regulation contribute to lipid homeostasis at multiple levels. We summarise the role of crosstalk among PTMs in regulating lipid metabolism. (**1**) During hepatic DNL, abundant PTMs such as phosphorylation (AMPK), (de)acetylation (SIRT1/2/6), methylation (EZH2, PRMT5), ubiquitination (FBW7, ASGR1), SUMOylation (SUMO1, SENP1, UBC9, PIAS1), sulfhydration (NsHS/H_2_S), citrullination (L-citrulline-AMPK) and ADP ribosylation (PARP2) can regulate the expression of ACLY, ACC, FAS, SCD via transcription factor mTOR, LXR, SREBP and CHREBP. (**2**) Fatty acid beta-oxidation. ADP ribosylation (PARP1) inhibits beta-oxidation via PPARα. (**3**) Lipolysis. LDL transports fatty acid from the liver to peripheral tissues. In adipose tissues, acetylation (SETDB1), ADP ribosylation (PARP1), sulfhydration (H_2_S) and phosphorylation (AMPK) modulate the lipolysis by regulating key enzyme HSL or transcription factor PPARγ. (**4**) Reverse cholesterol transport (RCT). SUMOylation (SUMO-LRH1), S-nitrosylation (NO-PON1-HDL), citrullination and ADP ribosylation regulate RCT by modifying HDL or relevant enzymes. (**5**) Bile acid transport. Ubiquitination (ASGR1) and glutathionylation (GSH) govern cholesterol excretion by promoting bile acid synthesis. (**6**) LDLR. Hepatic LDLR mediates LDL uptake and the subsequent degradation, abolishing excess serum LDL, which is inhibited by PCSK9-mediated LDLR degradation. Several PTMs such as phosphorylation acetylation, methylation, S-nitrosylation, ubiquitination, SUMOylation and glutathionylation could regulate LDLR functioning. Acetylation and ubiquitination modulate LDLR function via the SIRT6-PCSK9-LDLR axis and LXR-IDOL-LDLR axis. The red arrows indicate the triglyceride (TG) pathway, the blue arrows indicate the cholesterol (CHO) pathway, and the black arrows indicate the roles of PTM-related molecules in hyperlipidemia. The figure is generated with BioRender (https://biorender.com). ABCA1 ATP binding cassette transporter, ABCG1 ATP binding cassette transporter, ACC acetyl-CoA carboxylase, ACLY ATP-citrate lyase, AMPK AMP-activated protein kinase, ASGR1 asialoglycoprotein receptor 1, Apo apolipoprotein, CM chylomicrons, CYP7A1 cholesterol 7α-hydroxylase 1, ChREBP carbohydrate-responsive element-binding protein, EZH2 enhancer of zeste homolog 2, FAS fatty acid synthase, FFA fatty acid, GSH glutathione, H_2_S hydrogen sulfide, HDL high-density lipoprotein, HMGCR 3-Hydroxy-3-methylglutaryl coenzyme A reductase, HSL hormone-sensitive lipase, IDOL inducible degrader of LDLR, LDL low-density lipoprotein, LRH-1 liver receptor homolog-1, LXR liver X receptor, PARP poly (ADP-ribose) polymerase, PGC1α PPARγ coactivator 1α, PIAS1 protein inhibitor of activated STAT 1, PPAR peroxisome proliferator-activated receptor, PRMT protein arginine methyltransferase, SCD stearoyl-CoA desaturase, SENP1 sentrin/SUMO-specific protease 1, SETDB1 SUMOylated SET domain bifurcated 1, SIRT sirtuin, SR-BI scavenger receptor class B member 1, SREBP sterol regulatory element-binding protein, VLDL very low-density lipoprotein, mTOR mammalian target of rapamycin complex, PTMs post translational modifications

Excess intake and synthesis or insufficient catabolism of lipids will lead to hyperlipidemia. The metabolism of lipids is intricately regulated by various molecules and enzymes, involving abundant posttranslational modifications. As shown in Fig. 6, de novo lipogenesis, β-oxidation, cholesterol reverse transport, bile acid synthesis and lipoprotein transport are involved in lipid metabolism. Various PTMs play vital roles in regulating lipid homeostasis through diverse mechanisms. There is an incomplete understanding of PTMs in regulating lipid metabolism, and we thus will highlight the roles of common PTMs in lipid homeostasis and hyperlipidemia.

##### Phosphorylation in hyperlipidemia

Protein phosphorylation contributes to lipid metabolism by regulating critical substrates in lipolysis, lipid biosynthesis, and fatty acid β oxidation. AMPK, an energy-regulating kinase, regulates several critical molecules involved in lipid metabolism, such as ACC, SREBP, and some key enzymes.

ACC is a vital site in fatty acid biosynthesis and oxidation pathways with ACC1 and ACC2 isoforms. ACC1 mediates the process through which acetyl-CoA converses into malonyl-CoA in the synthesis pathway, and ACC2 suppresses carnitine palmitoyltransferase 1 (CPT1) to decrease β-oxidation. AMPK phosphorylates ACC1 at Ser79, leading to the inactivation of ACC1 and reduction in fatty acid synthesis.^18^ AMPK phosphorylates ACC2 at Ser219, resulting in the inhibition of ACC2 and increased fatty acid oxidation.^374^

SREBPs include three isoforms, SREBP1a, SREBP1c, and SREBP2, promoting related gene expression in lipid synthesis. AMPK phosphorylates SREBP1c at Thr426, Ser410, Ser430, or Ser372, inhibiting the expression of SREBP1c and suppressing TG synthesis.^375^ AMPK can also inhibit SREBP1c by reducing the transcription of the mTORC^376^ and the subsequent expression of FASN. AMPK can directly restrain SREBP2 by phosphorylation, repress HMGCR and cholesterol synthesis, and ameliorate dyslipidemia.^375^

Hormone-sensitive lipase (HSL) is the key rate-limiting enzyme catalyzing TG hydrolysis. AMPK agonist AICAR can suppress HSL activity by phosphorylation at Ser565 and inhibit lipolysis in adipocytes.^318^

HMGCR is a crucial rate-limiting enzyme in cholesterol synthesis. The balance between phosphorylation and dephosphorylation is suggested to influence HMGCR activity. AMPK inactivates HMGCR by phosphorylating Thr172, but AMPK activates HMGCR by phosphorylating Ser872 and promotes hypercholesterolemia.^377,378^

The role of protein kinases in lipid metabolism is widely known. Further exploration of novel therapeutic strategies targeting protein phosphorylation and protein kinases is essential for designing novel lipid-lowering therapies.

##### Acetylation in hyperlipidemia

Acetylation can markedly alter protein function through changes in hydrophobicity, solubility, and surface properties. Sirtuins (class III HDACs) are a group of remarkably conserved NAD^+^-dependent deacetylases catalyzing deacetylating reactions in crucial proteins related to lipid metabolism. Sirtuins are suggested to restrain lipogenesis and prevent lipid accumulation by promoting fatty acid beta-oxidation or exporting surplus lipids.

SIRT1 can deacetylate SREBP1c and cause SREBP1c ubiquitination and degradation, suppressing fatty acid and cholesterol synthesis.^379^ SIRT1 induces PPARα activation and enhances fatty acid beta-oxidation.^380^ SIRT2 is suggested to deacetylate ACLY in the liver^381^ and suppress PPARγ coactivator 1α (Pgc1α) in adipose tissue^382^ to inhibit lipid accumulation. SIRT6, regulated by SIRT1, promotes the acetylation of histone H3 lysine 9 to inhibit TG synthesis.^27^ Hepatic SIRT6 can inhibit PCSK9 transcription, preventing LDLR breakdown and thereby lowering plasma LDL levels in mice.^383^ Large yellow tea extract lowers blood lipids in leptin receptor knockout mice by inhibiting lipogenesis via activating the SIRT/SREBP pathway.^384^

The discovery of novel mechanisms of deacetylases regulating lipid homeostasis is an active field of exploration. Further identification of novel agents targeting protein acetylation and the related enzymes is warranted.

##### Methylation in hyperlipidemia

Protein methylation is a contributor to numerous human diseases. Methyltransferase (lysine methyltransferase, arginine methyltransferase) and demethyltransferase are the enzymes that regulate protein methylation.

Lysine methyltransferase is involved in lipid metabolism. LncRNA PU.1 AS can reduce plasma TG and hepatic TG by interacting with EZH2 (a histone-lysine N-methyltransferase) and decreasing lipogenesis via the EZH2/SIRT6/SREBP1c pathway.^385^

Arginine methyltransferase can also regulate lipid metabolism. Butyrylcholinesterase (BChE) interacts and colocalizes with PRMT5, regulating LDLR-mediated LDL intake via the MEK-ERK pathway. BChE-deficient mice exhibit PRMT5 degradation and are susceptible to hypercholesterolemia.^34^ PRMT5 can mediate symmetric arginine demethylation. PRMT5 has been demonstrated to promote SREBP1 SDM, activating enzymes regulating cholesterol synthesis^386^ and fatty acid synthesis.^387^

A better understanding of protein methylation in lipid metabolism is required to support the development of medicines for regulating lipid profiles.

##### Ubiquitination in hyperlipidemia

Multiple factors, such as transcription, translation and enzymatic activity, are involved in lipid homeostasis. Recently, evidence of the role of ubiquitin ligases in lipid metabolism has been provided. Here, we will describe the role of E3 ubiquitin ligases.

IDOL, the individual E3 ubiquitin ligase, regulates LDLR degradation to induce hypercholesterolemia through the LXR-IDOL-LDLR axis.^388,389^ IDOL regulates circulating lipid metabolism and the development of atherosclerosis independent of LDLR function.^389^ A few E3 ligases regulate SREBP. ITCH can degrade SIRT6 via ubiquitination to promote fatty acid beta-oxidation. ITCH deficiency can disturb nuclear SREBP clearance and decrease circulating cholesterol levels.^390^ FBW7, a member of the SKP1-cullin-1-F-box (SCF) complex ubiquitin ligases, regulates SREBP degradation, which is negatively mediated by microRNA-182.^391^ E3 ligase TRIM72/MG53 regulates the ubiquitin-dependent degradation of IRS1 and insulin receptors. TRIM72/MG53 deficiency would alleviate insulin resistance and hyperlipidemia in HFD-mice, while TRIM72/MG53 overexpression would aggravate insulin resistance and hyperlipidemia.^295^

Some E3 ligases play a role in cholesterol metabolism. Human TEB4, an E3 ligase resident in the ER membrane, degrades squalene monooxygenase (SM) to inhibit cholesterol synthesis.^392^ Glycoprotein 78 (gp78) can enhance the ubiquitinated degradation of HMGCR,^393^ insulin-induced gene 1 protein (INSIG1)^394^ and ApoB-100.^395^ Hepatic gp78 deficiency alleviates hyperlipidemia and insulin resistance through reducing lipid biosynthesis.

Recently, Song and colleagues found that inhibition of asialoglycoprotein receptor 1 (ASGR1) would upregulate ABCA1, ABCG5/ABCG8, LXRα, suppress SREBP and lipogenesis, subsequently facilitate cholesterol excretion and alleviate hyperlipidemia.^45^ Mechanistically, ASGR1 deficiency decreased LXRα ubiquitination, whereas overexpression of ASGR1 increased its ubiquitination.^45^

The role of protein ubiquitination of the UPS system in lipid metabolism is recognized. Further investigation of novel therapeutic approaches regulating E3 ligases and the UPS system may hold promise for the treatment of hyperlipidemia.

##### SUMOylation in hyperlipidemia

SUMOylation, a SUMO-mediated protein modification, influences many cellular processes by affecting protein stability, activity, interactions and cellular localization; accordingly, SUMOylation has been identified to participate in lipid homeostasis.

SUMO regulates cholesterol homeostasis involving cholesterol synthesis, ingestion, transport and bile acid metabolism. SUMOylated LRH-1 recruits corepressor prospero-related homeobox protein 1 (PROX1) and suppresses LRH-1-dependent genes associated with RCT.^396^ FXR, a bile-acid-stimulated nuclear receptor, restrains the biosynthesis and transport of bile acid through small heterodimer partner (SHP) and LRH-1. SUMO1 decreases the attachment of FXR to the promoter of SHP, inhibiting bile acid synthesis and transport.^397^ UBC9, the E2 SUMO-conjugating enzyme, can interact with SUMOylate SREBP2 at the lysine463 site, suppressing SREBP2 transcriptional activity and repressing cholesterol synthesis.^347^ Protein inhibitor of activated STAT 1 (PIAS1), with SUMO E3 ligase activity, inhibits LXR-dependent fatty acid synthesis and lipogenic genes, including *Srebp1* and *Fasn*.^398^

SENP, mediating deSUMOylation, can also regulate lipid metabolism. SENP1 can raise LDLR expression through deSUMOylation.^56^ SUMOylated SET domain bifurcated 1 (SETDB1) inhibits the expression of *Pparg* and *Cebpa* (CCAAT Enhancer Binding Protein Alpha). SENP2 deficiency induces the accumulation of SUMOylated SETDB1, repressing the expression of *Pparg* and *Cebpa,* thus decreasing lipid storage in adipocytes.^399^

Further elucidation of protein SUMOylation may yield novel therapeutic targets for the treatment of hyperlipidemia.

##### Glycosylation in hyperlipidemia

Glycosylation of human lipoproteins exhibits high diversity, playing crucial roles in regulating lipoprotein metabolism.

N-glycosylation regulates proteins involved in lipid synthesis, package, and the abolition of lipoproteins. N-glycosylation in humans reduces LDL through enhanced LDLR expression.^72^ Glucose-induced N-glycosylation maintains SREBP cleavage-activating protein (SCAP) stability and decreases its interaction with INSIG1, permitting SREBP activation and downstream gene transcription.^400^ *SCARB1* codes scavenger receptor class B member 1 (SR-BI), the primary receptor in selective HDL uptake into the liver. A loss-of-function genetic variant in *SCARB1* (leucine replaces proline 376) induces altered N-glycosylation, increasing HDL-cholesteryl ester and preventing its uptake into hepatocytes due to decreased SR-BI.^401^

O-glycosylation links GlcNAc and GalNAc to target proteins. Site-specific glycan profiles of HDL-related ApoE are closely involved in HDL activity and function.^402^ The galNAc-T2 enzyme (encoded by *GALNT2*) catalyzes the GalNAc linkage in O-glycosylation. The *GALNT2* variant has been shown to affect HDL and triglyceride levels in human genetics studies.^403^ However, reduced GalNAc-T2 function was related to increased HDL.^404^ Others demonstrated that GalNAc-T2 function defect reduced plasma HDL,^405^ which made the role of O-glycosylation in HDL metabolism somewhat mysterious. However, *GALNT2* has been shown by glycoproteomics to serve as the inducer of phospholipid transfer protein (PLTP) and is required in maintaining HDL levels in mammals.^405^

A clearer understanding of how lipoproteins are regulated by protein glycosylation might provide new therapeutic insights into the treatment of dyslipidemia and its clinical consequences.

##### Lipidation (palmitoylation and myristoylation) in hyperlipidemia

Protein lipidation is an essential mechanism allowing proteins to shuttle between organelles and the membrane to alter structure, activity and function.

Palmitate, a sixteen-carbon saturated fatty acid, and can be linked to cysteine residues by thioester bonds. The rapid shift of palmitoylation and depalmitoylation reversibly regulates protein trafficking and function. ELMO domain containing 2 (ELMOD2), a nonclassical ADP-ribosylation factor (Arf)-GTPase activating protein, can handle the transport of adipocyte triglyceride lipase (ATGL) to lipid droplets. Through palmitoylation, ELMOD2 is bound to lipid droplets and regulates ATGL recruitment.^80^

Myristic acid, a fourteen-carbon saturated fatty acid, typically and irreversibly binds to the glycine residue by a covalent bond through myristoylation. In a study, adenovirus-mediated overexpression of hepatic NH(2)-terminal myristoylated signal-attached Akt (myr-Akt) leads to hypertriglyceridemia, hypoglycemia and hypoinsulinemia, which is regulated by Akt-mediated SREBP1 expression and fatty acid biosynthesis in an SREBP1-independent manner.^88^

Evidence of lipidation in hyperlipidemia is sparse, thus further exploration of lipidation in lipid metabolism might provide us with new perspectives on regulating dyslipidemia.

##### Glutathionylation in hyperlipidemia

S-glutathionylation is a vital redox regulatory mechanism involving the attachment of oxidized glutathione and protein thiol through a mixed disulfide linkage. In redox regulation, GSH undergoes S-glutathionylation and is then reversed via enzymatic or chemical reduction. Glutathione S-transferase and peroxiredoxins promote protein S-glutathionylation, while glutaredoxins (Glrx) mainly reverse it. S-glutathionylation and related enzymes play crucial roles in dyslipidemia.

S-glutathionylation can modify lipid metabolism-related enzymes and factors. Paraoxonase 1 (PON1) is an esterase related to HDL in serum that mediates macrophage cholesterol efflux. Oxidative stress can induce S-glutathionylation in PON1 and subsequent reversible inactivation.^406^ Elevated glutathionyl haemoglobin level is found in patients with hyperlipidemia, suggesting the role of protein glutathionylation and oxidative stress.^110^

Glutaredoxins are widely involved in lipid metabolism. Glrx knockout mice on a chow diet exhibit hyperlipidemia, obesity, and fatty liver. Glrx supplementation inhibits hepatic lipid levels in a short time, suggesting that upregulation of Glrx could potentially improve lipid metabolism.^407^ One possible explanation might be that SITR1 inactivation induces S-glutathionylation and promotes hyperacetylation and hyperactivation of SREBP.

The normal functioning of protein glutathionylation is crucial, and it may be a potential target for lipid hemostasis regulation.

##### S-nitrosylation in hyperlipidemia

Nitrosylation is the covalent incorporation between the nitrosyl moiety of NO and target molecules. Nitrosylation occurs at the thiol group of cysteine, which is known as S-nitrosylation. There is only scarce evidence of protein S-nitrosylation in hyperlipidemia, mainly regarding lipoprotein regulation. S-nitrosylation leads to an interaction between NO and HDL-related PON1 at cysteine 284, abolishing PON1 enzymatic activity and resulting in the inactivation of lipid peroxides and cholesterol efflux disorders.^119^ The phosphotyrosine binding domain protein ARH is an adaptor protein interacting with LDLR and is required for well-organized LDLR activity. ARH requires S-nitrosylation via NO to allow LDL uptake by LDLR.^408^

Evidence of S-nitrosylation in hyperlipidemia is sparse, further exploration and a better understanding of S-nitrosylation and lipid homeostasis may offer new opportunities in dyslipidemia research.

##### Sulfhydration in hyperlipidemia

Sulfhydration alters the thiol group of cysteine residues to a persulfide (-SSH) group, leading to the enhanced reactivity of the cysteine residue. H_2_S is essential in lipid homeostasis through protein sulfhydration.

H_2_S treatment decreases plasma triglycerides by stimulating hepatic autophagy via AMPK-mTOR signaling.^409^ H_2_S donors (NaHS or GYY4137) directly activate SIRT1 by sulfhydration, restrain cholesterol uptake in macrophages and inhibit cholesterol biosynthesis in the liver, thereby reducing plasma lipid levels in *ApoE*^-/-^ mice.^410^ NaHS-derived H_2_S promotes HRD1 sulfhydration, thereby increasing the interaction between DGAT1, DGAT2 and HRD1, repressing lipid droplet development in the heart of *db/db* mice.^129^

Therefore, targeting H_2_S and related protein sulfhydration may contribute to novel strategies and targets for treating hyperlipidemia and its consequences.

##### Citrullination in hyperlipidemia

Citrullination is an irreversible chemical process converting arginine to citrulline; citrullination has an impact on protein denaturation, hydrogen bond generation, protein structure, charge and protein–protein interactions. To date, only limited studies have indicated that protein citrullination participates in lipid metabolism.

Citrulline could increase the expression of ATP-binding cassette transporter (ABCA1) and ATP-binding cassette subfamily G member 1 (ABCG1) in macrophages, thereby promoting reverse cholesterol transport.^411^ Treatment with L-citrulline promotes fatty acid β-oxidation and restrains hepatic fat accumulation by increasing AMPK phosphorylation.^138^

It is necessary to conduct more research to determine how citrullination regulates lipid metabolism.

##### ADP ribosylation in hyperlipidemia

ADP-ribosylation can be divided into MARylation and PARylation. PARP activity is modulated by a group of cholesterol-related compounds, including cholesterol derivatives, bile acids and steroid hormones. In turn, PARPs play a vital role in lipid homeostasis.

PARPs affect abundant lipid-activated nuclear receptors, including PPARγ, PPARα, and LXR. PARP1 inhibitors can reduce the expression of PPARγ-dependent molecules (including adiponectin and CD36) in the late adipogenesis stage in adipocytes.^412^ PARP1 could restrain PPARα transactivation directly via ADP-ribosylation, thereby reducing gene expression involving fatty acid β-oxidation in the liver.^380^ PARP1 can ADP-ribosylate LXR, regulating ABCA1-modulated cholesterol efflux in macrophages. PARP1 inhibitors increase cholesterol efflux and regulate lipid homeostasis via the LXR-ABCA1 axis.^413^ PARP7 is a mono-ADP-ribosylation polymerase that can also ADP-ribosylate LXR.^414^

PARPs can also influence lipid-related pathways such as fatty acid metabolism, cholesterol homeostasis and the regulation of lipoprotein. PARPs facilitate fatty acid biosynthesis and transport by stimulating the expression of related genes, including *FASN*, *CD36*, *FABP3*, *FABP4*, and *FABP7*.^415,416^ PRAP (PARP1, PARP2, PARP10) deficiency or inhibition promotes fatty acid β-oxidation, as indicated by elevated expression of related genes and an increased respiratory quotient.^148,366,415^ PARP2 is closely involved in cholesterol biosynthesis, and loss of PARP2 mediates cholesterol synthesis by stimulating SREBP1 and SREBP2 in skeletal muscle and liver.^415,417^ PARP2 deletion weakens the expression of hepatic ABCA1, thereby decreasing cholesterol flux.^417^

Multiple investigations have demonstrated that lower PARP activity is related to optimized HDL/LDL ratios and normal triglyceride levels. Pharmacological inhibition of tankyrase could lower serum triglyceride, cholesterol, nonesterified fatty acids, and glycerol in *db/db* mice.^418^ Suppression of PARP1 reduces plasma triglyceride, cholesterol, and LDL levels and enhances HDL levels in HFD-fed mice.^419^

ADP-ribosylation is closely involved in lipid homeostasis. Advances in identifying novel therapeutic targets will provide novel perspectives into targeted drug discovery.

##### Carbonylation in hyperlipidemia

Protein carbonylation induces oxidative stress-related damage. Protein carbonyl level can serve as an indicator of oxidative injury in individuals with familial hypercholesterolemia. Cyanate, a uremic toxin, works via protein carbonylation. Cyanate induces hyperlipidemia by suppressing the Nrf2/HO-1 pathway and mediates oxidative stress via the AMPK-mTOR pathway in mice.^155^

Inhibition of carbonylation is potentially beneficial to reducing oxidative stress and improving lipid metabolism. Annona crassiflora crude extract (CEAc) can improve hyperlipidemia with reduced triglycerides, lower cholesterol and higher HDL in mice by decreasing protein carbonylation and lipid peroxidation.^420^ Low molecular weight galactomannan-based standardized fenugreek seed extract (LMWGAL-TF) could also lower plasma lipids in HFD-fed mice by reducing hepatic FASN and leptin and reducing mitochondrial oxidative stress via downregulated protein carbonylation.^421^ Fish oils, abundant with ω-3 polyunsaturated fatty acids (ω-3 PUFAs), improve hyperlipidemia in high-caloric diet-fed rats through reducing oxidative stress and hepatic protein carbonylation.^422^

Protein carbonylation is a deleterious PTM that disrupts lipid homeostasis. Strategies to restrain protein carbonylation hold the promise of regulating lipid metabolism and improving dyslipidemia.

#### PTMs in atherosclerosis

**Fig. 7:**
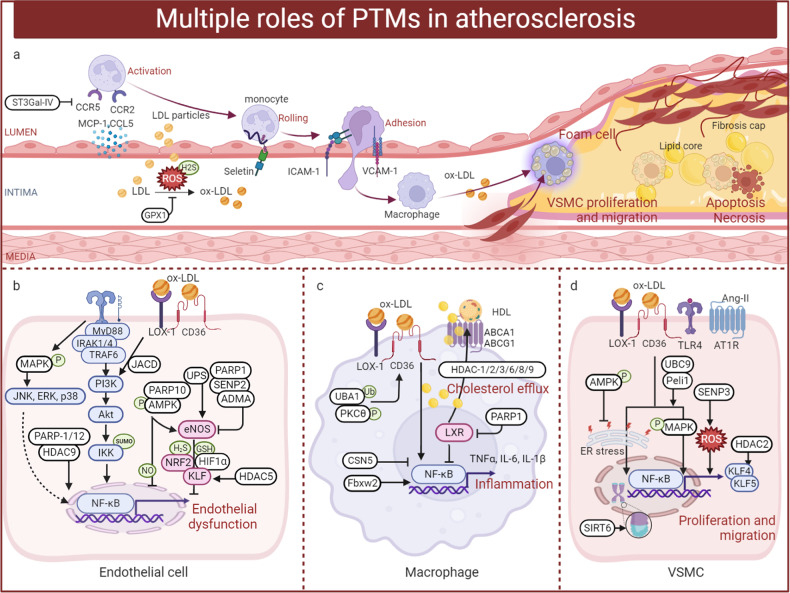
Multiple roles of PTMs in atherosclerosis. Atherosclerosis occurs as a result of multiple risk factors, including abnormal lipid metabolism (hyperlipidemia), endothelial dysfunction, foam cell formation, VSMC proliferation and migration, cell apoptosis and necrosis. **a** The holistic illustration of atherosclerotic progression: monocyte activation, rolling and adhesion; macrophage-derived foam cell formation; VSMC proliferation and migration; atherosclerotic plaque formation. Lipotoxic ox-LDL and inflammation injure endothelial cells, activate the main regulator NF-κB, promote expression of pro-inflammatory genes and induce inflammation. Ox-LDL induces macrophage inflammation and lipid uptake, which will facilitate foam cell formation. ABCA1 and ABCG1-mediated reverse cholesterol transport could alleviate lipid overload and inhibit foam cell generation. Ox-LDL also induces VSMC phenotypic transformation, and promote VSMC proliferation and migration, contributing to atherosclerotic plaque. To understand the comprehensive role of PTMs in atherogenesis, we summarise several pathways of PTMs in regulating the physiology and pathology of endothelial cells, macrophage and VSMC. **b** Endothelial dysfunction. Abundant PTMs regulate endothelial inflammation, such as phosphorylation (oxLDL-MAPK, AMPK-p300, JACD-PI3K/Akt), acetylation (HDAC9-IKK-NFκb, HDAC5-KLF2), methylation (ADMA-iNOS), ubiquitination (UPS-eNOS), SUMOylation (SUMO-IKK, Disturbed flow-SENP2-p53/ERK5-eNOS), glutathionylation (GPX1-oxLDL, GSH-NRF2/HIF1α-NFκB), S-nitrosylation (S-nitrosylated-NO-NFκB), sulfhydration (H_2_S-KEAP1-NRF2, oxLDL-H_2_S-CSE-NF-κB), ADP ribosylation (PARP1-NF-κB/NFAT/AP1, PARP1-eNOS, PARP10-NFκB, PARP12-TRIF-NFκB). **c** Macrophage inflammation and cholesterol efflux. Phosphorylation (PKCθ-ATF2-CD36), acetylation (HDAC1/2/3/6/8/9-ABCA1/ABCG1), ubiquitination (UBA1-oxLDL-NADPH, FBXW2-KSRP), Neddylation (CSN5-NFκB), glycosylation (ST3Gal-IV-CCR5) and ADP ribosylation (PARP1-LXR-ABCA1) regulating macrophage roles in atherosclerotic progression. **d** VSMC proliferation and migration. Phosphorylation (oxLDL-MAPK, AMPK-LDLR-ER stress), acetylation (HDAC2-KLF4/5, SIRT6- telomeres), ubiquitination (Peli1), and SUMOylation (SENP3-ROS, AMPKα2-UBC9/SUMO2/3-GPR120, SUMO-LRH1-PROX1-RCT, UBC9/PIASγ-PPARα, SENP-PPARα/PPARδ, AngII-ATF3-eNOS) mediate the VSMC function in atherogenesis. The blue and red boxes indicate the major pathways in atherosclerosis, and the black boxes indicate the roles of PTM-related molecules and enzymes in atherosclerotic regulation. The figure is generated with BioRender (https://biorender.com). Column1,Column2; ABCA1 ATP binding cassette transporter, ABCG1 ATP binding cassette transporter, ADMA asymmetric dimethylarginine, AMPK AMP-activated protein kinase, AngII angiotensin II, AP-1 activator protein-1, ATF activating transcription factor, CCL5 C-C motif chemokine ligand 5, CCR C-C motif chemokine receptor, CSE cystathionine gamma-lyase, CSN5 COP9 signalosome 5, ER endoplasmic reticulum, eNOS endothelial nitric oxide synthase, ERK5 extracellular signal-regulating kinase 5, GPX1 glutathione peroxidase 1, GSH glutathione, H_2_S hydrogen sulfide, HDAC histone deacetylase, HDL high-density lipoprotein, HIF-1α hypoxia-inducible factor 1α, HRD1 promote degradation protein 1, ICAM-1 intercellular adhesion molecule-1, KEAP1 kelch like ech associated protein 1, KLF kruppel-like factor, LDL low-density lipoprotein, LRH-1 liver receptor homolog-1, LXR liver X receptor, MAPK mitogen-activated protein kinase, MCP-1 monocyte chemoattractant protein 1, NF-κB nuclear factor kappa B, NRF2 nuclear erythroid 2-related factor 2, oxLDL oxidative LDL, PARP poly (ADP-ribose) polymerase, Peli1 Pellino1, PIAS1 protein inhibitor of activated STAT 1, PKC protein kinase C, PROX ROS, reactive oxygen species, SENP1 sentrin/SUMO-specific protease 1, SIRT sirtuin, TRIF toll-interleukin-1 receptor containing adapter-inducing interferon-β, UBA1 ubiquitin-like modifier activating enzyme 1, VCAM-1 vascular cell adhesion molecule 1

Atherosclerosis occurs as a result of multiple factors, including matrix changes, lipid metabolism (hyperlipidemia), vascular changes, chronic unresolved inflammation, endothelial dysfunction, foam cell formation, hemodynamic stress, and blood coagulation. As shown in Fig. 7, dyslipidemia induces endothelial dysfunction, which promotes macrophage and vascular smooth muscle cell (VSMC)-derived foam cell formation, finally leading to the formation of atherosclerotic plaques. PTMs affect protein structures and biological properties, which are closely involved in these pathological processes. Aiming to understand and exploit the molecular mechanisms of atherosclerosis via proteomic investigations, a thorough comprehension of the role of PTMs is essential to gain insights into the potential regulation of the relevant cellular pathophysiology. Here, we will examine various PTMs in the context of atherosclerosis.

##### Phosphorylation in atherosclerosis

Protein phosphorylation is the most widely investigated PTM, and it is closely related to atherosclerosis. Abundant protein kinases have been reported to regulate the progress of atherosclerosis.

MAPKs, a Ser/Thr kinase family, are vital signal-transducing enzymes that are involved in cellular regulation. MAPK family signaling cascades mainly include p38α MAPK, ERK, and JNK.^423^ MAPKs are of relevance to atherosclerosis via matrix production, endothelial cell activation, macrophage inflammation and foam cell formation, VSMC proliferation and migration. MAPK activation mediates monocyte adhesion to activated endothelium mediated by oxidized LDL (ox-LDL).^424^ Apolipoprotein E (*ApoE*^-/-^) mice with p38α MAPK deficiency in macrophages show enhanced macrophage apoptosis in atherosclerotic plaques.^425^ JNK2 decreases the phosphorylation of scavenger receptor-A (Sr-A) cytoplasmic tail serine to fewer foam cells, which finally contributes to smaller plaques in *ApoE*^-/-^*Jnk*^-/-^ mice.^425^ Oxidized LDL induces VSMC proliferation through MAPK activation.^426^ Knockout of MAPK phosphatase-1 (MKP-1) in *ApoE*^-/-^ mice reduces atherosclerosis via reducing the contents of TNFα and interleukin-1α.^427^ In addition, other MAPK members or pathway-related molecules, such as activator protein-1 (AP-1)^428^ and MK2,^429^ are implicated in atherosclerosis via phosphorylation.

AMPK is a conserved Ser/Thr kinase responsible for energy homeostasis and the regulation of cell metabolism. AMPK kinases (including LBK1, CAMKKβ, and TAK1) activate AMPK via phosphorylation at Thr172, coping with depleted cellular ATP levels. AMPK then phosphorylates target proteins to activate catabolic pathways or suppress anabolic pathways to conserve and produce ATP. AMPK has been reported to possess vasoprotective and antiatherosclerotic effects by inhibiting oxidative stress, inflammation, and VSMC proliferation. Specifically, the double knockout of LDLR and AMPKα2 in mice promotes ER stress and accelerates the development of atherosclerosis.^430^ AMPK inhibits endothelial inflammation by phosphorylating the transcriptional coactivator p300 in human umbilical vein endothelial cells (HUVECs).^431^ Sterol SREBP-1c is phosphorylated by AMPK at Ser372, which reduces lipogenic transcription in hepatic cells.^375^

Furthermore, other related kinases and their substrates, including Rho-associated coiled-coil-containing kinases (ROCK),^432^ Akt kinase^17^ and protein kinase C (PKC) are also involved in the development of atherosclerosis.^433^ Ablation of ROCK1 in macrophage results in enhanced cholesterol efflux and thus reduced atherosclerosis.^432^ Junctional cadherin 5 associated (JCAD) aggravates arterial thrombosis and atherosclerosis via PI3K/Akt pathway.^17^ PKCθ enhances CD36 expression through triggering transcription factor 2 (ATF2) and promotes macrophage-derived foam cell formation, leading to atherosclerosis in *ApoE*^-/-^ mice.^433^

The role of protein phosphorylation in the progression of atherosclerosis progression has been well recognized and it is a potential target for atherosclerosis prevention. Further research of novel therapeutic targets in regulating protein phosphorylation is critical for designing novel anti-atherosclerosis approaches.

##### Acetylation in atherosclerosis

Lysine acetylation can affect the process of atherosclerosis mainly via regulating acetylation and deacetylation of histones and nuclear proteins. Plasma interleukin-35 (IL-35) is increased in *ApoE*^-/-^ mice and patients with hypercholesterolemia, and IL-35 suppresses mitochondrial ROS-induced H3K14 acetylation, which inhibits endothelial activation and alleviates the development of atherosclerosis.^434^

Increasing evidence has indicated the essential roles of HDACs in endothelial cell homeostasis and the development of atherosclerosis. The HDAC I, II and IV types are the typical HDACs; class III consists of sirtuin proteins. Among them, HDAC9 is well known because of its essential role in regulating human and mouse atherosclerosis. A genome-wide association meta-analysis (GWAS) reveals that HDAC9 is implicated in atherosclerotic aortic calcification at a genome-wide level and affects VSMC contractility phenotype through regulating calcification.^435^ HDAC9 deficiency in *Ldlr*^-/-^ mice reduces aortic lesion size and protects against atherosclerosis through suppressing cholesterol efflux by inhibiting ABCA1/ABCG1 and PPARγ.^436^ HDAC9 can deacetylate IKK-α/β to activate NF-κB, leading to inflammation in macrophage^437^ and promoting endothelial-mesenchymal transition,^28^ both of which contribute to regulating atherosclerotic vulnerability.

Most typical HDACs are involved in endothelial cell biology, VSMC phenotype determination and macrophage-derived foam cell formation. Metformin-mediated anti-inflammatory role in the endothelium is caused by phosphorylation of HDAC5 and the subsequent activation of KLF2.^438^ Phosphorylated HDAC2 regulates the acetylation of KLF4 and KLF5 in VSMCs, mediating retinoic acid receptor agonist-induced VSMC proliferation.^439^ The VSMC proliferation stimulator Ang-II can activate HDAC5 via G protein-coupled receptor (GPCR)-kinase2 interacting protein 1 (GIT1).^440^ HDAC inhibitors such as TSA and ITF2357 (pan-HDACi) or genetic silencing of HDAC1/2/3/6/8 genes enhances histone acetylation and ABCA1/ABCG1 levels, thus restraining cholesterol accumulation and development of foam cells in macrophages.^440^

Furthermore, sirtuins also play a vital role in the progression of atherosclerosis. SRT3025, a SIRT1 activator, inhibits atherosclerosis in *ApoE*^-/-^ mice through reducing hepatic PCSK9 expression and increasing LDLR expression.^441^ Human and mouse plaque VSMCs exhibit reduced SIRT6 expression. SIRT6 bounds to telomeres, affects H3K9 deacetylation and 53BP1 (p53 binding protein 1) binding, which preserves telomere integrity and extends VSMC lifespan and thus inhibits atherosclerosis.^442^

The balance of protein acetylation and deacetylation is tuned to govern cell biology and thus regulate atherosclerosis. Dysregulation of protein acetylation represents a cause of cardiovascular comorbidities. Advances in deciphering the role and mechanism of protein acetylation in atherosclerosis will potentially provide novel therapeutic targets in treating atherosclerosis.

##### Methylation in atherosclerosis

Protein methylation has been well recognized for regulating the function of nuclear and nucleic acid-binding proteins and to have an essential role in CVDs.

Histone methylation is of great importance in atherosclerosis. The histone methylation profile in human atherosclerotic lesions reveales that global H3K27me2 and H3K9me2 are reduced in atherosclerotic plaques, whereas H3K4me2 shows comparable levels in both atherosclerotic and normal carotid arteries.^443^

Furthermore, histone methyltransferases and a few intermediate products during the enzymatic process are implicated in atherosclerotic progression. Myocardin-related transcription factor A (MRTF-A) recruits the H3K27 methyltransferase ASH2, transactivates inducible nitric oxide synthase (iNOS), inducing endothelial inflammation.^36^ The Set7/9 lysine methyltransferase directly methylates the FXR at lysine 206 to regulate a series of target genes of bile acid homeostasis. These genes encode estrogen receptor, androgen receptor, p53, TAF10, p300/CBP-associated factor and RelA subunit of NF-κB, which are associated with atherosclerosis.^444^ PRMT and ensuing proteolysis of proteins generate asymmetric dimethylarginine (ADMA). ADMA regulates ROS by inhibiting NOS and reducing NO production. Plasma ADMA level is shown to increase in individuals with coronary artery disease,^445^ and ADMA is suggested to act as a risk marker.

Much more work is necessary to better understand the role of protein methylation in atherosclerosis. Advances in this area could afford new perspectives and guide treatments for atherosclerosis.

##### Ubiquitination in atherosclerosis

The ubiquitin-proteasome cascade pathway is crucial to protein metabolism and endogenous protein degradation in eukaryotic cells. The UPS is implicated in a wide range of physiological processes, including endothelial dysfunction, apoptosis, oxidative stress and foam cell formation, which are relevant to atherosclerosis. E1, E2, and E3 ligases are implicated in atherosclerotic progression.

UBA1 is a major E1-activating enzyme in the UPS cascade. UBA1 inhibitor PYR-41 can inhibit ox-LDL-induced proinflammatory cytokine expression, NADPH oxidases and lipid deposition in macrophage, thereby suppressing atherosclerosis in *ApoE*^-/-^ mice with blunted proinflammatory responses in macrophage.^446^

Aggregated low-density lipoprotein (agLDL)-induced ubiquitin-conjugating E2 enzyme E2-25K facilitates lipid-bearing macrophages apoptosis via ubiquitination and degradation of p53, leading to foam cell formation.^447^

E3 ubiquitin ligases are recognised to play diverse and multifaceted roles in atherosclerosis-related inflammatory and metabolic processes. TRIM21 deficiency facilitates Th17 differentiation and promotes IL-17 expression, thereby enhancing collagen content in plaques and improving atherosclerotic plaque stability in *Ldlr*^-/-^ mice.^47^ The E3 ligase IDOL triggers the degradation of LDLR. Transgenic expression of hepatic IDOL results in reduced LDLR and aggravated atherosclerosis in western diet-fed C57Bl/6J mice.^448^ Deleting the E3 ligase Pellino1 (Peli1) facilitates VSMC foam cell formation, induces proinflammatory cytokines and promotes atherosclerosis.^449^ E3 ubiquitin ligase FBXW2 can promote macrophage inflammation and atherosclerosis by ubiquitinating KH‐type splicing regulatory protein (KSRP) and reducing proinflammatory cytokines. Inhibition of FBXW2 might serve as a potential approach to treating atherosclerosis.^450^

Since the critical role of protein ubiquitination and the UPS system in atherosclerosis has gained widespread recognition, further exploration of new therapeutic approaches regulating ubiquitin ligases could contribute to the treatment of atherosclerosis.

##### SUMOylation in atherosclerosis

Recent studies have indicated that SUMOylation is implicated in dyslipidaemia, endothelial dysfunction, and VSMC proliferation, all processes which trigger the initiation and development of atherosclerosis. Next, we will elaborate on the role of SUMOylation in atherosclerosis from these three aspects.

Dyslipidemia in atherosclerosis is a crucial metabolic risk factor that includes high serum TG levels, increased LDL levels, and decreased HDL levels. SUMOylated LRH-1 interacts with PROX1 to inhibit RCT and thereby promotes atherosclerosis^396^. SUMO E2 enzyme UBC9 and SUMO E3 ligase PIASγ induce dyslipidaemia by SUMOylating PPARα at the K185 site and suppressing PPARα transcriptional activity.^451^ Correspondingly, SENPs promote fatty acid β-oxidation by eliminating SUMOylation in PPARα and PPARδ.^452^

During endothelial cell dysregulation, deficient nitric oxide (NO), enhanced adhesion molecules, endothelial apoptosis and senescence contribute to atherosclerotic pathology. SENP1 deficiency directly increases SUMOylation of GATA binding factor 2 (GATA2) and nuclear factor of kappa light polypeptide gene enhancer in B-cell inhibitor alpha (IκBα), leading to decreased GATA2 stability and NF-κB activity, thus reducing adhesion molecules expression.^453^ Disturbed flow causes phosphorylation of SENP2 via p90RSK kinase, which promotes SENP2 activity and downregulates SUMOylation of p53 and ERK5 under disturbed flow, leading to endothelial dysfunction evidenced by reduced eNOS and atherosclerotic plaque formation in *Ldlr*^-/-^ mice.^454^ The SUMOylation of activation of transcription factor 3 (ATF3) could aggravate endothelial dysfunction induced by angiotensin II (Ang-II) by decreasing NO generation.^455^

It has been proposed that SUMOylation is essential to promote VSMC proliferation. Ox-LDL and Ang-II can increase SENP3 expression via a ROS-dependent way in VSMC. SENP3 overexpression facilitates VSMC proliferation, migration and vascular remodeling through suppressing de-SUMOylation of β-catenin.^456^ AMP-activated protein kinase α2 (AMPKα2) activation inhibits GPR120 SUMOylation via suppressing SUMO2/3 and Ubc9 in VSMC, mediating the anti-inflammatory and atheroprotective impacts of fish oil.^57^

From this perspective, protein SUMOylation plays a diverse and critical role in atherosclerosis. Further elucidation of protein SUMOylation in atherosclerosis could yield novel therapeutic targets of atheroprotection.

##### Neddylation in atherosclerosis

Studies have shown that protein neddylation is associated with dysfunction of the endothelium and macrophage activation.

Ox-LDL induces global neddylation, and overexpression of NEDD8 can reduce HDAC2 levels in human aortic endothelial cells (HAECs). Suppressing neddylation with the neddylation activating enzyme (NAE) inhibitor MLN4924 relieves ox-LDL-mediated endothelial dysfunction.^457^ MLN4924 can decrease inflammation in macrophages and endothelial cells. MLN4924 also increases HDAC6 activity, reducing endothelial dysfunction and atherosclerosis in vivo.^458^

The de-neddylation enzyme COP9 signalosome 5 (CSN5) was proposed to inhibit atherosclerosis by regulating macrophage activation through inhibiting NF-κB.^63^ Therefore, de-neddylation approaches may serve as a candidate strategy to decrease atherogenesis.

Much more exploration is required for a better understanding of the relationship between protein neddylation and atherosclerosis. Discoveries in this field might potentially identify novel therapeutic targets in treating atherosclerosis.

##### Glycosylation in atherosclerosis

Atherosclerosis is a complex and chronic inflammatory disease in which, in one early aspect, leukocytes are recruited to the arterial intima from the blood through a series of steps, including tethering, rolling, adhesion and transmigration, processes driven by adhesion molecules and chemokines. Glycosylation and various glycosyltransferases are contributors to atherosclerosis, as chemokine receptors and adhesion molecules are usually glycosylated proteins.

The lack of several glycosyltransferases inhibits atherosclerosis by decreasing leukocyte recruitment. α2,3-Sialyltransferase IV (ST3Gal-IV) deficiency in mice protects against atherosclerosis, as the glycosylation of C-C motif chemokine receptor 5 (CCR5) is impaired, leading to decreased leukocyte rolling.^74^ Loss of α(1,3) fucosyltransferases-IV (FucT-IV) and FucT-VII reduces the glycosylation of selectin ligands and leukocyte recruitment, decreasing atherosclerotic lesions.^459,460^ Core 2 β1,6 galactosyltransferase I deletion prevents atherosclerosis by reducing glycoproteins in selection ligands such as CD43, CD44, and CD162 and inhibiting their activity.^461^

Elevated levels of the protein glucose adducts and advanced glycosylation end products (AGEs) are found in individuals with diabetes. Increased AGEs promote oxidative stress and contribute to cardiovascular diseases. AGEs are produced mainly by protein and lipid nonenzymatic glycosylation. AGEs and receptor for AGE (RAGE) initiate signaling pathways, such as promoting adhesion molecule expression through the NF-κB pathway and activating NADPH and NOS.^462^

Further elucidation is necessary to better clarify the role of glycosylation in atherosclerosis, which might hold the potential to explore novel therapeutic drugs in CVD and its clinical sequelae.

##### Palmitoylation in atherosclerosis

Palmitoylation affects membrane fusion and cellular trafficking of proteins and is reversibly controled by palmitoyltransferases and acyl protein thioesterase (APT).

Palmitoylation is needed to target and localize endothelial nitric oxide synthase (eNOS) to the caveolae for optimal NO release.^463^ In endothelial cells with FASN deficiency, reduced eNOS palmitoylation causes impaired angiogenesis and endothelial dysfunction.^464^ ATP-1 insufficiency leads to prolonged palmitoylation of Ras-related protein (R-Ras), causing impaired trafficking of eNOS synthase and thus reduction of NO and endothelial dysfunction.^463^

Palmitoylation dysregulation could also cause abnormal foam cell formation. Oxidized high-density lipoprotein (ox-HDL) promotes CD36 palmitoylation, causes CD36 localization to lipid rafts and activates downstream pathway, which finally increases ox-HDL uptake in macrophages and foam cell development. Bromopalmitate-mediated suppression of CD36 palmitoylation reduces cell surface translocation and lightens oxHDL uptake.^79^

Limited knowledge of the function of protein palmitoylation in atherosclerosis hindered the research to explore novel strategies targeting palmitoylation, thereby more research is warranted to elaborate on the relationship between palmitoylation and atherosclerosis.

##### Myristoylation in atherosclerosis

Since eNOS dysfunction produces superoxide instead of NO, leading to endothelial dysfunction and atherosclerosis, eNOS is a potential therapeutic target in atherosclerosis. eNOS is acylated by palmitoylation and myristoylation and resides in caveolae and Golgi. N-myristoylation is required for eNOS acylation in the Golgi complex and is of great significance in determining eNOS activity.

Myristoylated pseudosubstrate of PKCζ (mPS), a myristoylated peptide inhibiting PKC activity, activates eNOS in endothelial cells. Myristoylation-induced eNOS activation depends on PI3K/Akt signaling and elevated cellular calcium. Other myristoylated peptides can also activate eNOS in a non-PKC-dependent manner.^465^

Moreover, LIM and cysteine-rich domains 1 (LMCD1) were recently reported to govern the proliferation and migration of human and mouse smooth muscle cells (SMCs) through controling NFATC1-mediated IL33 and E2F1-mediated CDC6 expression. LMCD1 myristoylation inhibits this regulation.^89^

Since very little information about myristoylation and atherosclerosis is available, much more work is required to offer a comprehensive understanding of myristoylation in atherosclerosis.

##### Prenylation in atherosclerosis

Protein prenylation is implicated in atherosclerosis through regulating lipid metabolism, smooth muscle cell proliferation and macrophage inflammation.

Prenylcysteine oxidase (PCYOX1) was recently found to be restricted to the metabolism of protein prenylation, which was related to lipoprotein oxidation and the atherogenic ApoB100 lipoproteins.^94^ PCYOX1 insufficiency in *ApoE*^-/-^ mice attenuates the consequences of atherosclerosis by reducing lesion vulnerability, decreasing lipid peroxidation, and lowering serum lipids and inflammation.^94^ The farnesylation of small G-proteins is essential for cell growth and differentiation. Blocking the enzyme protein farnesyltransferase (PFT) suppresses the proliferation of human SMCs by suppressing the farnesylation of Ras protein.^466^ Inhibition of RAC1 farnesylation by geranylgeranyltransferase type I (GGTase-I) causes enhanced proinflammatory signaling in macrophages.^94^

The normal functioning of protein prenylation is of importance, and targeting protein prenylation might provide a novel opening in anti-atherosclerosis therapy.

##### Glutathionylation in atherosclerosis

Glutathione is a plentiful intracellular small-molecule antioxidant that is implicated in numerous cellular redox processes.

The pathological process of CVD is considered to involve changes in GSH concentration and oxidation state. Serum S-glutathionylated protein levels are elevated in patients with atherosclerosis. S-glutathionylated ApoB100 (an atherogenic lipoprotein) is positively related to peripheral vascular damage.^467^ The synthesis of GSH in macrophages was found to be negatively related to the pathogenesis of atherosclerosis. Ribo-cysteine-treated mice show an increase in the level of GSH and a significant reduction in circulating apoB, lipoprotein(a) and oxidized lipid.^468^

Glutathionylation-related key enzymes contribute to the regulation of atherosclerosis. In a study investigating the glutathione peroxidase 1 (GPX1) polymorphism in individuals with T2DM, GPX1 was found to possess a protective role in human endothelial cell dysfunction and atherosclerosis.^469^ GPX1 deficiency in diabetic *ApoE*^-/-^ mice increases LDL oxidation and accelerates atherosclerosis.^470^ Ox-LDL restrains glutathione reductase (GR) activity and facilitates accumulating protein S-glutathionylation, ROS generation, and cell death in macrophages, promoting the development of atherosclerosis.^471^

Glutathionylation serves as an endothelial cell redox switch. SIRT1 glutathionylation restrains enzymatic activity through changing the protein structure and attaching to NAD^+^, causing endothelial cell apoptosis and senescence.^472^ S-glutathionylation inhibits p65 and p50 subnit of NF-κB, leading to angiogenesis and cell survival.^473^ S-glutathionylation also promotes anti-oxidation, anti-inflammation, and angiogenesis targeting HIF-a^109^ and KEAP1 (NRF2 inhibitor).^474^

Protein S-glutathionylation mediates oxidant-induced PTMs and has emerged as a vital redox regulator in macrophage and endothelial cell dysfunction. Advances in deciphering the underlying mechanism of how protein prenylation regulates atherosclerosis could facilitate novel perspectives in preventing atherosclerosis.

##### S-nitrosylation in atherosclerosis

NO acts as an endogenous mediator of cell respiration and regulator of cardiovascular physiology. NO exerts its influence via cyclic guanosine monophosphate (cGMP) or S-nitrosylated modification. Almost all primary cardiovascular functions of NO are implicated in S-nitrosylation.

S-nitrosylation regulates endothelial inflammation. S-nitrosylation inhibits proinflammatory cytokines and adhesion molecules by blocking the NF-κB pathway.^475^ S-nitrosylation regulates platelet activation in the development of atherosclerosis. NO suppresses platelet aggregation via S-nitrosylation.^476^ eNOS knock-out mice exhibit upregulated leukocyte rolling, increased exocytosis, and enhanced arteriolar thrombosis.^477^ Heat shock protein 90 (HSP90) S-nitrosylation inhibits the HSP90-ATPase activity 1 (AHA1) interaction but stimulates the HSP90-cell division cycle 37 (CDC37) association, which modulates endothelial dysfunction and exacerbates atherosclerosis.^478^ Guanine nucleotide-binding protein G(i) subunit alpha-2 (SNO-GNAI2) S-nitrosylation increases in individuals with diabetes and atherosclerosis. S-nitrosylation mediates the attachment of GNAI1 and C-X-C chemokine receptor type 5 (CXC-R5), inducing Hippo/YAP and accelerating atherosclerosis in diabetic mice.^120^

S-nitrosylated modifications play a role in cardiovascular disease and atherosclerotic progression via regulating NO activity. Further investigation of S-nitrosylation in cardiovascular function might offer us novel perspectives and opportunities in cardiovascular disease prevention research.

##### Sulfhydration in atherosclerosis

Hydrogen sulfide (H_2_S) as a dissolved gas is recognized as a member of the family of gasotransmitters. A considerable amount of evidence has suggested that H_2_S can protect against atherosclerosis. The atherosclerotic protective mechanisms of H_2_S include endothelium preservation, anti-inflammation, antioxidative responses, and vasorelaxation.^128^

S-sulfhydration has been recognized as one of the main mechanisms determining the physiological effects of H_2_S. A few studies have demonstrated H_2_S signaling via S-sulfhydration in attenuating atherosclerosis. KEAP1 protein is sulfhydrated at cysteine151, which enhanced KEAP1 thiolation, promoted NRF2 translocation and inhibited superoxide generation in endothelial cells. These increased NRF2-related antioxidant actions ameliorated the subsequent diabetes-accelerated atherosclerosis.^479^ Cystathionine γ-lyase (CSE) and cystathionine β-synthase (CBS) are two enzymes associated with the biosynthesis of H_2_S. Ox-LDL downregulates CSE/ H_2_S signaling and activates inflammation via the NF-κB signaling pathway in macrophages.^480^ Treatment with an H_2_S donor was found to cause CSE thiolation (C252, C255, C307, C310), boost its linkage to L-homocysteine, and alleviate hyperhomocysteinemia-related atherosclerosis in mice.^127^

The ordinary functioning of H_2_S regulated by the S-sulfhydration modification is pivotal in cellular biology. More research targeting the comprehensive role of S-sulfhydration in diverse physiologic and pathologic scenarios could potentially provide us with a new guide in anti-atherosclerosis therapies.

##### Citrullination in atherosclerosis

PADs catalyze the transition of arginine to citrulline, accordingly modifying protein charge and structure. Protein citrullination is associated with rheumatoid arthritis but there is only sparse evidence of its role in atherosclerosis.

Clinical evidence has revealed the relationship between citrullinated proteins and coronary heart disease. Several citrullinated proteins and PAD4 were identified in human coronary artery plaques. Anti-citrullinated protein antibodies (ACPAs) are related to the aortic plaque burden.^481^ High concentrations of serum ACPAs targeting Cit-histone H2B are also associated with elevated coronary artery calcium scores.^139^ These data suggest the possible role of citrullinated proteins as biomarkers for atherosclerosis.

Evidence of citrullination in regulating atherosclerosis is sparse, further exploration is needed to afford a comprehensive understanding of the effect of S-nitrosylation in atherosclerosis.

##### ADP ribosylation in atherosclerosis

PARP1 is the first identified and the classical PARP member that is activated by abnormal DNA or RNA breaks and mediates approximately 90% of total cellular PARP responses.^482^ PARP1 participates in atherosclerosis by regulating cholesterol efflux, inflammation, endothelial cell dysfunction, foam cell death, and cellular energy crisis. PARP1 can PARylate LXR and inhibit LXR-induced ABCA1 expression, thus reducing cholesterol efflux in macrophages.^413^ Proatherogenic factors (such as proinflammatory cytokines) cause PPAR1 hyperactivity, inducing overactivation of NF-κB, activated T cells (NF-AT), and the AP-1 pathway.^483,484^ Blocking PPAR1 improves endothelial dysfunction by boosting eNOS activity,^485^ reducing adhesion molecules,^486^ and advancing endothelium-dependent relaxation.^149^ PPAR1 inhibition decreases macrophage recruitment,^487^ and acetyl-coenzyme A acetyltransferase-1 (ACAT-1) mediates foam cell death and atherosclerosis in *ApoE*^-/-^ mice.^488^ Hyperactivation of PPAR1 also leads to exhaustion of NAD^+^ and ATP, resulting in a cellular energy crisis.^489^

In addition to PPAR1, increasingly more other PPARs have been identified to participate in tissue inflammation. PPAR9 and PPAR14 together modulate the proinflammatory activation of macrophages.^490^ PPAR14-mediated SIRT1 ADP ribosylation appears to decrease the phosphorylation of SIRT1.^490^ PPAR10 ADP-ribosylates NF-κB essential modulator (NEMO) and suppresses its polyubiquitination, inhibiting NF-κB translocation.^491^ PARP12 binds to toll-interleukin-1 receptor containing adapter-inducing interferon-β (TRIF) and increases NF-κB-dependent gene expression.^492^

The diverse role of ADP ribosylation in endothelial dysfunction and atherosclerosis has gained widespread explanations. Further investigation of new therapeutic targets in regulating ADP ribosylation is crucial for developing novel anti-atherosclerosis therapies.

##### Carbonylation in atherosclerosis

Protein carbonylation is usually a deleterious PTM, and inhibition of carbonylation will potentially alleviate oxidative stress and modulate atherosclerosis.

In vitro, ox-LDL induces carbonylation of p65 of NF-κB, potentially worsening LPS-induced macrophage inflammation and related atherosclerosis.^493^

In vivo, D-carnosine octyl ester alleviates atherosclerosis in *ApoE*^-/-^ mice by decreasing general serum carbonylated protein levels.^494^ Oolong tea derived-oolonghomobisflavan A (OFA) prevents atherosclerosis by reducing oxidative stress and carbonylation of ApoB100.^156^

Protein carbonylation is usually a deleterious PTM that disrupts normal cellular functions. Strategies to restrain protein carbonylation could benefit regulating cell homeostasis and prevent atherosclerosis.

### Pharmaceutical intervention of PTMs in preclinical studies

The preclinical research of metabolic diseases which target protein modifications is an interesting field of study. We next discuss some representative compounds in preclinical studies and elucidate how these compounds impact each PTM in metabolic diseases. Detailed information can be seen in supplementary Table 1 which summarizes the pharmaceutical interventions of protein modifications in preclinical studies of metabolic diseases.

#### AMPK activators

Metformin is an AMPK activator that improves glucose control and insulin sensitivity, thus decreasing intestinal glucose absorption. Metformin promotes phosphorylation of AMPK and glucose production in primary rat hepatocytes.^495^

Ampkinone plays an indirect action in the phosphorylation of AMPK. DIO mice treated with ampkinone show reduced body weight, decreased fat mass weight and improved metabolic characteristics.^496^

In addition, the AMPK activator AICAR also inhibits the phosphorylation of IRS-1 by suppressing ERK phosphorylation. In *db/db* mice, intraperitoneal administration (0.25 g/kg) of AICAR can improve blood glucose levels by inhibiting the phosphorylation of ERK in adipose tissue.^497^

#### MAP kinase inhibitors

SD-169 is a selective ATP-competitive inhibitor of MAP kinases. SD-169 treatment provides a hypoglycemic effect and preserves beta cell mass in NOD mice.^498^

U0126 inhibits the MAPK/ERK pathway to ameliorate diabetic cardiomyopathy in STZ-induced diabetic mice. U0126 also reduces de novo fatty acid synthesis by inhibiting liver FASN expression. U0126 inhibits atherosclerosis in LDLR-deficient mice without adipogenesis side effects.^499^

#### PTP1B inhibitor

KY-226 is an inhibitor of PTP1B. Oral administration of KY-226 can increase the insulin-induced phosphorylation of insulin receptors, and reduce plasma glucose and triglyceride levels in *db/db* mice. In high-fat-diet-induced obese mice, KY-226 reduces body weight gain and increases phosphorylated STAT3 in the hypothalamus.^500^

JTT-551 has been used in the research of T2DM. In *db/db* mice, chronic administration of JTT-551 shows an anti-hyperglycemic effect. A single dose of JTT-551 in *ob/ob* mice enhanced the IR phosphorylation of the liver.^501^

MSI-1436 selectively inhibits PTP1B and enhances insulin-stimulated tyrosine phosphorylation of insulin receptors, causing fat-specific weight loss in DIO mice.^502^ DPM-1001 is an analogue of MSI-1436, which can enhance β-subunit phosphorylation and reduce diet-induced obesity.^503^

#### JAK1/2 inhibitor

Ruxolitinib is a selective JAK1/2 inhibitor. Inhibiting phosphorylation of the JAK2/STAT3/SOCS3 pathway significantly decreases the area of atherosclerotic plaques and balloon injury of the aorta in rabbits fed with HFD.^504^

Fedratinib (TG101348) is an ATP-competitive inhibitor of JAK2. TG101348 selectively decreases cellular phosphorylated STAT5 and substantially reduces aortic atherosclerosis in *ApoE*^-/-^ mice.^505^

#### EGFR inhibitor

Erlotinib is a direct-acting inhibitor of EGFR tyrosine kinase and reduces EGFR autophosphorylation. Erlotinib can block EGFR-mediated STAT3 phosphorylation, significantly reducing myocardial structural and functional deficits in diabetic mice.^506^

Compound Y396 could inhibit the phosphorylation of EGFR. Y396 inhibits endothelial cell dysfunction induced by oxidative stress in diabetes.^507^

#### Histone deacetylase inhibition

Valproate improves kidney function in DN rat models by modulating acetylation of histone H4 in ERS-associated protein promoter.^508^

MGCD0103, an HDAC inhibitor, protects the pancreas from STZ-induced hyperglycemia, macrophage infiltration and oxidative stress.^509^

Vorinostat (SAHA) treatment ameliorates HFD-induced reduction of histone H3 acetylation and reverses memory impairment in insulin-resistant cognitively deficient mice by reducing BDNF levels.^510^

Tubastatin A is a specific HDAC6 inhibitor. Tubastatin A treatment reduces food intake, fat mass, hepatic steatosis and improves systemic glucose homeostasis by restoring leptin sensitivity.^511^

#### H3K27me3 demethylase inhibitor

GSK-J4 is the inhibitor of H3K27me3 demethylase towards KDM6B and KDM6A. It can reduce obesity-related properties in diet-induced obesity mice via the sensitised leptin signaling.^512^

#### Histone methyltransferase inhibitor

BIX01294 is an inhibitor of G9a histone methyltransferase. G9a histone methyltransferase mediates the phosphorylation of FOXO1 both in vivo and in vitro, regulating obesity and associated diseases.^287^

GSK126 is a specific EZH2 methyltransferase inhibitor. GSK126 alleviates obese phenotypes by promoting diet-induced differentiation of thermogenic beige adipocytes in obese mice and decreasing EZH2 enzymatic activity.^513^

#### USP30 inhibitor

MF-094 is a selective inhibitor of USP30. MF-094 can accelerate diabetic wound healing by deubiquitinating NLRP3 to inhibit the NLRP3 inflammasome.^514^

#### Ubiquitin-activating enzyme E1

PYR-41 is the first cell-permeable inhibitor of ubiquitin-activating enzyme E1, but invalid to E2. PYR-41 inhibits the ubiquitin-activating enzyme UBA1 to alleviate atherosclerosis and suppress the inflammatory response of macrophage in *ApoE*^-/-^ mice.^446^

#### DUB inhibitor

GSK2643943A is a DUB inhibitor. GSK2643943A decreases the USP20 hydrolyzed K48- and K63-linkages and reduces HMGCR ubiquitination to inhibit diet-induced cholesterol biosynthesis.^515^

#### NEDD8-activating enzyme inhibitor

MLN4924 is a kind of small molecule NEDD8-activating enzyme (NAE) inhibitor. It has been demonstrated that MLN4924 effectively prevents diet-induced obesity and the disorder of glucose metabolism in mice.^516^ Similarly, MLN4924 causes pharmacological inhibition of cullin neddylation, which prolongs insulin action in hepatocytes and decreases hepatic glucose production in mice.

#### PARP inhibitors

PJ34 is a potent specific inhibitor of PARP1/2, that can reduce PARP activity, GAPDH ribosylation, and GAPDH translocation in the mouse model of T2DM.^517^

## Clinical trials of metabolic diseases by targeting protein modifications

Growing evidence corroborates the essential role of PTMs in metabolic diseases. Families of drugs targeting protein modification enzymes include kinase agonists and inhibitors, HDAC inhibitors, histone methyltransferase inhibitors, and others. Here, we focus on clinical trials of metabolic diseases by agents that target protein modification. Table 1 shows the current clinical trials of metabolic diseases by targeting protein modification.

**Table 1:** Clinical trials of metabolic diseases by targeting protein modifications

No.	Drug	Modification	Mechanism	Phase	NCT number
1	Atorvastatin	Prenylation	An HMG-CoA reductase inhibitor; Inhibits the posttranslational prenylation of small guanosine triphosphate binding proteins.	II	NCT02633956
2	Curcumin	Phosphorylation Ubiquitination O-GlcNAcylation	An ACC inhibitor; Phosphorylates and inactivates ACC; Inhibits protein ubiquitination; Inhibits O-GlcNAcylation pathway.	IV	NCT01052025
3	MK-4074	Phosphorylation Ubiquitination O-GlcNAcylation	An ACC inhibitor; Phosphorylates and inactivates ACC; Inhibits protein ubiquitination; Inhibits O-GlcNAcylation pathway.	I	NCT01431521
4	PF-05221304	Phosphorylation Ubiquitination O-GlcNAcylation	An ACC inhibitor; Phosphorylates and inactivates ACC; Inhibits protein ubiquitination; Inhibits O-GlcNAcylation pathway.	I II	NCT03248882 NCT03776175
5	Firsocostat	Phosphorylation Ubiquitination O-GlcNAcylation	An ACC inhibitor; Phosphorylates and inactivates ACC; Inhibits protein ubiquitination; Inhibits O-GlcNAcylation pathway.	II	NCT02856555
6	Resveratrol	Acetylation	Decreases PGC-1α acetylation.	I	NCT02704494
7	Selonsertib (GS-4997)	Phosphorylation	An ASK1 inhibitor; Phosphorylates p38/JNK.	II IIb N/A I II II III III	NCT02177786 NCT04026165 NCT02781584 NCT02509624 NCT02466516 NCT03449446 NCT03053063 NCT03053050
8	BGP-15	Phosphorylation	A PARP inhibitor; Phosphorylates Akt and GSK-3, prevents p38/JNK activation.	II	NCT01069965
9	Tropifexor	Acetylation	A FXR agonist; Acetylates FXR; Blocks connection of FXR and the SUMO ligase PIASγ.	II II	NCT02855164 NCT02854605
10	Cilofexor	Acetylation	A FXR agonist, acetylated FXR Blocks connection of FXR and the SUMO ligase PIASγ.	II II II	NCT02854605 NCT03449446 NCT03987074
11	Emricasan	Phosphorylation Nitrosylation Ubiquitination SUMOylation Acetylation	A pan-Caspase inhibitor; Inhibits SUMOylation of the ligand-binding domain.	II II	NCT02686762 NCT03205345
12	Dorzagliatin	SUMOylation Nitrosylation	A GK agonist; Promotes SUMOylation of pancreatic glucokinase.	II	NCT03141073
13	MK-0941	SUMOylation Nitrosylation	A GK agonist; Promotes SUMOylation of pancreatic glucokinase.	III	/
14	AZD1656	SUMOylation Nitrosylation	A GK agonist; Promotes SUMOylation of pancreatic glucokinase.	II II	NCT01152385 NCT01020123
15	Trodusquemine	Phosphorylation Nitrosylation Sumoylation	A PTP1B inhibitor; Inhibits phosphorylation, nitrosylation and SUMOylation at active site Cys215.	I I I I	NCT00509132 NCT00806338 NCT00606112 NCT02524951
16	Sodium phenylbutyrate	Acetylation	A HDAC inhibitor; Inhibits histone deacetylase.	I	NTR7426

*HMG-CoA* 3-hydroxymethylglutaryl coenzyme A, *ACC* acetyl-CoA carboxylase, *FASN* fatty acid synthase, *AMPK* AMP-activated protein kinase, *PGC* peroxisome proliferator-activated receptor-gamma coactivator, *PARP* poly (ADP-ribose) polymerase, *ASK1* apoptosis signal-regulating kinase 1, *JNK* Jun N-terminal kinase, *TGF* transforming growth factor, *FXR* farnesoid X receptor, *GK* glucokinase, *PTP1B* protein tyrosine phosphatase 1B

### HMG-CoA reductase inhibitors (Statins)

The 3-hydroxymethylglutaryl coenzyme A reductase inhibitors (statins) is an effective prevention strategy for vascular diseases such as cardiovascular diseases and stroke for more than 20 years. Statins are used as hypolipidemic drugs which act as a primary response by inhibiting cholesterol synthesis. Statins reduce cholesterol biosynthesis, mainly in the liver, and consequently modulate endogenous lipid metabolism. The anti-atherosclerotic effects of statins are correlated with reduced LDL cholesterol levels. In addition, statins exert a cardiovascular protective impact by non-lipid dependent actions referred to as “pleiotropic” effects. Because statins lower the intermediates of the mevalonate pathway beyond cholesterol, they inhibit the posttranslational prenylation of small guanosine triphosphate-binding proteins and their downstream effectors.^518^ To date, statins also confer protective effects against NAFLD in addition to cardiovascular protection. In a clinical research using atorvastatin, the severity of NAFLD activity ratings were dramatically reduced in individuals with dyslipidemia (NCT02633956).^519^ Statins were also discovered to lower the risk of NAFLD in a 6-year follow-up cohort of more than 11 million individuals.^520^

### ACC inhibitors

ACC is a rate-limiting molecule in DNL that converts acetyl-CoA to malonyl-CoA. ACC1 and ACC2 are two isoforms of ACC in mammals. AMPK phosphorylates ACC1 Ser79Ala and ACC2 Ser212Ala, regulating the fatty acid synthesis and oxidation.^374,521,522^ ACC inhibitors fall into two main classes: natural and non-natural (synthetic) compounds. These compounds inhibit ACC activity in three ways: they phosphorylate and inactivate ACC by activating AMPK, inhibiting carboxyltransferase (CT) activity, and acting as biotin carboxylase (BC) inhibitors. Curcumin, a natural compound found in turmeric, influences metabolism through AMPK/ACC pathway as mentioned previously. Curcumin also inhibits the O-GlcNAcylation pathway to restore aggravated liver metabolic damage and improve lipid accumulation.^523^ In a randomized, double-blind, placebo-controlled trial with 240 participants, researchers looked at the effectiveness of curcumin in preventing the onset of T2DM in a group of prediabetics. The data demonstrated that it can effectively prevent prediabetes from developing T2DM (NCT01052025).^524^ According to research by Lu et al., giving mice with gestational diabetes a high dosage of curcumin (100 mg/kg) might reduce their glucose and insulin intolerance. In a group of 118 T2DM patients, Panahi et al. conducted an RCT with a focus on the effects of curcuminoids on ghrelin, adiponectin, leptin, and TNF. When the treatment (1000 mg of curcumin plus 10 mg of piperine per day) was administered to patients with T2DM for 12 weeks, patients showed an inrecese in adiponectin and a decrease in the leptin: adiponectin ratio, leptin levels and TNF levels, independently of weight changes.^525^ A meta-analysis of nine trials with a combined total of 262 animals revealed that curcumin had a significant impact on blood vessel density and the pace of wound healing, suggesting that it could be a likely candidate for the treatment of diabetic foot ulcers.^526^

MK-4074 is a type of pan-ACC inhibitor and a liver-selective medication that dose-dependently reduces liver lipids in humans. A phase I, randomized, double-blind, and placebo-controlled clinical study that included 31 adults with fatty liver disease investigated the effect of MK-4074; however, this research was unfinished (NCT01431521).

The liver receives a preferential distribution of the ACC inhibitor PF-05221304, which is selective, orally accessible, and reversible. It has been tested in phase I and II clinical trials, which showed that with PF-05221304 monotherapy, liver fat reductions reached 50–65% (NCT03248882). When the drugs PF-05221304 and PF-06865571 were administered together, liver fat was reduced in contrast to the placebo; after 6 weeks with either drug alone, the placebo-adjusted LSM decrease in liver fat was -44.5% or -35.4%, respectively (NCT03776175). The findings of two trials show that raising the dose of PF-05221304 did not result in an overall rise in the frequency of adverse events, and that co-administration of PF-05221304 and PF-06865571 may be able to circumvent some of the drawbacks of ACC inhibition alone.^527^

Firsocostat (GS-0976), also known as ND-630, has been assessed in clinical trials. 20 mg GS-0976 treatment is capable of lowering hepatic steatosis, according to a phase II clinical research involving 126 patients with at least 18% hepatic steatosis (NCT02856555). In another open-label, proof-of-concept, phase II trial, fisocostat plus semaglutide, fisocostat plus cilofexor, or semaglutide monotherapy, could result in the greater capability of preventing hepatic steatosis and biochemical markers^528^(NCT03987074).

### Resveratrol (RES)

Resveratrol (RES) is an important phenolic phytochemical that may improve mitochondrial function and prevent metabolic diseases. PGC-1 activity and acetylation are reduced by RES, respectively.^529^ A phase III study in patients with diabetic nephropathy indicated that with the treatment of RES, the urine albumin/creatinine ratio was markedly decreased and serum creatinine was unchanged, while serum antioxidant enzymes were significantly increased (NCT02704494).^530^ Patients with T2DM and CHD who received resveratrol treatment for 4 weeks saw improvements in their glycemic control, HDL cholesterol levels, total cholesterol to HDL cholesterol ratio, TAC, and MDA levels.^531^, Trans-resveratrol and hesperidin improved insulin resistance, dyslipidemia, hyperglycemia, hypertension, and low-grade inflammation in obese subjects.^532^ Kantartzis et al. conducted the largest randomized, placebo-controlled clinical trial to investigate the 3-month impact of 150 mg/d resveratrol in 112 overweight or NAFLD patients. They found that resveratrol treatment improved the liver status and did not affect fat content or cardiometabolic parameters in humans.^533^ However, Fogacci et al. suggested that the earlier study was designed without addressing the dose-dependent effects and oral administration of resveratrol might lead to poor bioavailability.^533^ Furthermore, a trial demonstrated that patients with resveratrol (≥300 mg/day) uptake had a significant decrease in total cholesterol, blood pressure, and blood glucose.^534^ In a comprehensive review and meta-analysis, resveratrol’s effectiveness and safety in controlling lipid and glucose levels in people with T2DM were shown.^533^

### ASK1 inhibitor

Upstream control of p38/JNK signaling is provided by the enzyme apoptosis signal-regulating kinase 1 (ASK1). Oxidative stress, ER stress, and inflammatory signals can primarily activate ASK1. P38/JNK pathway is associated with numerous diseases; however, the application of P38/JNK inhibitors is limited due to their essential roles in cell survival and homeostasis.^535^ As a result, ASK1 seems to be a different therapeutic target for conditions and cell death caused by p38/JNK. The role of ASK1 in metabolic disorders has been demonstrated in mice, supporting clinical trials of ASK1 inhibitory compounds.

In 2016, a phase II clinical trial involving 333 diabetic nephropathy patients(NCT02177786) suggested that 18 mg selonsertib (GS-4997, an ASK1 inhibitor) treatment for 48 weeks showed a 71% reduction in eGFR.^536^ Another phase IIb clinical trial assessing the effect of selonsertib on diabetic kidney disease is still ongoing (NCT04026165).

In 2016, a phase II clinical trial involving 72 patients with NASH and stage 2-3 liver fibrosis (NCT02466516) showed that, 18 mg and 6 mg selonsertib-treated groups had a 43% and 30% reduction in fibrosis, respectively.^537^ In another IIb trial (NCT03449446), selonsertib combing firsocostat and cilofexor could effectively improve NASH activity and fibrosis.^538^

### PARP inhibitors

It has been discovered that the hydroximic acid derivative BGP-15 inhibits PARP1. BGP-15 can also prevent p38 MAPK/JNK activation and induce phosphorylation of Akt and GSK-3,^539^ increasing insulin sensitivity. In a phase II clinical trial including 47 non-diabetic patients with impaired glucose tolerance, the BGP-15 treatment group demonstrated enhanced insulin sensitivity, and the drug was safe and well-tolerated.^540^ However, a phase II experiment that examined the safety and effectiveness of BGP-15 in T2DM patients was stopped early for lack of findings (NCT01069965).

### FXR agonists

The Farnesoid X Receptor (FXR) is a member of the nuclear receptor family is a ligand-stimulated transcription factor. Many genes involved in the metabolism of bile acids, lipids, glucose, and amino acids are controlled by FXR. FXR may be phosphorylated, SUMOylated, and acetylated, and these modifications have an impact on the receptor functions.

The safety, tolerability, pharmacodynamics, and pharmacokinetics of tropifexor (LJN452) were studied in a clinical trial funded by Novartis Institutes for Biomedical Research. The results showed that in healthy subjects, tropifexor was well tolerated without altering plasma lipid levels, and had a pharmacokinetic profile suitable for once-daily administration.^541^ A phase II clinical trial enrolling 99 NASH patients showed that the decrease in total liver fibrosis was associated with the tropifexor therapy.^542^(NCT02855164).

In a phase II, double bind, placebo-control clinical trial with 140 noncirrhotic NASH patients, the 100 mg cilofexor (GS-9647) treatment group achieved a better result in NAFLD Activity Score (NAS), hepatic histology, and hepatic biochemistry^543^(NCT02854605). In two phase II co-medication clinical trials, cilofexor with semaglutide or firsocostat may be a better treatment strategy for NASH^528,538^ (NCT03449446, NCT03987074).

### Caspase inhibitors

Caspases, a class of cysteine-dependent aspartate-specific proteases, are crucial for maintaining cellular and organismal homeostasis by mediating inflammatory response and cell death. PTMs that control caspase modification, including phosphorylation, nitrosylation, ubiquitination, SUMOylation, glutathionylation, and acetylation, are essential for both cell survival and death.^544^

To date, several clinical trials of emricasan (an irreversible pan-caspase inhibitor) have shown improved biomarkers.^545^ However, in a randomized, placebo-controlled phase II trial, 318 NASH patients were administrated with 5 mg emricasan, 50 mg emricasan or placebo; emricasan did not improve liver ballooning and NASH fibrosis546 (NCT02686762).^546^ In another phase II double-blind, placebo-controlled clinical trial with 217 NASH patients, emricasan is safe but has a poor therapeutic effect in patients with cirrhosis^547^(NCT03205345).

### Glucokinase activators

Glucokinase (GCK) is one member of the hexokinase enzymes family and plays a critical role in glucose metabolism as a glucose-sensing. GCK regulates glucose homeostasis by catalyzing the phosphorylation of glucose into glucose-6 phosphate. GCK activity and cellular stability in beta cells are controlled by SUMOylation. S-nitrosylation of GCK at cysteine-371 promotes GCK enzymatic activity and increases insulin secretion. Glucokinase activators (GKAs), a particular class of T2DM medication, are created to target GCK. Several GKAs have recently been tested in clinical trials.

Dorzagliatin was the first GKA approved for T2DM in China. In a trial, 24 patients with T2DM were randomly assigned to receive dorzagliatin 75 mg once or twice daily for 28 days. Dorzagliatin displayed the capacity to improve β-cell function and regulate blood glucose. In a phase II, multicenter, randomized, double-blinded, and placebo-controlled clinical study enrolling 258 Chinese individuals with T2DM, dorzagliatin was safe and well tolerated, which finally improved glycemic control throughout 12 weeks^548^(NCT02561338). In another phase III clinical trial, dorzagliatin combined with metformin exhibited good glycemic management ability with safety^549^(NCT03141073).

In a phase III clinical study with 587 T2DM patients, MK-0941 (a new GKA) significantly reduced the levels of HbA1c and postmeal glucose in individuals that received conventional treatment of insulin glargine (metformin 1500 mg/day).^550^

AZD1656, another GKA, demonstrated good safety and tolerability in numerous ascending dose trials, and patients experienced lower fasting blood glucose levels.^551^ Moreover, the HbA1c and blood glucose levels were lowered by AZD1656 treatment in two different dose-range investigations^552,553^(NCT01152385; NCT01020123).

### PTP1B inhibitors

The first member of the protein tyrosine phosphatase (PTP) family and a suppressor of the leptin and insulin signaling pathways is PTP1B. PTP1B is abundantly expressed in tissues that respond to insulin, negatively regulating both integrin and insulin by dephosphorylation. PTP1B enzyme activity is inhibited by S-nitrosylation of Cys215. In diverse animal models, several PTP1B inhibitors have demonstrated their potential for treating numerous diseases, such as diabetes, obesity, and cancer. However, few PTP1B inhibitors are suitable for clinical application and the trials about PTP1B inhibitors went to termination because of their poor efficacy and safety. For example, trodusquemine (MSI-1436) was studied in 4 phase I clinical trials which were completed without results from 2007 to 2018 (NCT00509132; NCT00806338; NCT00606112; NCT02524951).

### HDAC inhibitors

HDAC, which removes the acetyl group from histone and non-histone proteins, is a novel molecular target in the treatment of type 2 diabetes and obesity. Clinical trials have looked into HDAC inhibitors.

In a clinical study with a double-blinded, randomized, placebo-controlled, crossover design, 18 T2DM patients accepted sodium phenylbutyrate (NaPB, an HDAC inhibitor) or a placebo. However, no difference was observed in the levels of insulin, triglycerides, FFA, or the amount of fat accumulating in muscle and the liver. The experiment indicated that NaPB increased peripheral insulin sensitivity when compared to placebo^554^(NTR7426). Another clinical trial involving 8 overweight or obese nondiabetic male subjects treated with phenylbutyrate (PBA) or placebo demonstrated that PBA prevented lipid-induced β-cell dysfunction.^555^

## Concluding remarks and future perspectives

### Novel PTMs in cardiometabolic diseases

In addition to the classic PTMs addressed in detail above, some novel PTMs emerge, such as succinylation, lactylation, beta-hydroxybutyryration, and lysine crotonylation. Next, we will describe the roles of some representative novel PTMs in metabolic diseases.

#### Succinylation and cardiometabolic disease

SIRT7 desuccinylates PRMT5, enhances PRMT5 activity, and induces SREBP1a arginine methylation. Arginine di-methylation of SREBP1a promotes the biogenesis of fatty acids, TGs and cholesterol.^556^ The data suggest that succinylation is a negative feedback regulator in cholesterol metabolism.^557^ Serum succinate levels were elevated in hyperlipidemia individuals and atherosclerotic mouse models,^558^ and succinate activates the succinate receptor 1 (SUCNR1)/renin-angiotensin system (RAS) pathway to increase Ang-II secretion, leading to endothelial cell damage.^559^ Succinate promotes VSMC proliferation and SMC migration. By the production of pro-inflammatory cytokines, succinate stimulates M1 polarization and causes atherosclerosis to form.^184^ Nine succinylation sites were identified in DIO mice.^560^ Increased protein succinylation has been identified in the muscle of type 1 diabetic rats and adipose tissue in type 2 diabetic mice.^561^ SIRT5-mediated desuccinylation of the K108 site of optineurin protects retinal ganglion cells in diabetic retinopathy.^562^ Succinated protein has been found in adipose tissue of DIO, *db/db*, and *ob/ob* mice.^561^ Supplementation with succinic acid protects mouse female offspring from HFD-induced obesity.^563^ Bioinformatics analysis showed that succinylation was the target of SIRT5, and liver overexpression of SIRT5 improved metabolic disorders in *ob/ob* mice. An earlier study using a mouse model of NAFLD caused by a high-fat, low-protein diet and carbon tetrachloride injection discovered substantial alterations in the liver’s succinylation levels.^183^

#### Lysine crotonylation and cardiometabolic disease

Lysine crotonylation is involved in cellular metabolism, cell cycle and tissue processes.^564^ Short-chain enyl-CoA hydrase (ECHS1) favors cardiac homeostasis by mediating crotonylation of histone proteins. Downregulation of ECHS1 increases histone crotonylation levels at H3K18 and H2BK12 and enhances the expression of genes associated with cardiac hypertrophy. The role of lysine crotonylation in metabolic diseases remains to be investigated. Improving crotonylation levels of oocytes can combat a decrease in oocyte quality in diabetics.^193^ Like acetylation, protein crotonylation is essential for controlling the browning of WAT. Acox2 is a lysine crotonylation regulator that controls the mice’s hepatic metabolic balance.^192^ It is still unknown how lysine crotonylation affects metabolic disorders.

#### Beta-hydroxybutyryration and cardiometabolic disease

Histone lysine β-hydroxybutyrylation (KbHb) is connected to upregulated genes in metabolic response pathways to starvation. Stimulation of ketone body production after starvation increases KbHb and lysine butyrylation.^565^ KbHb’s macronutrient pathway is enriched, including fatty acid β-oxidation, TCA cycle, glycolysis, amino acid metabolism, urea cycle, and ketogenesis.^566^ Elevated β-hydroxybutyric acid has been found to antagonize aortic endothelial damage and has a protective effect on diabetic vascular disease;^197^ at the same time, KbHb can inhibit the occurrence of kidney disease caused by diabetes.^567^ KbHb has the effect of improving obesity.^568^ Oral β-hydroxybutyric acid has been reported to improve blood lipid status by increasing HDL levels and reducing LDL/HDL ratios in Wistar rats.^198^ There is growing evidence that β-OHB is beneficial in vascular function and metabolism. In the Chinese sample, serum β-OHB levels were discovered to be related to coronary artery disease.^569^ Oral administration of ketone body 3-hydroxybutyrate (3-HB) can improve atherosclerosis in *ApoE*^-/-^ mice by inhibiting macrophage autophagy.^199^

#### Lactylation and cardiometabolic disease

Histone lactylation mediates repolarization of macrophages from M1 to M2 by transitioning from glycolysis to oxidative phosphorylation, thereby providing an anti-atherosclerotic effect.^204^ Elevated serum lactate levels were found to be significantly associated with plaque load and atherosclerosis in the Community Atherosclerosis Risk (ARIC) study cohort.^206^ In human skeletal muscle, lactic acid-induced lactation is correlated with insulin intolerance.^207^

### The association of novel protein modification sites suggested by high-sensitivity mass spectrometry

Mass spectrometry (MS) is a cutting-edge research tool for PTM analysis due to its advantages of excellent specificity and sensitivity. Numerous protein changes can be found using MS. With the improvement of analytical methods and mass spectrometry instruments, the methods for detecting PTMs are becoming increasingly comprehensive. Fourier transform (FT)-based mass spectrometers are characterized by high resolution and high sensitivity for PTM analysis. Discovery of structural mechanisms involving histone activity depends on crosslinked mass spectrometry (XL-MS).^570^ Using the LC-MS/MS method to assess the efficacy of the discovered modification sites, an MS/MS spectrogram can be produced. The isolation of target chromatin regions for the purpose of identifying histone posttranslational modifications is made easier by chromatin affinity purification using MS (ChAP-MS).^571^ The recently developed electron-transfer/higher-energy collision dissociation (ETHcD) has allowed for appreciable strides in addressing the uncertain locations of traditional fragmentation techniques in PTM omics.^572^ Affi-BAMS’ novel analytical immunoassay platform provides fast, highly specific quantitative data for protein and PTM targets. Accurate analysis of specific targets in complex proteomes is also possible.^573^ LC-FAIMS-MS/MS plays an important role in characterizing PTM crosstalk.^574^ Data-independent mass spectrometry techniques can identify precursor ion compliance with the modification, determine the quality and localization of peptide sequence modifications, and also record the peptide content in the sample, including the modified peptide. PTM site deletions can be detected in combination with LC-MS, isotope and antipeptide antibodies. LERLIC-MS/MS is used to study PTM modifications of complex biological samples in shotgun proteomics, which is directly coupled to tandem mass spectrometry (MS/MS). Broadband collision-induced dissociation (bbCID) mass spectrometry guide targeted proteomics, especially in the identification of glycopeptides and phosphopeptides.^575^ Low-intensity precursor ions can be used in liquid chromatography-electrospray ionization-quadrupole-time-of-flight mass spectrometry (LC-ESI-q-TOF MS/MS) to identify changed peptides.^576^

High-sensitivity mass spectrometry can efficiently and accurately identify metabolic disease-related proteins, and analyze the type and abundance of PTMs. For example, high-sensitivity mass spectrometry can aid diabetes risk assessment by analyzing amino acids, amines, and other polar metabolites, and the binding to tagging sites allows specific quantification of PTM sites.^577^ T2DM is linked with the type and site of PTM of hemoglobin, which may offer useful noninvasive biomarkers for protein damage in T2DM. The research of T2DM and other metabolic diseases is made possible by the detailed analysis of various sites in advanced glycosylation end products (AGEs).^578^ Mass spectrometry-based proteomics has discovered tens of thousands of PTM sites in the proteome. Proteomics techniques can measure and quantify PTMs that co-occur on the proteome or the same protein. At the same time, examining the steady-state levels of histone PTM in cells under various perturbations makes it possible to investigate the crosstalk between PTMs and its functional significance. Research on histone and nucleoproteins has recently benefited from the integration of proteomics based on high-resolution mass spectrometry and isolation of acetylated peptides.^579^ The latest PTM detection methods above have opened up the area of PTMs and their role in metabolic and cardiovascular diseases.

### The crosstalk of different PTM omics

The interaction between multiple PTMs in a protein to jointly regulate the biochemical function of a protein through positive and negative regulatory interactions is called PTM crosstalk. Positive and negative crosstalk are two categories of crosstalk, depending on how the PTMs interact with each other. When multiple modifications of the same or distinct kinds are present in the same sequence area (within five amino acids), but do not impact the same residues, this is referred to as positive crosstalk. These changes could take place concurrently, or they could have a causal or chronological connection. Negative crosstalk occurs when two PTMs directly fight for the same amino acid, when one PTM obscures the recognition site of another PTM, or when PTMs “compete” for the same residue in a causal or temporal way.^580^ In important protein domains like histones, protein kinases, and RNA recognition motifs, which are involved in a variety of biological processes like RNA processing, DNA damage response, signal transduction, and cell cycle regulation, crosstalk between multiple PTMs happens more frequently.^581^

There is increasing evidence that PTM crosstalk is associated with the occurrence and development of various diseases, including metabolic diseases. Studies have suggested that interactions between O-GlcNAcylation and other PTMs may be involved in obesity and diabetes, and glucose toxicity may promote metabolic syndrome through abnormal O-GlcNAcylation and ubiquitination of these proteins in various tissues. Crosstalk between O-GlcNAcylation and phosphorylation initiated by insulin signaling is associated with cytoplasmic enzyme activity that regulates eNOS during vasodilation, and kinetic dysregulation between O-GlcNAcylation and eNOS phosphorylation leads to early endothelial dysfunction in diabetes-accelerated atherosclerosis.^582^ O-GlcNAc acylation in the insulin signaling pathway, which is the changed central pathway in diabetes, can prevent phosphorylation of the site and impair this signaling pathway.^583^ PTM crosstalk shows different mechanisms in different types of diseases, whereas in situ crosstalk may drive differential interactions in CVDs. p53 is a classic example of non-histone PTM crosstalk. By directly competing with the ubiquitination of the same residue, which is carried out by MDM2, p53’s CTD lysine acetylation inhibits ubiquitin-proteasome-dependent breakdown. We can better comprehend the etiology of diseases and identify novel targets for drug treatment with a thorough knowledge of PTM crosstalk.^584^

### The superiority of targeting PTMs

Various PTMs play a role in almost every part of biological processes, and abnormal PTM states are frequently linked to human illnesses. A method for natural protein breakdown known as proteolysis-targeting chimera (PROTAC) successfully ubiquitinates target proteins using the in vivo UPS.^585^ A new medicinal approach to drug finding is using PROTAC-targeted protein degradation. The PROTAC complex consists of the E3 ligase and the target protein. In addition to offering novel chemical knockdown instruments for biological study, PROTACs have the potential to be used as therapeutic therapies for illnesses like obesity, hyperlipidemia, cancer, immune disorders, virus infections, and neurodegenerative diseases. PROTACs are divided into PROTACs that target protein kinases,^586^ nuclear receptors, and transcriptional regulators. In traditional cancer treatment, gene editing technology is used to remove cancer-related pathogenic proteins, such as CRISPR-Cas9 technology, genetic change through RNA interference, transcription activator-like effector nucleases, and recombination-based gene deletion. The expensive and time-consuming nature of conventional genome editing methods, especially in large nonhuman primates, has caused many inconveniences to researchers. In addition, some uncertainties, such as spontaneous mutations in knockout models, potential genetic compensation, and embryonic lethality, may lead to failure. PROTAC offers a potential strategy to knock down proteins of interest rapidly and reversibly, which is not possible with genome editing strategies.^587^ The advantages of PROTACs include event-driven activity, targeting undruggable proteins, overcoming traditional drug resistance, and other effects. Statistics show that more than 80% of human proteins are unreachable targets.^588^ Since POI ligands, E3 ubiquitin ligases, and linkers work together synergistically, and the ternary complex has a rigid shape, PROTAC molecules show the advantage of high selectivity for target proteins, which can convert non-druggable targets into druggable targets. PROTAC has the advantage of targeting non-druggable proteins.^589^ Current research on PROTACs is more focused on the treatment of cancer.

A method to achieve extensive substrate dephosphorylation has been developed, called phosphorylation targetan mosaicism (PhosTACs). Unlike PROTACs, PhosTAC focuses on recruiting Ser/Thr phosphatase into phosphate matrices to mediate its dephosphorylation, which can uniquely provide targeted function gain opportunities to manipulate protein activity; this mode of action provides PROTAC with greater selectivity, extended period of effect, and the ability to overcome drug resistance. PhosTAC works similarly to PROTAC and has great potential both in terms of biological tools and clinical treatment.^590^

In recent years, studies have found that PNPLA3 levels are lowered by PROTAC-mediated degradation, reducing liver TG content in PNPLA3-overexpressing (148 M) mice.^591^ An oral active VHL recruitment PROTAC (21c) can lead to HMGCR degradation as well as cholesterol reduction, which offers fresh methods for treating hyperlipidemia and associated metabolic illnesses.^592^ Lycoline binds directly to SCAP to inhibit SREBF, thereby improving HFD-induced obesity, hyperlipidemia and insulin resistance.^593^

### Development of PTM database and research tools

In numerous biological functions, protein PTMs are crucial.^594^ PTM is closely linked to the onset and progression of many illnesses, including metabolic illnesses like obesity, diabetes, hypertension, and hyperlipidemia. Understanding basic biological processes and the frequency of diseases is significantly affected by PTM analysis. The PTM database is a database for researchers to study the protein structure of multiple species, their association with diseases, PTM sites, the relationship between enzymes and substrates, the relationship between substrates and PTM sites, protein interactions, cell signaling, and transcription. Information on regulation and PTM site prediction provides important information for the majority of researchers. Table 2 lists the main PTM databases.

**Table 2:** PTM resource database

No.	Short Name	DB Name	URLs
1	dbPTM	A database for exploring regulatory networks and functional associations of protein posttranslational modifications	https://awi.cuhk.edu.cn/dbPTM/
2	PTMcode v2	A resource for functional associations of posttranslational modifications within and between proteins	http://ptmcode.embl.de
3	PTM-SD	A database of structurally resolved and annotated posttranslational modifications in proteins	http://www.dsimb.inserm.fr/dsimb_tools/PTM-SD/
4	FAT-PTM	The Functional Analysis Tools for Posttranslational Modifications (FAT-PTM) database: a PTM database for analysis of proteins and metabolic pathways	https://bioinformatics.cse.unr.edu/fat-ptm/
5	PTMD	A Database of Human Disease-associated Posttranslational Modifications	http://ptmd.biocuckoo.org
6	DEPOD	The Human DEPhOsphorylation Database	http://depod.bioss.uni-freiburg.de/
7	iPTMnet	An integrated resource for protein post-translational modification network discovery	http://proteininformationresource.org/iPTMnet
8	PhosPhAt 4.0	The Arabidopsis Protein Phosphorylation Site Database: an updated arabidopsis database for searching phosphorylation sites and kinase-target interactions	http://phosphat.uni-hohenheim.de
9	Phospho.ELM	A database of phosphorylation sites	http://phospho.elm.eu.org
10	PhosphoGrid	A database of experimentally verified in vivo protein phosphorylation sites from the budding yeast saccharomyces cerevisiae	http://www.phosphogrid.org
11	PhosphoSitePlus	A knowledgebase dedicated to mammalian post-translational modifications (PTMs)	http://www.phosphosite.org
12	UniCarbKB	A new database features for integrating glycan structure abundance, compositional glycoproteomics data, and disease associations	http://www.unicarbkb.org/
13	VPTMdb	A viral posttranslational modification database	http://vptmdb.com:8787/VPTMdb/
14	AWESOME	A database of SNPs that affect protein posttranslational modifications	https://github.com/mgramin/awesome-db-tools
15	PTM-Switchboard	A database of posttranslational modifications of transcription factors, the mediating enzymes and target genes	http://cagr.pcbi.upenn.edu/PTMswitchboard
16	PTM-Shepherd	Analysis and Summarization of PostTranslational and Chemical Modifications From Open Search Results	https://github.com/Nesvilab/PTM-Shepherd
17	iProteinDB	An Integrative Database of Drosophila Post-translational Modifications	https://www.flyrnai.org/tools/iproteindb/web/
18	PRISMOID	A comprehensive 3D structure database for posttranslational modifications and mutations with functional impact	https://prism.erc.monash.edu/
19	dbGSH	A database of S-glutathionylation	http://csb.cse.yzu.edu.tw/dbGSH/
20	qPTMplants	An integrative database of quantitative posttranslational modifications in plants	http://qptmplants.omicsbio.info
21	novPTmenzy	A database for enzymes involved in novel posttranslational modifications	http://www.nii.ac.in/novptmenzy.html
22	SysPTM 2.0	An updated systematic resource for posttranslational modification	http://lifecenter.sgst.cn/SysPTM/
23	PHOSIDA	A posttranslational modification database	http://www.phosida.com
24	BioGRID	A comprehensive biomedical resource of curated protein, genetic, and chemical interactions	https://thebiogrid.org
25	qPhos	A database of protein phosphorylation dynamics in humans	http://qphos.cancerbio.info
26	EPSD	Eukaryotic Phosphorylation Site Database: a well-annotated data resource of protein phosphorylation sites in eukaryotes	http://epsd.biocuckoo.cn/
27	CPLM 4.0	An updated database with rich annotations for protein lysine modifications	http://cplm.biocuckoo.cn/
28	YAAM	Yeast Amino Acid Modifications Database	http://yaam.ifc.unam.mx/
29	HPRD	Human Protein Reference Database	http://www.hprd.org/
30	Phospho3D 2.0	An enhanced database of three-dimensional structures of phosphorylation sites	http://www.phospho3d.org/
31	LymPHOS 2.0	An update of a phosphosite database of primary human T cells	http://www.lymphos.org
32	P3DB 3.0	From plant phosphorylation sites to protein networks	http://p3db.org
33	UniPep	A database for human N-linked glycosites: a resource for biomarker discovery	http://www.unipep.org
34	GlycoFish	A database of zebrafish N-linked glycoproteins identified using SPEG method coupled with LC/MS	http://betenbaugh.jhu.edu/GlycoFish
35	mUbiSiDa	A comprehensive database for protein ubiquitination sites in mammals	http://reprod.njmu.edu.cn/mUbiSiDa
36	SwissPalm	A database on protein S-palmitoylation	https://swisspalm.org/

**Fig. 8:**
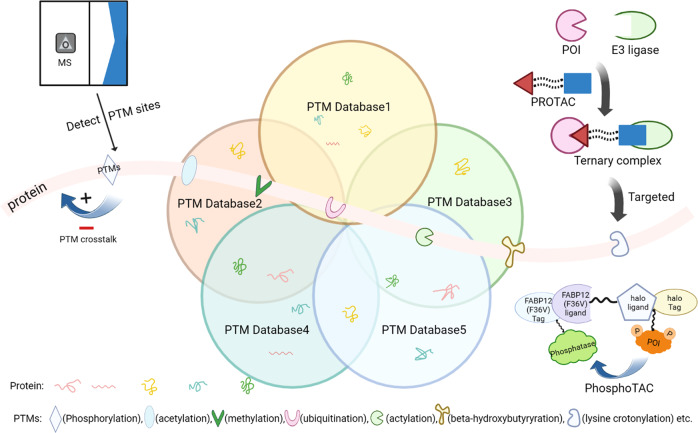
Future outlook of PTMs research. To date, there are several PTM databases, and each one contains thousands of proteins and overlapping parts. PTM crosstalk regulates multiple PTMs on the same or different protein substrates. Mass spectrometry (MS) is a commonly used tool to study PTMs, and MS-based different PTM omics indicate the potential crosstalk. Recently, the proteolysis-targeted chimerism (PROTAC) technology for targeted protein degradation is an innovative strategy to treat various diseases. PhosTAC focuses on recruiting Ser/Thr phosphatase into phosphate matrices to mediate, which can uniquely provide targeted function gain opportunities to manipulate protein activity. PhosTAC works similarly to PROTAC and has great potential as biological and pharmacological tools. The figure is generated with BioRender (https://biorender.com)

Classical methods for the detection of protein PTMs include specific antibody development, radioisotope-labeled substrates, western blot analysis, chemically tagged peptides,^595^ and PTM-specific enzymes by peptides. The advancement of new biotechnologies to examine the landscape of different PTMs in various metabolic diseases will lead to the identification of new drug candidates. Figure 8 describes the crosstalk of PTMs, MS detection methods, PROATC, and PTM database.

In summary, PTMs control target protein function and stability, protein-protein interactions, and subcellular localization, contributing to almost all biological processes, thus providing novel and diverse mechanisms for the regulation of systematic biology. Targeting PTMs presents an opportunity for a novel or refined drug development that could provide a novel and more precise approach for the management of metabolic diseases.

## Supplementary information

Supplementary Materials for Targeting protein modifications in metabolic diseases: molecular mechanisms and targeted therapies
