# Supplementary material for: Targeting protein modifications in metabolic diseases: molecular mechanisms and targeted therapies
Source: Signal Transduct Target Ther. 2023 May 27;8:220. doi: 10.1038/s41392-023-01439-y (PMC10224996; doi:10.1038/s41392-023-01439-y)
Supplement: Supplementary file 1 — Supplementary Materials for Targeting protein modifications in metabolic diseases: molecular mechanisms and targeted therapies [file 41392_2023_1439_MOESM1_ESM.docx]

Supplementary Materials for

Targeting protein modifications in metabolic diseases:

molecular mechanisms and targeted therapies

Xiumei Wu, Mengyun Xu, Mengya Geng, Shuo Chen, Peter J. Little, Suowen Xu, Jianping Weng*

Correspondence to: wengjp@ustc.edu.cn

Xiumei Wu and Mengyun Xu contribute equally to this work.

Suowen Xu and Jianping Weng are co-senior authors.

**This file includes:** Supplementary Table 1

Supplementary Table 1. Pharmaceutical Intervention of protein modification in preclinical studies of metabolic diseases

| No. | | Compound | | | | | | Functions | | | | | Animal model | | | | | Dose and duration | | | | | | Observed effects | | | | Ref. | | |
| --- | --- | --- | --- | --- | --- | --- | --- | --- | --- | --- | --- | --- | --- | --- | --- | --- | --- | --- | --- | --- | --- | --- | --- | --- | --- | --- | --- | --- | --- | --- |
| 1.1 Phosphorylation in diabetes | | | | | | | | | | | | | | | | | | | | | | | | | | | | | | |
| 1 | | Metformin | | | | | | AMPK activator | | | | | Sprague Dawley (SD) rats | | | | | 100 mg/ml once or twice a day for 5 days | | | | | | ↓β-hydroxybutyrate; ↓hepatic expression of SREBP-1, FAS, and S14; ↓hepatic glucose production; ↑phosphorylation and activation of AMPK | | | | ^1^ | | |
| 2 | | Fasudil | | | | | | Protein kinases inhibitor | | | | | STZ-induced SD rats | | | | | 10 mg/kg/d for 8 weeks | | | | | | ↓p-cofilin; ↓PKCβ2 phosphorylation at Thr641 and Ser660 | | | | ^2^ | | |
| 3 | | Indole-5-carboxamide (SD-169) | | | | | | p38α MAPK inhibitor | | | | | NOD mice | | | | | 200 or 600 mg/kg/week for 10 weeks | | | | | | ↓incidence of diabetes; ↓p38 and HSP60 in T cells of the pancreatic beta islets; ↓p38 MAPK signaling pathway | | | | ^3^ | | |
| 4 | | KY-226 | | | | | | Protein tyrosine phosphatase 1B (PTP1B) inhibitor | | | | | C57BL/6 mice;  *db/db* mice | | | | | 30 and 60 mg/kg/d for 4 weeks | | | | | | ↓blood HbA1c values, plasma insulin levels; ↑STAT3 phosphorylated by leptin in the hypothalamus | | | | ^4^ | | |
| 5 | | U0126 | | | | | | MEK1 and MEK2 inhibitor | | | | | STZ-induced C57BL/6 mice | | | | | 1 mg/kg/d for 8 weeks | | | | | | ↓ERK1/2 phosphorylation; ↓XBP1s Ser 348 phosphorylation; ↓blood glucose in DCM mice | | | | ^5^ | | |
| 6 | | TDZD-8 | | | | | | GSK-3β Inhibitor | | | | | C57BL/6 mice;  *db/db* mice | | | | | 2 μM/24h for 2 weeks | | | | | | ↓dephosphorylation and activation of GSK3β; ↓ tau phosphorylation at multiple epitopes in db/db mice; ↓ obesity and diabetes in db/db mice | | | | ^6^ | | |
| 7 | | S3I- 201 and erlotinib | | | | | | EGFR tyrosine kinase inhibitor | | | | | C57BL/6 mice | | | | | S3I-201 (5 mg/kg) or  Erlotinib (10mg/kg) every other day for 16 weeks | | | | | | ↓hyperglycemia; ↓STAT3 phosphorylation; ↓cardiomyopathy | | | | ^7^ | | |
| 8 | | Y396 | | | | | | EGFR tyrosine kinase inhibitor | | | | | SD rats;  C57BL/6 mice | | | | | 20 mg/kg every other day for 4 weeks | | | | | | ↓high glucose induced-phosphorylation of EGFR; ↓blood glucose; ↓P-EGFR, p-AKT, p-eIF2α, CHOP, ATF6, and Nox4; ↓body weight in T1DM; ↑survival rate | | | | ^8^ | | |
| 9 | | 5-aminoimidazole-4-carboxamide-1-d-ribofuranoside (AICAR) | | | | | | AMPK activator | | | | | *db/db* mice | | | | | 0.25 g/kg once | | | | | | ↓blood glucose levels; ↓the phosphorylation of ERK in adipose tissue | | | | ^9^ | | |
| 10 | | AG1478 | | | | | | EGFR tyrosine kinase inhibitor | | | | | C57BL/6 mice | | | | | 10 mg/kg/day for 2 weeks | | | | | | ↓body weight; ↓blood glucose level; ↓the phosphorylation of EGFR and eIF2α | | | | ^10^ | | |
| 11 | | JTT-551 | | | | | | Protein tyrosine phosphatase 1B (PTP1B) inhibitor | | | | | *ob/ob* mice;  *db/db* mice | | | | | *ob/ob*: 0.1 or 1 or 10 mg/kg/d for 7 days; *db/db*: 3 or 30 mg/kg for 4 weeks | | | | | | ↓ insulin level, body weight; ↑autophosphorylation of Irβ; ↑glucose and lipid metabolism | | | | ^11^ | | |
| 12 | | SB 203580 | | | | | | p38α MAPK inhibitor | | | | | C57BL/6 mice | | | | | 1 mg/kg/d for 8 weeks | | | | | | ↓phosphorylation of the p38 MAPK; ↓the levels of IL6 in the cardiac tissue; ↑LV function in STZ-induced diabetic cardiomyopathy | | | | ^12^ | | |
| 1.2 Acetylation in diabetes | | | | | | | | | | | | | | | | | | | | | | | | | | | | | | |
| 13 | | Trichostatin A | | | | | | HDAC class I/II inhibitor | | | | | SD rats | | | | | 500 μg/kg/d for 4 weeks | | | | | | ↓proteinuria and extracellular matrix production; ↑acetylation of histones H3 and H4 | | | | | ^13^ | |
| 14 | | Vorinostat | | | | | | Pan-inhibitor of HDAC1, HDAC2, HDAC3, HDAC6, HDAC7 and HDAC11 | | | | | SD rats | | | | | 20 or 50 mg/kg/d for 4 weeks | | | | | | ↓ the EGFR in the kidneys of diabetic rats; ↓early diabetic renal enlargement; ↓renal growth and glomerular hypertrophy | | | | | ^14^ | |
| 15 | | Vorinostat | | | | | | Pan-inhibitor of HDAC1, HDAC2, HDAC3, HDAC6, HDAC7 and HDAC11 | | | | | C57BL/6 mice;  *eNOS*^-/-^ mice | | | | | 50 mg/kg/d for 18 weeks | | | | | | ↓Renal Injury in STZ-diabetic C57BL/6 mice; ↓eNOS Expression in the Kidneys; ↓oxidative stress, albuminuria and glomerular matrix production in STZ-diabetic wildtype mice | | | | | ^15^ | |
| 16 | | Valproate | | | | | | HDAC1 inhibitor | | | | | Wistar rats | | | | | 200 mg/kg/d for 6 weeks | | | | | | ↓renal cell apoptosis; ↓the acetylation level of histone H4 in the promoter of CHOP; ↑the acetylation level of histone H4 in the promoter of GRP78 | | | | | ^16^ | |
| 17 | | Sodium butyrate (NaB) | | | | | | NMDA receptor antagonist | | | | | SD rats | | | | | 500 mg/kg/d for 21 days | | | | | | ↓plasma glucose and NF-κB expression; ↓the plasma creatinine and urea levels; ↓decreased the expression of iNOS; ↓the diabetes-induced over expression of collagen I in glomerular region; ↑the diabetes-induced decreased in the acetylation of histone H3 | | | | | ^17^ | |
| 18 | | Sodium valproate | | | | | | NMDA receptor antagonist | | | | | SD rats | | | | | 300 mg/kg/day for 8 weeks | | | | | | ↓ the fibroblast activation in diabetic kidney; ↓decreased the diabetes-induced over expression of COX-2 and ICAM-1; ↑diabetes-associated decreased acetylation of histone H3; ↑the reduction in body weight, anti-fibrotic and renoprotective effect | | | | | ^18^ | |
| 19 | | MGCD0103 | | | | | | HDAC (Class I/IV) inhibitor | | | | | SD rats | | | | | 10 ug/d for 4 weeks | | | | | | ↓STZ-induced hyperglycemia and inflammation in pancreatic islets; ↑SOD expression and histone acetylation level on promoters; | | | | | ^19^ | |
| 1.3 Methylation in diabetes | | | | | | | | | | | | | | | | | | | | | | | | | | | | | | |
| 20 | GSK-J4 | | | | | | H3K27me3/me2-demethylases JMJD3/KDM6B inhibitor | | | | | | | | | | C57BL/6 mice;  *db/db* mice | | | | | 10,50,100 mg/kg twice per day for 8 days | | | ↓diabetes‐induced DNA damage on renal sections of db/db mice; ↑renal H3K27me3 levels | | | | ^20^ | |
| 1.4 Ubiquitination in diabetes | | | | | | | | | | | | | | | | | | | | | | | | | | | | | | |
| 21 | | MG132 | | | | | | Proteasome inhibitor | | | | | Wistar rats | | | | | 0.05 or 0.1 mg/kg/d for 8 weeks | | | | | | ↓kidney damage; ↓Smad7 ubiquitin degradation and TGF-β activation in DN | | | | | ^21^ | |
| 22 | | MF-094 | | | | | | USP30 inhibitor | | | | | SD rats | | | | | 50 mg/kg/d for 10 days | | | | | | ↓USP30; ↓NLRP3 and caspase-1 p20; ↓concentrations of IL-1β and IL-18 | | | | | ^22^ | |
| 1.5 Neddylation in diabetes | | | | | | | | | | | | | | | | | | | | | | | | | | | | | | |
| 23 | | | | MLN4924 (Pevonedistat) | | | | | | NEDD8-activating enzyme (NAE) inhibitor | | | | | | C57BL/6 mice | | | 60 mg/kg  once | | | | | ↓hepatic glucose production; ↓hyperglycemia in mice; ↑hepatic insulin signaling through insulin receptor substrate stabilization | | | | ^23^ | | |
| 1.6 ADP-ribosylation in diabetes | | | | | | | | | | | | | | | | | | | | | | | | | | | | | | |
| 24 | | | | PJ34 | | | | | | PARP-l/2 inhibitor | | | | | | *db/db* mice | | | | | | 3 doses | | ↓PARP activity, GAPDH ribosylation; GAPDH translocation; ↓muscle fiber injury; ↑metabolic activity following hind limb IR injury in a murine model of type-II diabetes | | | | ^24^ | |  |
| 2.1 Phosphorylation in obesity | | | | | | | | | | | | | | | | | | | | | | | | | | | | | |  |
| 25 | | KY-226 | | | | | | | | Protein tyrosine phosphatase 1B (PTP1B) inhibitor | | | | | | C57BL/6J mice;  *db/db* mice | | | | | | 10 or 30 mg/kg/d for 4 weeks | | ↓body weight gain in HFD-mice; ↓total food consumption and visceral fat volume gain at 60 mg/kg/day; ↑STAT3 phosphorylated by leptin in the hypothalamus; ↑p-IR and p-Akt in the liver and femoral muscle | | | | ^4^ | |  |
| 26 | | DPM-1001 | | | | | | | | Protein tyrosine phosphatase 1B (PTP1B) inhibitor | | | | | | C57BL/6 mice | | | | | | 5 mg/kg/d for 50 days | | ↓diet-induced obesity; ↑insulin and leptin signaling in high-fat diet-fed mice; ↑β-subunit phosphorylation | | | | ^25^ | |  |
| 27 | | Salubrinal | | | | | | | | eIF2α dephosphorylation inhibitor | | | | | | C57BL/6 mice | | | | | | 1 mg/kg/d for 8 weeks | | ↓body fat; ↓the mass of adipocyte tissue; ↓obesity-induced hepatic steatosis; ↓hepatic ER stress; ↑autophagy by maintaining eIF2α phosphorylation | | | | ^26^ | |  |
| 28 | | Amlexanox | | | | | | | | IKKε and TBK1 inhibitor | | | | | | C57BL/6 mice | | | | | | 25 mg/kg/d for 4 days | | ↑the phosphorylation of TBK1 at Ser172 and HSL phosphorylation; ↑catecholamine resistance in obese adipose tissue | | | | ^27^ | |  |
| 29 | | Sodium Butyrate | | | | | | | | NMDA receptor antagonist | | | | | | CD-1 mice | | | | | | 1% w/v for 12 weeks | | ↓HFD induced obesity and hypercholesterolemia | | | | ^28^ | |  |
| 30 | | Trodusquemine (MSI-1436) | | | | | | | | Protein-tyrosine phosphatase 1B (PTP1B) | | | | | | AKR/J mice | | | | | | Initial dose of 10 mg/kg with three subsequent weekly doses of 5 mg/kg for 22 days | | ↓appetite; ↓protein-tyrosine phosphatase 1B (PTP1B); ↓body weight in a fat-specific manner; ↑plasma insulin and leptin levels in mice | | | | ^29^ | |  |
| 31 | | Ampkinone | | | | | | | | AMP-activated protein kinase (AMPK) activator | | | | | | C57BL/6 mice | | | | | | 10 mg/kg/d for 1 month | | ↓total body weight and overall fat mass; ↑glucose dispersal and insulin sensitivity via activation of AMPK in vivo | | | | ^30^ | |  |
| 2.2 Acetylation in obesity | | | | | | | | | | | | | | | | | | | | | | | | | | | | | | |
| 32 | | | | Vorinostat (SAHA) | | | | | Pan-inhibitor of HDAC1, HDAC2 and HDAC3 (Class I), HDAC6 and HDAC7 (Class II) and HDAC11 (Class IV) | | | | | | C57BL/6 mice | | | | | | 50 mg/kg/d for 20 days | | ↑white adipose tissue browning in lean mice;  ↑energy expenditure and improves glucose homeostasis in obese mice | | | | ^31^ | | | |
| 33 | | | | Vorinostat (SAHA) | | | | | Pan-inhibitor of HDAC1, HDAC2 and HDAC3 (Class I), HDAC6 and HDAC7 (Class II) and HDAC11 (Class IV) | | | | | | Swiss albino mice | | | | | | 25 or 50 mg/kg/d for 2 weeks | | ↓HDL-cholesterol in HFD fed mice; ↓TNF-α level; ↓the histone H3 acetylation levels | | | | ^32^ | | | |
| 34 | | | | Tubastatin ACAY10603 and ricolinostat | | | | | HDAC6 inhibitor | | | | | | C57BL/6 mice | | | | | | 25 μL once | | ↓body weight and the plasma leptin level;  ↓HDAC6 deacetylase activity in the iWAT and BAT; ↑elevated Chil3 (also called Ym1) and Arg1 levels | | | | ^33^ | | | |
| 35 | | | | Trichostatin A | | | | | HDAC class I/II inhibitor | | | | | | C57BL/6 mice | | | | | | 1 or 2 mg/kg/d for 10 weeks | | ↓the weight of visceral adipocyte tissue and the bodyweight of HFD-fed obese mice | | | | ^34^ | | | |
| 36 | | | | MS-275 | | | | | HDAC class I inhibitor | | | | | | C57BL/6 mice | | | | | | 10 mg/kg every other day for 22 days | | ↓body weight, adipocyte size; ↑glucose tolerance; ↑thermogenic capacity; ↑expression of UCP 1 | | | | ^35^ | | | |
| 37 | | | | MS-275 | | | | | HDAC class I inhibitor | | | | | | C57BL/6 mice | | | | | | 5 mg/kg every alternate day for 7 weeks | | ↓calorie uptake; ↓body weight gain; ↓food uptake; ↓body weight gain in DIO mice; ↓ visceral adiposity; ↑energy expenditure | | | | ^36^ | | | |
| 38 | | | | CG200745 | | | | | Pan-HDAC inhibitor | | | | | | C57BL/6 mice | | | | | | 0.2 mg/kg/d for 9 days | | ↓HFD-induced body weight gain and hypertension; ↓HFD-induced increase in HDAC activity and expression in kidney | | | | ^37^ | | | |
| 39 | | | | Dacinostat (HY-13606) | | | | | HDAC1 inhibitor | | | | | | C57BL/6 mice | | | | | | 1 mg/kg/2d for 9 weeks | | ↓HFD-induced weight gain; ↓adipose tissue hypertrophy; ↑adipose thermogenesis; ↑adipose thermogenesis via transcriptional activation of Ucp1 and Ppargc1α by acetylation of histone 3 lysine 27 | | | | ^38^ | | | |
| 40 | | | | Scriptaid | | | | | HDAC inhibitor | | | | | | C57BL/6 mice | | | | | | 1 mg/kg/d for 4 weeks | | ↓lipids in skeletal muscle; ↓heart weight, body weight; ↑fatty acid oxidation; ↑lipid oxidation; | | | | ^39^ | | | |
| 41 | | | | Trichostatin A | | | | | HDAC class I/II inhibitor | | | | | | C57BL/6 mice | | | | | | 10 µg/kg/d for 2 weeks | | ↑PPARγ acetylation at multiple lysine sites (K289, K386, K462 and K466); ↑adipogenesis; ↑insulin sensitivity | | | | ^40^ | | | |
| 2.3 Methylation in obesity | | | | | | | | | | | | | | | | | | | | | | | | | | | | | | |
| 42 | | | | BIX01294 (BIX) and A366 | | | | | G9a and GLP histone methyltransferase inhibitor | | | | | C57BL/6 mice | | | | | | | BIX (50 mg/kg/d) or A366 (2 mg/kg/d) for 2 days | | ↓H3K9me2 levels; ↑musclin levels | | | | ^41^ | | | |
| 43 | | | | GSK126 | | | | | EZH2 methyltransferase inhibitor | | | | | C57BL/6 mice | | | | | | | 50 mg/kg/d for 10 days | | ↓the obese phenotype; ↓white adipogenesis; ↓H3K27me3; ↑lipolysis; ↑glucose tolerance | | | | ^42^ | | | |
| 44 | | | | GSK-J4 | | | | | H3K27me3/me2-demethylases JMJD3/KDM6B inhibitor | | | | | *ob/ob* mice | | | | | | | 30 mg/kg/d for 21 days | | ↓the body weight and food intake of DIO mice; ↑improved glucose homeostasis in DIO mice; ↑the H3k27me3 modification in the hypothalamus | | | | ^43^ | | | |
| 2.4 Ubiquitination in obesity | | | | | | | | | | | | | | | | | | | | | | | | | | | | | | |
| 45 | | | | GSK2643943A | | | | | Deubiquitinating enzyme (DUB) inhibitor | | | C57BL/6 mice | | | | | | | | | 30 mg/kg/d for 13 days | | ↓the USP20 hydrolyzed K48- and K63-linkages and reduced HMGCR ubiquitination; ↓cholesterol biosynthesis; ↓serum lipid contents; ↑glucose clearance; ↑succinate levels and energy expenditure | | | | ^44^ | | | |
| 2.5 Neddylation in obesity | | | | | | | | | | | | | | | | | | | | | | | | | | | | | | |
| 46 | | | | MLN4924 | | NEDD8-activating enzyme (NAE) inhibitor | | | | | C57BL/6 mice | | | | | | | | | | 30 mg/kg for a week | | ↓obesity and glucose intolerance in young mice; ↓PPARγ induced in HFD-fed mice; ↓PPARγ induced neddylation | | | | ^45^ | | | |
| 3.1 Phosphorylation in fatty liver disease | | | | | | | | | | | | | | | | | | | | | | | | | | | | | | |
| 47 | | | | LB100 | | PP2A inhibitor | | | | | C57BL/6 mice | | | | | | | | | | 1.5 mg/kg three times a week for 6 weeks | | ↓hepatic steatosis; ↓PP2A activity; ↑insulin sensitivity in HFD-fed mice; ↑the AMPK/Sirt1 pathway in vivo | | | | ^46^ | | | |
| 48 | | | | Udenafil | | PDE5 inhibitor | | | | | *ob/ob* mice | | | | | | | | | | 10 or 30 mg/kg/d for 6 weeks | | ↓inflammatory response and macrophage infiltration; ↑markers of steatohepatitis and regulates lipid metabolism in ob/ob mice; ↑lipolysis and beta oxidation in liver tissue | | | | ^47^ | | | |
| 49 | | | | Salubrinal | | eIF2α dephosphorylation inhibitor | | | | | C57BL/6 mice | | | | | | | | | | 1 mg/kg/d for 8 weeks | | ↓obesity-induced hepatic steatosis; ↓hepatic ER stress and promoted autophagy by maintaining eIF2α phosphorylation; ↑ obesity-induced hepatic fibrosis | | | | ^26^ | | | |
| 50 | | | | Dasatinib | | Dual Src/Bcr-Abl inhibitor | | | | | C57BL/6 mice | | | | | | | | | | 4 or 8 mg/kg/d for 4 weeks | | ↓the WD-induced disturbance of serum ALT, lipid profile; ↓hepatic expression of PDGFA, phosphorylated PDGFR α and β, IL1α, COX2, SREBP-1, iNOS, CD68, TGF-β and ASMA; ↓the steatosis, inflammation, hepatocellular ballooning, hepatic fibrosis and the high NAFLD activity scoring induced by WD; ↑expression for arginase-1 and CD163 | | | | ^48^ | | | |
| 51 | | | | GW501516 | | PPARδ agonists | | | | | C57BL/6 mice | | | | | | | | | | 3 mg/kg/d for 3 weeks | | ↓hepatic caspase-1 and IL-1β; ↓fatty acid-induced inflammation and steatosis; ↑the levels of AMPKα phosphorylation | | | | ^49^ | | | |
| 52 | | | | Gemigliptin | | Dipeptidyl peptidase-4 (DPP-4) inhibitor | | | | | C57BL/6 mice | | | | | | | | | | 330-380 mg/kg/d for 12 weeks | | ↓LECT2 expression; ↓hepatic dysfunction; ↓phosphorylated mTOR and cleaved SREBP-1; ↑Akt phosphorylation | | | | ^50^ | | | |
| 53 | | | | Ezetimibe | | Nrf2 activator | | | | | C57BL/6 mice | | | | | | | | | | 10 mg/kg/d for 4 weeks | | ↓hepatic steatosis, inflammation, and fibrosis in diet-induced NASH; ↓oxidative liver injury by activating Nrf2 ↑Nrf2 activation; ↑AMPK and ACC phosphorylation | | | | ^51^ | | | |
| 3.2 Neddylation in fatty liver disease | | | | | | | | | | | | | | | | | | | | | | | | | | | | | | |
| 54 | | | | MLN4924 | NEDD8-activating enzyme (NAE) inhibitor | | | | | | C57BL/6 mice | | | | | | | | | 60 mg/kg every 4 days for 2 or 3 weeks | | | | | ↓hepatic steatosis; serum alanine aminotransferase (ALT) and aspartate aminotransferase (AST) ; ↓neddylation; ↓oxidative stress, lipid peroxidation, and inflammation | | ^52^ | | | |
| 4.1 Phosphorylation in hyperlipidemia and atherosclerosis | | | | | | | | | | | | | | | | | | | | | | | | | | | | | | |
| 55 | | | | TG101348 (fedratinib) | JAK2 inhibitor | | | | | | C57BL/6 mice;  *ApoE*^-/-^ mice | | | | | | | | | 120 mg/kg/d in the first week, and 240 mg/kg/d in the following days for 30 days | | | | | ↓atherogenesis in ApoE^-/-^mice; ↓HSPC expansion, excessive myelopoiesis, and leukocytosis | | ^53^ | | | |
| 56 | | | | U0126 | MEK1/2 inhibitor | | | | | | *Ldlr*^-/-^ mice | | | | | | | | | 3 mg/kg/d for 20 weeks | | | | | ↓plaque lesion; ↑ApoE expression in LDLR^-/-^ mice | | ^54^ | | | |
| 57 | | | | Ruxolitinib | JAK1/2 inhibitor | | | | | | New Zealand male rabbits | | | | | | | | | Unknown dose for 12 weeks | | | | | ↓the area of atherosclerotic plaques; ↓aortic intimal injury; ↓mineralized nodules; ↓plasma lipids levels and plaques lipid burden; ↓plasma IL-6, IL-1β, IFN-γ and TNF-α levels; ↓JAK2 and STAT3 phosphorylation; ↓SOCS3 expression; ↑IL-17 and IL-10 levels | | ^55^ | | | |
| 58 | | | | TG101348 | JAK2 inhibitor | | | | | | *ApoE*^-/-^ mice | | | | | | | | | 120 mg/kg/d for first weeks | | | | | ↓monocytosis and neutrophilia in WD-fed ApoE^-/-^ mice; ↓the splenomegaly; ↓atherosclerosis by suppression of HSPC expansion, excessive myelopoiesis, and leukocytosis; ↓cellular phosphorylated STAT5 and ERK1/2 | | ^53^ | | | |
| 60 | | | | SP600125 | JNK inhibitor | | | | | | *ApoE*^-/-^ mice | | | | | | | | | 0.2 mg/kg/d for 10 days | | | | | ↓low shear stress–induced atherosclerotic plaque formation; ↑induced VCAM-1 expression | | ^56^ | | | |
| 61 | | | | Trodusquemine | Protein-tyrosine phosphatase 1B (PTP1B) inhibitor | | | | | | *ob/ob* mice | | | | | | | | | 10 mg/kg/d for 1 time or 6 weeks | | | | | ↓obesity-induced increase in atherosclerotic plaque area; ↓hyperphosphorylation of aortic Akt and AMPKα1 | | ^57^ | | | |
| 4.2 Acetylation in hyperlipidemia and atherosclerosis | | | | | | | | | | | | | | | | | | | | | | | | | | | | | | |
| 62 | | | | TMP195 | | Selective class IIa histone deacetylase (HDAC) inhibitor | | | | | | | | | *ApoE*^-/-^ mice | | | | | | 50 mg/kg/d for 4 weeks | | | | | ↓endothelial activation as demonstrated by mitigated VCAM-1 expression; ↓the initiation of atherosclerosis; ↓HDAC9 | ^58^ | | | |
| 63 | | | | Suberoylanilide hydroxamic acid (SAHA) | | Pan-inhibitor of HDAC1, HDAC2 and HDAC3 (Class I), HDAC6 and HDAC7 (Class II) and HDAC11 (Class IV) | | | | | | | | | *ApoE*^-/-^ mice | | | | | | 10 mg/kg every other day for 4 weeks | | | | | ↓atherosclerotic plaque area along the aorta; ↓aortic CD68 and CD45 protein levels; ↓Nox1, Nox4 protein levels and markers of inflammation in the atherosclerotic aorta | ^59^ | | | |
| 64 | | | | Trichostatin A | | HDAC class I/II inhibitor | | | | | | | | | *ApoE*^-/-^ mice | | | | | | 0.5 mg/kg/d for 12 weeks | | | | | ↓foam cell formation and enhances the expression of PPARγ; ↑PPARγ and relieves AS in mice; ↑PPARγ mainly through C/EBPα acetylation-associated transactivation | ^60^ | | | |
| 65 | | | | Trichostatin A | | HDAC class I/II inhibitor | | | | | | | | | *Ldlr* ^-/-^ mice | | | | | | 0.5 mg/kg/d for 4 weeks | | | | | ↓IL-6 and IL-1β; ↑macrophage accumulation in aortic sinus; ↑histone hyperacetylation; ↑the expression of CD36, increased SRA, TNF-α, and VCAM-1 | ^61^ | | | |
| 4.3 Ubiquitination in hyperlipidemia and atherosclerosis | | | | | | | | | | | | | | | | | | | | | | | | | | | | | | |
| 66 | | | | PYR-41 | | Ubiquitin-activating enzyme E1 inhibitor | | | | | | | | | *ApoE*^-/-^ mice | | | | | | 10 mg/kg for two times a week | | | | | ↓the atherosclerosis burden in the entire inner surface of the aortas and the aortic root; ↓macrophage infiltration and plaque necrosis; ↓the aortic expression of Mcp-1, Vcam-1, and Icam-1; Il-1β and Il-6 —the main metabolic parameters | ^62^ | | | |
| 67 | | | | Bortezomib | | 20S proteasome inhibitor | | | | | | | | | *ApoE* ^-/-^ mice | | | | | | 0.1 or 0.5 μg/g once every 3 days for 4 weeks | | | | | ↓α-SMC-actin; ↓proteasome; ↑in cleaved caspase-3; ↑enlargement of the necrotic core | ^63^ | | | |
| 68 | | | | Bortezomib | | 20S proteasome inhibitor | | | | | | | | | *ApoE* ^-/-^ mice | | | | | | 50 μg/kg for twice weekly | | | | | ↓chymotrypsin-like proteasomal activity; ↓collagen content in the brachiocephalic artery (BCA) lesions | ^64^ | | | |
| 69 | | | | Aspirin | | NF-κB inhibitor | | | | | | | | | New Zealand rabbits | | | | | | 100 mg/kg/d for 12 weeks | | | | | ↓IκBα, pIκBα and p65; ↓the pathogenesis of atheroma formation | ^65^ | | | |
| 4.4 S-Nitrosylation in hyperlipidemia and atherosclerosis | | | | | | | | | | | | | | | | | | | | | | | | | | | | | | |
| 70 | | | Probucol and cilostazol | | | Phosphodiesterase (PDE) 3A inhibitor | | | | | | | | | Japanese white rabbits | | | | | | 0.3% PB and 0.3% CZ 100 g/rabbit/d for 16 weeks | | | | | ↓the lesion areas; ↓the macrophage accumulation and smooth muscle cell proliferation in the lesions; ↑the levels of NO and protein S-nitrosylation; ↑anti-oxidant and anti-inflammatory effects | ^66^ | | | |

**Reference：**

1 Zhou, G. *et al.* Role of AMP-activated protein kinase in mechanism of metformin action. *J Clin Invest* **108**, 1167-1174 (2001).

2 Lai, D. *et al.* The Rho kinase inhibitor, fasudil, ameliorates diabetes-induced cardiac dysfunction by improving calcium clearance and actin remodeling. *J Mol Med (Berl)* **95**, 155-165 (2017).

3 Medicherla, S. *et al.* Preventive and therapeutic potential of p38 alpha-selective mitogen-activated protein kinase inhibitor in nonobese diabetic mice with type 1 diabetes. *J Pharmacol Exp Ther* **318**, 99-107 (2006).

4 Ito, Y. *et al.* Therapeutic effects of the allosteric protein tyrosine phosphatase 1B inhibitor KY-226 on experimental diabetes and obesity via enhancements in insulin and leptin signaling in mice. *J Pharmacol Sci* **137**, 38-46 (2018).

5 Wang, T. *et al.* The MEK inhibitor U0126 ameliorates diabetic cardiomyopathy by restricting XBP1's phosphorylation dependent SUMOylation. *Int J Biol Sci* **17**, 2984-2999 (2021).

6 Dey, A., Hao, S., Wosiski-Kuhn, M. & Stranahan, A. M. Glucocorticoid-mediated activation of GSK3beta promotes tau phosphorylation and impairs memory in type 2 diabetes. *Neurobiol Aging* **57**, 75-83 (2017).

7 Luo, W. *et al.* Inhibition of EGFR-STAT3 attenuates cardiomyopathy in streptozotocin-induced type 1 diabetes. *J Endocrinol* **242**, 199-210 (2019).

8 Wang, Z. J. *et al.* A Novel Rhynchophylline Analog, Y396, Inhibits Endothelial Dysfunction Induced by Oxidative Stress in Diabetes Through Epidermal Growth Factor Receptor. *Antioxid Redox Signal* **32**, 743-765 (2020).

9 Shibata, T., Takaguri, A., Ichihara, K. & Satoh, K. Inhibition of the TNF-alpha-induced serine phosphorylation of IRS-1 at 636/639 by AICAR. *J Pharmacol Sci* **122**, 93-102 (2013).

10 Galan, M. *et al.* A novel role for epidermal growth factor receptor tyrosine kinase and its downstream endoplasmic reticulum stress in cardiac damage and microvascular dysfunction in type 1 diabetes mellitus. *Hypertension* **60**, 71-80 (2012).

11 Fukuda, S. *et al.* Pharmacological profiles of a novel protein tyrosine phosphatase 1B inhibitor, JTT-551. *Diabetes Obes Metab* **12**, 299-306 (2010).

12 Westermann, D. *et al.* Inhibition of p38 mitogen-activated protein kinase attenuates left ventricular dysfunction by mediating pro-inflammatory cardiac cytokine levels in a mouse model of diabetes mellitus. *Diabetologia* **49**, 2507-2513 (2006).

13 Noh, H. *et al.* Histone deacetylase-2 is a key regulator of diabetes- and transforming growth factor-beta1-induced renal injury. *Am J Physiol Renal Physiol* **297**, F729-739 (2009).

14 Gilbert, R. E. *et al.* Histone deacetylase inhibition attenuates diabetes-associated kidney growth: potential role for epigenetic modification of the epidermal growth factor receptor. *Kidney Int* **79**, 1312-1321 (2011).

15 Advani, A. *et al.* Long-term administration of the histone deacetylase inhibitor vorinostat attenuates renal injury in experimental diabetes through an endothelial nitric oxide synthase-dependent mechanism. *Am J Pathol* **178**, 2205-2214 (2011).

16 Sun, X. Y. *et al.* Valproate attenuates diabetic nephropathy through inhibition of endoplasmic reticulum stressinduced apoptosis. *Mol Med Rep* **13**, 661-668 (2016).

17 Khan, S. & Jena, G. Sodium butyrate, a HDAC inhibitor ameliorates eNOS, iNOS and TGF-beta1-induced fibrogenesis, apoptosis and DNA damage in the kidney of juvenile diabetic rats. *Food Chem Toxicol* **73**, 127-139 (2014).

18 Khan, S., Jena, G. & Tikoo, K. Sodium valproate ameliorates diabetes-induced fibrosis and renal damage by the inhibition of histone deacetylases in diabetic rat. *Exp Mol Pathol* **98**, 230-239 (2015).

19 Lee, H. A. *et al.* Histone deacetylase inhibitor MGCD0103 protects the pancreas from streptozotocin-induced oxidative stress and beta-cell death. *Biomed Pharmacother* **109**, 921-929 (2019).

20 Chen, H. *et al.* Histone demethylase UTX is a therapeutic target for diabetic kidney disease. *J Physiol* **597**, 1643-1660 (2019).

21 Gao, C. *et al.* MG132 ameliorates kidney lesions by inhibiting the degradation of Smad7 in streptozotocin-induced diabetic nephropathy. *J Diabetes Res* **2014**, 918396 (2014).

22 Li, X. *et al.* MF-094, a potent and selective USP30 inhibitor, accelerates diabetic wound healing by inhibiting the NLRP3 inflammasome. *Exp Cell Res* **410**, 112967 (2022).

23 Chen, C. *et al.* Cullin neddylation inhibitor attenuates hyperglycemia by enhancing hepatic insulin signaling through insulin receptor substrate stabilization. *Proc Natl Acad Sci U S A* **119** (2022).

24 Long, C. A. *et al.* Poly-ADP-ribose-polymerase inhibition ameliorates hind limb ischemia reperfusion injury in a murine model of type 2 diabetes. *Ann. Surg.* **258**, 1087-1095 (2013).

25 Krishnan, N., Konidaris, K. F., Gasser, G. & Tonks, N. K. A potent, selective, and orally bioavailable inhibitor of the protein-tyrosine phosphatase PTP1B improves insulin and leptin signaling in animal models. *J Biol Chem* **293**, 1517-1525 (2018).

26 Li, J. *et al.* Phosphorylation of eIF2alpha signaling pathway attenuates obesity-induced non-alcoholic fatty liver disease in an ER stress and autophagy-dependent manner. *Cell Death Dis* **11**, 1069 (2020).

27 Mowers, J. *et al.* Inflammation produces catecholamine resistance in obesity via activation of PDE3B by the protein kinases IKKepsilon and TBK1. *Elife* **2**, e01119 (2013).

28 Zhang, L. *et al.* Sodium Butyrate Protects -Against High Fat Diet-Induced Cardiac Dysfunction and Metabolic Disorders in Type II Diabetic Mice. *J Cell Biochem* **118**, 2395-2408 (2017).

29 Lantz, K. A. *et al.* Inhibition of PTP1B by trodusquemine (MSI-1436) causes fat-specific weight loss in diet-induced obese mice. *Obesity (Silver Spring)* **18**, 1516-1523 (2010).

30 Oh, S. *et al.* Antidiabetic and antiobesity effects of Ampkinone (6f), a novel small molecule activator of AMP-activated protein kinase. *J Med Chem* **53**, 7405-7413 (2010).

31 Ma, J. *et al.* SAHA induces white fat browning and rectifies metabolic dysfunctions via activation of ZFPs. *J Endocrinol* **249**, 177-193 (2021).

32 Sharma, S. & Taliyan, R. Epigenetic modifications by inhibiting histone deacetylases reverse memory impairment in insulin resistance induced cognitive deficit in mice. *Neuropharmacology* **105**, 285-297 (2016).

33 Cakir, I. *et al.* Histone deacetylase 6 inhibition restores leptin sensitivity and reduces obesity. *Nat Metab* **4**, 44-59 (2022).

34 Lv, X. *et al.* HDAC inhibitor Trichostatin A suppresses adipogenesis in 3T3-L1 preadipocytes. *Aging (Albany NY)* **13**, 17489-17498 (2021).

35 Ferrari, A. *et al.* Attenuation of diet-induced obesity and induction of white fat browning with a chemical inhibitor of histone deacetylases. *Int J Obes (Lond)* **41**, 289-298 (2017).

36 Bele, S. *et al.* MS-275, a class 1 histone deacetylase inhibitor augments glucagon-like peptide-1 receptor agonism to improve glycemic control and reduce obesity in diet-induced obese mice. *Elife* **9** (2020).

37 Yoon, G. E., Jung, J. K., Lee, Y. H., Jang, B. C. & In Kim, J. Histone deacetylase inhibitor CG200745 ameliorates high-fat diet-induced hypertension via inhibition of angiotensin II production. *Naunyn Schmiedebergs Arch Pharmacol* **393**, 491-500 (2020).

38 Chu, X. Y. *et al.* Identification of Dacinostat as a potential anti-obesity compound through transcriptional activation of adipose thermogenesis in mice. *Biochim Biophys Acta Mol Basis Dis* **1867**, 166169 (2021).

39 Gaur, V. *et al.* Scriptaid enhances skeletal muscle insulin action and cardiac function in obese mice. *Diabetes Obes Metab* **19**, 936-943 (2017).

40 Jiang, X., Ye, X., Guo, W., Lu, H. & Gao, Z. Inhibition of HDAC3 promotes ligand-independent PPARgamma activation by protein acetylation. *J Mol Endocrinol* **53**, 191-200 (2014).

41 Zhang, W. *et al.* Muscular G9a Regulates Muscle-Liver-Fat Axis by Musclin Under Overnutrition in Female Mice. *Diabetes* **69**, 2642-2654 (2020).

42 Wu, X. *et al.* GSK126 alleviates the obesity phenotype by promoting the differentiation of thermogenic beige adipocytes in diet-induced obese mice. *Biochem Biophys Res Commun* **501**, 9-15 (2018).

43 Wei, Y. *et al.* Restoration of H3k27me3 Modification Epigenetically Silences Cry1 Expression and Sensitizes Leptin Signaling to Reduce Obesity-Related Properties. *Adv Sci (Weinh)* **8**, 2004319 (2021).

44 Lu, X. Y. *et al.* Feeding induces cholesterol biosynthesis via the mTORC1-USP20-HMGCR axis. *Nature* **588**, 479-484 (2020).

45 Park, H. S. *et al.* PPARgamma neddylation essential for adipogenesis is a potential target for treating obesity. *Cell Death Differ* **23**, 1296-1311 (2016).

46 Chen, X. Y. *et al.* LB100 ameliorates nonalcoholic fatty liver disease via the AMPK/Sirt1 pathway. *World J Gastroenterol* **25**, 6607-6618 (2019).

47 Yu, H. M., Chung, H. K. & Park, K. S. The PDE5 inhibitor udenafil ameliorates nonalcoholic fatty liver disease by improving mitochondrial function. *Biochem Biophys Res Commun* **558**, 57-63 (2021).

48 Elsayed, H. R. H. *et al.* Can Dasatinib Ameliorate the Hepatic changes, Induced by Long Term Western Diet, in Mice? *Ann Anat* **234**, 151626 (2021).

49 Lee, H. J. *et al.* Peroxisome proliferator-activated receptor-delta agonist ameliorated inflammasome activation in nonalcoholic fatty liver disease. *World J Gastroenterol* **21**, 12787-12799 (2015).

50 Hwang, H. J. *et al.* A dipeptidyl peptidase-IV inhibitor improves hepatic steatosis and insulin resistance by AMPK-dependent and JNK-dependent inhibition of LECT2 expression. *Biochem Pharmacol* **98**, 157-166 (2015).

51 Lee, D. H. *et al.* Ezetimibe, an NPC1L1 inhibitor, is a potent Nrf2 activator that protects mice from diet-induced nonalcoholic steatohepatitis. *Free Radic Biol Med* **99**, 520-532 (2016).

52 Serrano-Macia, M. *et al.* Neddylation inhibition ameliorates steatosis in NAFLD by boosting hepatic fatty acid oxidation via the DEPTOR-mTOR axis. *Mol Metab* **53**, 101275 (2021).

53 Tang, Y. *et al.* Inhibition of JAK2 Suppresses Myelopoiesis and Atherosclerosis in Apoe(-/-) Mice. *Cardiovasc Drugs Ther* **34**, 145-152 (2020).

54 Yang, J. *et al.* Combination of MEK1/2 inhibitor and LXR ligand synergistically inhibit atherosclerosis in LDLR deficient mice. *Biochem Biophys Res Commun* **522**, 512-517 (2020).

55 Yang, X. *et al.* Inhibition of JAK2/STAT3/SOCS3 signaling attenuates atherosclerosis in rabbit. *BMC Cardiovasc Disord* **20**, 133 (2020).

56 Wang, J. *et al.* Inhibition of c-Jun N-terminal kinase attenuates low shear stress-induced atherogenesis in apolipoprotein E-deficient mice. *Mol Med* **17**, 990-999 (2011).

57 Thompson, D. *et al.* Pharmacological inhibition of protein tyrosine phosphatase 1B protects against atherosclerotic plaque formation in the LDLR(-/-) mouse model of atherosclerosis. *Clin Sci (Lond)* **131**, 2489-2501 (2017).

58 Asare, Y. *et al.* Histone Deacetylase 9 Activates IKK to Regulate Atherosclerotic Plaque Vulnerability. *Circul. Res.* **127**, 811-823 (2020).

59 Manea, S. A. *et al.* Pharmacological inhibition of histone deacetylase reduces NADPH oxidase expression, oxidative stress and the progression of atherosclerotic lesions in hypercholesterolemic apolipoprotein E-deficient mice; potential implications for human atherosclerosis. *Redox Biol* **28**, 101338 (2020).

60 Chen, L. *et al.* HDAC3 inhibitor suppresses endothelial-to-mesenchymal transition via modulating inflammatory response in atherosclerosis. *Biochem Pharmacol* **192**, 114716 (2021).

61 Choi, J. H. *et al.* Trichostatin A exacerbates atherosclerosis in low density lipoprotein receptor-deficient mice. *Arterioscler Thromb Vasc Biol* **25**, 2404-2409 (2005).

62 Liao, J. *et al.* Inhibition of the Ubiquitin-Activating Enzyme UBA1 Suppresses Diet-Induced Atherosclerosis in Apolipoprotein E-Knockout Mice. *J Immunol Res* **2020**, 7812709 (2020).

63 Van Herck, J. L. *et al.* Proteasome inhibitor bortezomib promotes a rupture-prone plaque phenotype in ApoE-deficient mice. *Basic Res Cardiol* **105**, 39-50 (2010).

64 Wilck, N. *et al.* The Effect of Low-Dose Proteasome Inhibition on Pre-Existing Atherosclerosis in LDL Receptor-Deficient Mice. *Int J Mol Sci* **18** (2017).

65 Tan, C., Li, Y., Tan, X., Pan, H. & Huang, W. Inhibition of the ubiquitin-proteasome system: a new avenue for atherosclerosis. *Clin. Chem. Lab. Med.* **44**, 1218-1225 (2006).

66 Chen, Y. *et al.* Probucol and cilostazol exert a combinatorial anti-atherogenic effect in cholesterol-fed rabbits. *Thromb Res* **132**, 565-571 (2013).
